# Linkage and Association Mapping of *Arabidopsis thaliana* Flowering Time in Nature

**DOI:** 10.1371/journal.pgen.1000940

**Published:** 2010-05-06

**Authors:** Benjamin Brachi, Nathalie Faure, Matt Horton, Emilie Flahauw, Adeline Vazquez, Magnus Nordborg, Joy Bergelson, Joel Cuguen, Fabrice Roux

**Affiliations:** 1 Laboratoire Génétique et Evolution des Populations Végétales, Unité Mixte de Recherche CNRS 8016, Université des Sciences et Technologies de Lille 1, Villeneuve d'Ascq, France; 2 Department of Ecology and Evolution, University of Chicago, Illinois, United States of America; 3 GMI – Gregor Mendel Institute of Molecular Plant Biology, Austrian Academy of Sciences, Vienna, Austria; North Carolina State University, United States of America

## Abstract

Flowering time is a key life-history trait in the plant life cycle. Most studies to unravel the genetics of flowering time in *Arabidopsis thaliana* have been performed under greenhouse conditions. Here, we describe a study about the genetics of flowering time that differs from previous studies in two important ways: first, we measure flowering time in a more complex and ecologically realistic environment; and, second, we combine the advantages of genome-wide association (GWA) and traditional linkage (QTL) mapping. Our experiments involved phenotyping nearly 20,000 plants over 2 winters under field conditions, including 184 worldwide natural accessions genotyped for 216,509 SNPs and 4,366 RILs derived from 13 independent crosses chosen to maximize genetic and phenotypic diversity. Based on a photothermal time model, the flowering time variation scored in our field experiment was poorly correlated with the flowering time variation previously obtained under greenhouse conditions, reinforcing previous demonstrations of the importance of genotype by environment interactions in *A. thaliana* and the need to study adaptive variation under natural conditions. The use of 4,366 RILs provides great power for dissecting the genetic architecture of flowering time in *A. thaliana* under our specific field conditions. We describe more than 60 additive QTLs, all with relatively small to medium effects and organized in 5 major clusters. We show that QTL mapping increases our power to distinguish true from false associations in GWA mapping. QTL mapping also permits the identification of false negatives, that is, causative SNPs that are lost when applying GWA methods that control for population structure. Major genes underpinning flowering time in the greenhouse were not associated with flowering time in this study. Instead, we found a prevalence of genes involved in the regulation of the plant circadian clock. Furthermore, we identified new genomic regions lacking obvious candidate genes.

## Author Summary

Dissecting the genetic bases of adaptive traits is of primary importance in evolutionary biology. In this study, we combined a genome-wide association (GWA) study with traditional linkage mapping in order to detect the genetic bases underlying natural variation in flowering time in ecologically realistic conditions in the plant *Arabidopsis thaliana*. Our study involved phenotyping nearly 20,000 plants over 2 winters under field conditions in a temperate climate. We show that combined linkage and association mapping clearly outperforms each method alone when it comes to identifying true associations. This highlights the utility of combining different methods to localize genes involved in complex trait natural variation. Most candidate genes found in this study are involved in the regulation of the plant circadian clock and, surprisingly, were not associated with flowering time scored under greenhouse conditions. While rapid advances have been made in high-throughput genotyping and sequencing, high-throughput phenotyping of complex traits under natural conditions will be the next challenge for dissecting the genetic bases of adaptive variation in “laboratory” model organisms.

## Introduction

Flowering time is a major trait in the plant's life cycle, as it corresponds to the transition from the vegetative growth phase to the reproductive phase. At flowering, resources accumulated in storage tissues during the vegetative growth phase are reallocated to the production of seeds. Optimizing reproduction requires that the flowering date matches environmental conditions so that seeds can mature and disperse when conditions are appropriate. Natural variation in flowering time is related to latitude in many species [1]–[5], suggesting that factors such as photoperiod and temperature, that vary over large geographical scales, are likely involved in selecting for this trait. At the same time, environmental factors such as herbivory that act on a smaller spatial scale have been implicated [6]. Flowering time is, thus, a complex trait shaped by selective pressures acting on very different spatial scales.

A major goal in evolutionary biology is to identify the genetic basis of adaptive trait variation. For flowering time, many such studies have focused on the model species *Arabidopsis thaliana*. This species is a convenient choice because it is an annual plant with a worldwide distribution and, as such, encounters a variety of ecological conditions. Not surprisingly, diverse flowering time phenotypes have been described that likely result from different selective events across its range [3], [7]. In *A. thaliana*, flowering time is regulated by a complex genetic network composed of four main converging pathways [8]: the vernalization pathway, the photoperiod pathway, the autonomous pathway and the gibberellin pathway. These pathways integrate environmental and physiological factors such as photoperiod variation, ambient temperature, vernalization, and plant growth in order to trigger the transition to flowering at an appropriate time [9].

Most studies aiming to unravel the genetics of flowering time variation were performed in greenhouse conditions [10 for a complete review]. The polymorphisms revealed in such studies are likely to be, at least in part, specific to greenhouse conditions that plants do not experience in nature. For example, Weinig *et al.* [11] used a recombinant inbred line (RIL) family to show that a substantial number of quantitative trait loci (QTL) detected in natural conditions could not be detected under controlled conditions. In Li *et al.* [12], another RIL family was phenotyped in growth chambers simulating climatic conditions in Sweden or Spain and significant QTL×environment interactions were found. Ecologically realistic conditions expose plants to a great number of signals from their environment; this might lead to the identification of genes other than those responsible for flowering time variation under greenhouse conditions.

Conventional linkage mapping can be an effective tool for identifying genes underlying natural variation. However, the genes identified by this method are restricted to the ones segregating in the cross under consideration. Genome-wide association (GWA) mapping overcomes this limitation, and has recently been shown to successfully reveal common variants responsible for the variation in 107 phenotypes in a set of *A. thaliana* natural accessions [13]. However, GWA mapping suffers from the limitation that it generates false positives due to population structure [14]–[16]. As shown in Atwell *et al.* [13], the expected false positive rate varies greatly depending on the phenotype. Flowering time-related phenotypes, for example, exhibit the highest number of significant associations across a range of thresholds, and most likely include a high number of false positives due to clear geographical structure in these phenotypes. Statistical methods to control for population structure have been developed to reduce the inflation of false positives associations [13], [17], [18], but an alternative is the complementary use of traditional linkage mapping in controlled crosses and the use of near-isogenic lines [10], [18], [19]. The complementarity of GWA mapping and classical linkage mapping was particularly well demonstrated in a mouse GWA mapping study where the *Lasc1* gene, suggested by GWA to be a functional element associated with susceptibility to lung tumors, could not be validated in two independent intercross mouse populations containing both *Lasc1* alleles [20].

The aim of this study is to identify the polymorphisms underlying natural variation of flowering time in *A. thaliana*. To achieve this objective, we phenotyped nearly 20,000 plants over 2 winters under field conditions, including 197 worldwide natural accessions, 4366 RILs derived from 13 independent crosses chosen to maximize genetic and phenotypic diversity [21], and near-isogenic lines (NILs) derived from one of the 13 RIL families. The use of 4366 RILs provides unprecedented power for dissecting the genetic architecture of flowering time in *A. thaliana*. Additional resolution is achieved by combining GWA statistical methods that control for population structure with coarse mapping using RILs and NILs. Together, these methods enhance our ability to distinguish true from false associations finely mapped by GWA mapping. Flowering time-related pathways are some of the best-characterized genetic networks in plants; we, thus, made use of this information to validate our method by detecting candidate genes determined *a priori*.

More than 60 additive QTLs, all with relatively small to medium effects, were detected in the field experiment. QTLs found by linkage mapping efficiently validate GWA results. Regions of the genome validated by QTLs are clearly enriched with highly associated SNPs as compared to the rest of the genome. This allows us to propose a list of candidate genes, most of which have never or rarely been detected to be associated with natural variation in flowering time under laboratory conditions [13].

## Results

### Natural variation of flowering time in ecologically realistic conditions

**Figure 1:**
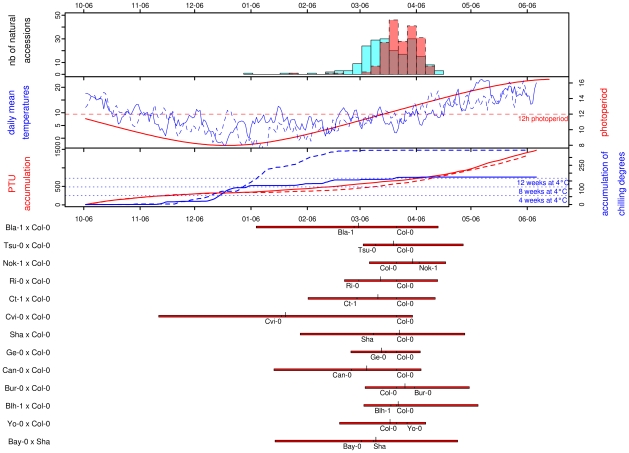
Distribution of natural variation for flowering time. For all frames of this figure, the x-axis gives the calendar dates from the 06^th^ of October (∼mean germination date) to the 06^th^ of June. The four panels in the figure are described from top to bottom. The top panel corresponds to the distribution of flowering time scored for the 184 natural accessions in 2007/2008 (blue) and 2008/2009 (red). The next panel gives the curves for photoperiod (in red), and the daily mean temperatures for 2007/2008 (blue solid line) and for 2008/2009 (blue dashed line). The next panel gives the photothermal units (PTU) accumulated from the beginning of germination to the end of the flowering season for 2007/2008 (red solid line) and for 2008/2009 (red dashed line). The accumulation of chilling degrees is represented over the same period (2007/2008: blue solid line, 2008/2009: blue dashed line). The equivalent accumulation in chilling degrees to 4, 8 and 12 weeks in a growth chamber at 4°C is indicated by blue dotted lines. The bottom panel gives the distribution of flowering time for each of the RIL families. For each RIL family, red bars extend from the minimum to the maximum values observed, with the larger ticks demarcating the median of the distribution and the smaller ticks indicating the flowering times for the parental lines.

Photothermal time is a temporal measure that integrates climatic conditions. Because flowering time may result from sensing climatic cues in *A. thaliana*, flowering time was scaled in photothermal units (PTU) using a phenology model that integrates both photoperiod length and temperature. To test the hypothesis that photothermal time is a better predictor of flowering time than Julian days [22], we grew 192 accessions in a field experiment over 2 consecutives winters. General patterns of daily mean temperature, daily photoperiod, accumulation of PTU, and accumulation of chilling degrees are presented for both years in Figure 1. In both years, the first natural accession (Pa-1 from Sicilia) flowered when photoperiod started to increase under short day conditions (∼8h and ∼9h photoperiod in the 2007/08 and 2008/09 experiments respectively). The last accessions (*i.e.* originating from Scandinavian regions) flowered when photoperiod reached approximately 14h photoperiod in both years (respectively on the 2008/04/19 and 2009/04/15), with the flowering peak occurring at around 12h photoperiod. The temperature curves indicate that plants endured much colder temperatures, over a longer period during winter 2008/2009 than during the previous year. January 2009 was continuously colder (mean temperature = 1.11°C) than January 2008 (mean temperature = 6.66°C); natural accessions therefore accumulated more chilling degrees (*i.e.* temperatures considered to vernalize the plant efficiently), and slightly less PTU during the second year. Accessions flowered later during the 2008/2009 experiment (median = March 28^th^ 2009) compared with the 2007/2008 experiment (median = March 21^th^ 2008) when flowering time is expressed in Julian days, although they accumulated less PTU (Figure 1).

A regression coefficient equal to 1 is expected if there is a perfect match of flowering time between the 2 years. This year-to-year comparison indicated that photothermal time seems to be a better predictor of flowering time than the number of Julian days (Figure S1): the regression coefficient for flowering time expressed in Julian days (slope = 0.63, CI = 0.60–0.65; intercept = 70.06, *P*<0.001; *R* ^2^ = 0.93) is significantly smaller than the regression coefficient for flowering time expressed in PTU (slope = 0.89, CI = 0.85–0.93; intercept = −72.32, *P*<0.001; *R* ^2^ = 0.91). Expressing flowering time in PTU improves the repeatability of the experiment, as the slope is closer to 1, but we can also note that the intercept for PTU is lower than zero. This earlier flowering when expressed in PTU might result from the colder period in January during the second year (2008/2009), resulting in a higher accumulation of chilling degrees in 2008/2009 compared to the 2007/2008 (Figure 1, see Discussion). Therefore, all flowering data from the set of 2007/2008 and 2008/2009 experiments were expressed in photothermal units (PTU).

Highly significant variation was observed among the set of 197 natural accessions (Table 1), with flowering time per accession averaging 457.37 PTU and ranging from 268.05 to 583.44 PTU (Figure S2). The distribution of flowering times for these natural accessions is roughly normal, mostly due to the transformation in photothermal units. When expressed in days, the distribution is L-shaped with a tail of early-flowering accessions (Figure 1). The shape of this distribution contrasts with other observations made for 16 flowering time related traits scored in various constant greenhouse conditions for the same set of natural accessions used in our study. In particular, Atwell and colleagues [13] found that a range of conditions including both long and short days combined with and without vernalization resulted in flowering related traits with either bimodal distributions or with L-shaped distributions containing a tail of late-flowering accessions. These 16 flowering time related traits scored in constant greenhouse conditions are more correlated with one another than with flowering time scored in our study [13].

**Table 1:** Summary statistics.

Plant material	Cross	RIL CRB code	Size	Mean	Range Days (min-max)	Range PTU (min-max)	*H* ^2^	V(G)/V(P)
Natural accessions	-	-	197	457.37	91.99–196.61	268.05–583.443	0.97	-
RIL parents	-	-	14	433.29	108.70–184.69	291.83–517.10	0.92	-
RIL families	Bla-1×Col-0	2RV	259	409.22	94.88–194.97	264.08–566.81	0.88	0.62
	Tsu-0×Col-0	3RV	276	469.85	153.73–208.77	414.58–662.74	0.84	0.62
	Nok-1×Col-0	4RV	223	505.35	145.87–199.12	388.32–590.31	0.79	0.52
	Ri-0×Col-0	6RV	286	446.30	143.42–194.69	379.39–565.29	0.71	0.33
	Ct-1×Col-0	7RV	377	441.58	123.41–193.37	326.12–558.43	0.73	0.51
	Cvi-0×Col-0	8RV	366	314.39	41.23–180.92	190.96–509.20	0.94	0.70
	Sha×Col-0	13RV	345	483.67	119.09–209.64	319.39–701.78	0.86	0.60
	Ge-0×Col-0	17RV	338	446.04	146.97–185.15	392.88–521.33	0.76	0.52
	Can-0×Col-0	19RV	371	420.50	104.64–182.40	280.68–522.84	0.86	0.63
	Bur-0×Col-0	20RV	343	489.67	154.79–212.13	416.79–685.34	0.85	0.55
	Blh-1×Col-0	21RV	315	478.57	154.10–216.94	417.81–700.04	0.77	0.31
	Yo-0×Col-0	23RV	456	460.53	140.72–188.14	371.00–534.14	0.79	0.62
	Bay-0×Sha	33RV	411	436.78	105.24–205.71	285.18–649.46	0.82	0.56

For each RIL family, the table gives the population phenotypic mean, the broad sense heritability (*H* ^2^), the proportion of phenotypic variation explained by the detected QTLs (V(G)/V(P)).

A significant positive relationship was found between flowering time and latitude (Figure S3; latitude regression coefficient ± SE = 3.19±0.40, *P*<0.0001). As previously noted [3], functionality at the flowering time gene *FRIGIDA* affected this positive relationship: accessions carrying a functional allele at FRI revealed a stronger latitudinal cline in flowering time (Table 1 in Dataset S1; latitude regression coefficient ± SE = 3.33±0.54, *P*<0.001) relative to accessions bearing a non-functional allele of FRI (latitude regression coefficient ± SE = 2.28±0.59, *P*<0.001). Indeed, the presence of this latitudinal cline in accessions carrying a non-functional allele was primarily due to Cvi-0, from the Cape Verde Islands, which is an outlier in the latitudinal distribution; if this outlier is removed, the relationship is no longer significant (latitude regression coefficient ± SE = 1.16±0.69, *P* = 0.097).

The parents of the RILs showed flowering times averaging 433.29 PTU and ranging from 291.83 (Cvi-0) to 517.10 PTU (Nok-3). These values span the distribution of natural accessions (Figure S2). Variation among the RIL parental lines covered 71.43% of the range observed among the 197 natural accessions. Twelve accessions are common to the parental lines and the natural accessions. Even though seeds from parental lines and natural accessions were produced in different environments, a highly significant correlation was observed for flowering time between these groups (Pearson correlation coefficient = 0.99, *P*<0.0001), suggesting that maternal effects do not impact flowering time in this study. The late-flowering behavior of the lab reference strain Col-0 (in our experiment 66.33% of all accessions flowered earlier than Col-0) is consistent with the results of Wilczek *et al.* [22], although it is generally described as an early-flowering accession when phenotyped under common laboratory conditions [13].

Highly significant variation for flowering time was observed among RILs within each RIL family (Figure S2, Table 2 in Dataset S1), with heritability of flowering time in RIL families averaging 0.82 and varying from 0.70 to 0.94 (Table 1). The range of observed flowering times among the 4366 RILs exceeds that observed among the 197 natural accessions at both sides of the distribution (Figure S2 and Figure S4). This was mainly due to significant extensive phenotypic transgression observed for each RIL family. Interestingly, only individuals from the RIL family Cvi-0×Col-0 started flowering under decreasing photoperiod. The intensity of phenotypic transgression was not related to the genetic relatedness of the corresponding parental lines (regression coefficient = −1.068, *P* = 0.957).

### Genetic architecture of flowering time

**Figure 2:**
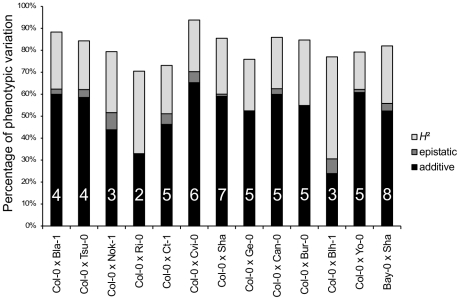
Broad-sense heritability, number of QTLs, and percentage of phenotypic variation explained by additive or epistatic QTLs for each of the 13 RIL families. *H* ^2^: broad-sense heritability (light grey bars). The percentage of phenotypic variation explained by additive and epistatic QTLs is illustrated by black and dark grey bars, respectively. The number of additive QTLs is indicated on the black bars.

**Figure 3:**
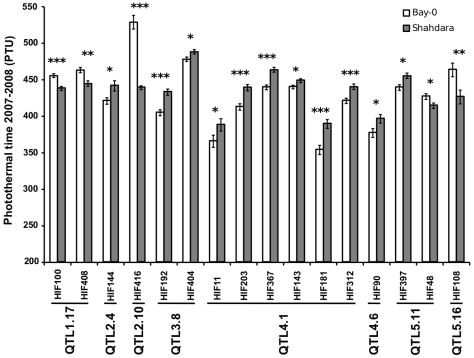
Validation of additive QTLs found in the Bay-0×Shahdara RIL family by NILs. Each of the 8 additive QTLs detected in the Bay-0×Shahdara RIL family is supported by 1 to 6 independent pairs of NILs (*i.e.* heterogeneous inbred families).

Sixty-two additive QTLs and 16 epistatic interactions were detected among the 13 RIL families (Tables 3 and 4 in Dataset S1, Figure S5), with each RIL family contributing between 2 (Ri-0×Col-0) and 8 (Bay-0×Shahdara) additive QTLs. The percentage of genetic variation explained by additive and epistatic QTLs in these RIL families averaged 66.46% and ranged from 39.71% (Blh-1×Col-0) to 78.5% (Yo-0×Col-0) (Figure 2). Each of the 8 additive QTLs detected in the Bay-0×Shahdara RIL family could be validated by 1 to 6 independent pairs of NILs, known as Heterogeneous Inbred Families (HIFs) (Figure 3, Tables 5 and 6 in Dataset S1), suggesting that additive QTLs detected by the mixed-model composite interval mapping approach are largely true positives. When more than one HIF was available, the allelic effect of each allele was consistent, except for QTL5.11 (Figure 3). In this case, a delay in flowering was associated with the Shadhara allele in HIF397 but with the Bay-0 allele in HIF48. Because QTL5.11 has been found to be in epistasis with another additive QTL (Figure 3: QTL5.16, Table 5 in Dataset S1), this inconsistency might be a consequence of the genetic background.

**Figure 4:**
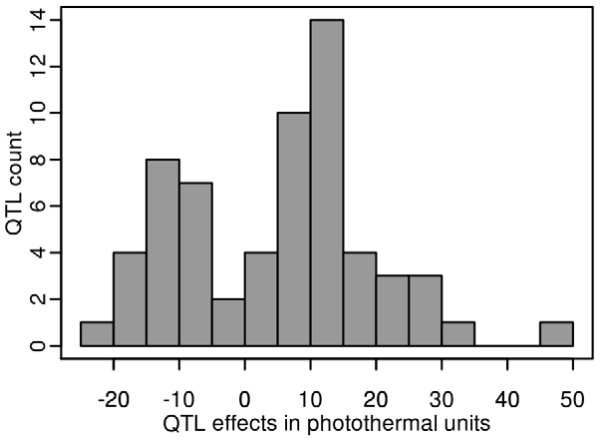
Distribution of the Col-0 additive allelic effect in the 2007–2008 field experiment. Histogram of additive allele estimates for the flowering time for all the 12 RIL families that have Col-0 as a common parental line relative to Col-0.

Given the large population size of each RIL family and the high heritability associated with flowering time, we were able to detect QTLs that account for a small percentage of phenotypic variation within each RIL family. The percentage of phenotypic variation explained by an additive QTL averaged 9.08% and ranged from below 1% (Tsu-0×Col-0, Ct-1×Col-0 and Bay-0×Shahdara) to 45.6% (Cvi-0×Col-0) (Figure 4). In a comparison of the 12 RIL families that have Col-0 as a common parental line, we found that the additive effects of Col-0 alleles range, in absolute values, from 4.19 to 48.09 photothermal units. As expected from the transgressive segregation observed within each RIL family, the distribution of the effects of the Col-0 allele is clearly bimodal, the first mode corresponding to QTLs having negative effects (the Col-0 allele makes plant flower earlier), and the second mode corresponding to QTLs having positive effects (the Col-0 allele makes plant flower later). A less stringent significance level for QTL detection and QTL effects (P = 0.10) did not affect the bimodal distribution of the Col-0 allele effects. Among the 54 additive QTLs detected in these 12 RIL families, 19 have negative effects (∼35.2%) and 35 have positive effects (∼64.8%).

Sixteen epistatic interactions were detected in 10 out of 13 RIL families, with each RIL family contributing 1 to 3 pairs of interacting QTLs. Epistatic interactions mainly involved QTLs with single-locus effects (10/16); only 1 epistatic interaction (Nok-1×Col-0) involved 2 QTLs without single-locus effects. The amount of phenotypic variation explained by epistatic QTLs ranged from 3.42 to 13.23 PTU. The proportion of phenotypic variation explained by these interactions averaged 2.42% and ranged from 0.09% to 7.8%; this is lower than the proportion of phenotypic variation explained by additive QTLs (Figure 2).

Our results were compared to those obtained for 5 RIL families common between this study and one by Simon *et al.* [23] (Blh-1×Col-0, Bur-0×Col-0, Ct-1×Col-0, Cvi-0×Col-0 and Shahdara×Col-0) by reanalyzing the flowering time data. Among those 5 RIL families, Simon *et al.* [23] found 22 additive QTLs when phenotyping plants under long days in a greenhouse experiment after a seeds vernalization period of 3 weeks. In the present study, considering the same 5 RIL families, 26 additive QTLs were detected, of which 10 (∼39%) have support intervals that overlap with support intervals of QTLs found in Simon *et al.* ([23], Table 7 in Dataset S1). Although the direction of the Col-0 allelic effect is consistent for all overlapping QTLs (Table 7 in Dataset S1), large differences in the predicted genetic position were observed for overlapping QTLs (mean = 3.44 cM±5.18 cM). This last result indicates that one cannot rule out different genetic bases for overlapping QTLs (see subsection Combining GWA mapping and traditional linkage mapping).

### Population structure and minor allele frequency dependence

The p-value distribution from the Wilcoxon rank-sum analyses showed an excess of low p-values, suggesting a potentially high rate of false positive associations. As expected for an excess of low p-values due to confounding by population structure, a mixed model approach that takes genetic similarity among natural accessions into account (EMMA method, ) effectively eliminated the excess of low p-values. While it might be tempting to consider p-values that remain extreme after EMMA correction as true associations, we need to keep in mind that minor allele frequency (MAF) also influences p-values [13], [24]. EMMA demonstrates enrichment in low p-values for rare alleles, whereas the Wilcoxon test showed weak power to detect associations for SNPs with low MAF (Figure S7). Comparison of the distribution of the p-values obtained by EMMA to a theoretical uniform distribution showed that the confounding remaining after the correction for population structure is likely to come from the bias due to rare alleles (Figure S6). Therefore, following Kang *et al.* [24], only SNPs with MAF>10% were considered further.

### Combining GWA mapping and traditional linkage mapping

**Figure 5:**
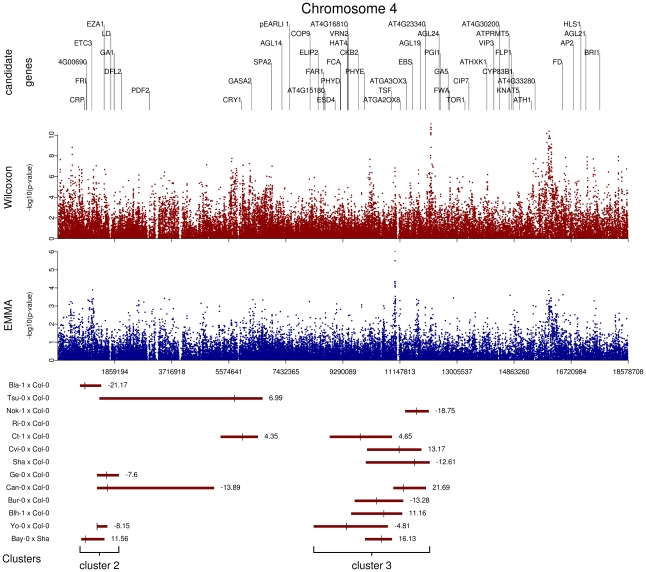
Comparison of GWA and traditional linkage mapping (additive QTLs) results for flowering time for chromosome 4. The *x*-axis indicates the physical position along the chromosome. Top panel: Position of the 52 *a priori* candidate genes located on chromosome 4. Mid-panel: −log^10^ p-values from a chromosome 4-wide scan using either the Wilcoxon model or the EMMA method (blue and red dots, respectively). Bottom panel: QTL regions for each of the 13 RIL families. For each RIL family, green bars represent the 95% confidence interval for QTL position, with the bigger tick representing the QTL position. QTL clusters 2 and 3 are highlighted below the corresponding QTL regions.

We initially inspected our data by identifying genomic regions in which highly significant SNPs within 20 kb of known *a priori* candidate genes for flowering time overlapped with QTL regions. These plots revealed several interesting features (additive QTLs: Figure 5 and Figure S8; epistatic QTLs: Figure S9). First, the 62 additive QTLs identified in the 13 RIL families are organized in 5 major clusters: cluster 1 is at the end of chromosome 1, cluster 2 is at the top of chromosome 4, cluster 3 is near the centromeric region of chromosome 4 (Figure 5), cluster 4 is at the beginning of chromosome 5, and cluster 5 is at the end of chromosome 5. In a similar field experiment, QTLs belonging to clusters 1, 3, and 5 have also been found in the Columbia×Landsberg *erecta* RIL family when scored for bolting time in fall in North Carolina and Rhode Island [11]. Second, allelic effects for QTLs in clusters 1, 2, and 5 were consistent among RIL families, whereas allelic effects for QTLs in clusters 3 and 4 were not. Third, p-values for SNPs within QTL regions were significantly smaller than p-values for SNPs located outside QTL confidence intervals; this was true for both the EMMA method (Kruskall-Wallis chi-squared = 23.30, *P*<0.001) and the Wilcoxon test (Kruskall-Wallis chi-squared = 71.64, *P*<0.001). Fourth, it was common for several association peaks to aggregate within a QTL region (Figure 5). Finally, as illustrated by the centromeric region of chromosome 1 (see Figure S8), some association peaks detected by the Wilcoxon test that are strongly associated with flowering time and fall within QTL regions were poorly associated when using the EMMA method; this result suggests the existence of false negatives after controlling for population structure.

### Enrichment of candidate genes for highly significant associations validated by QTLs

**Figure 6:**
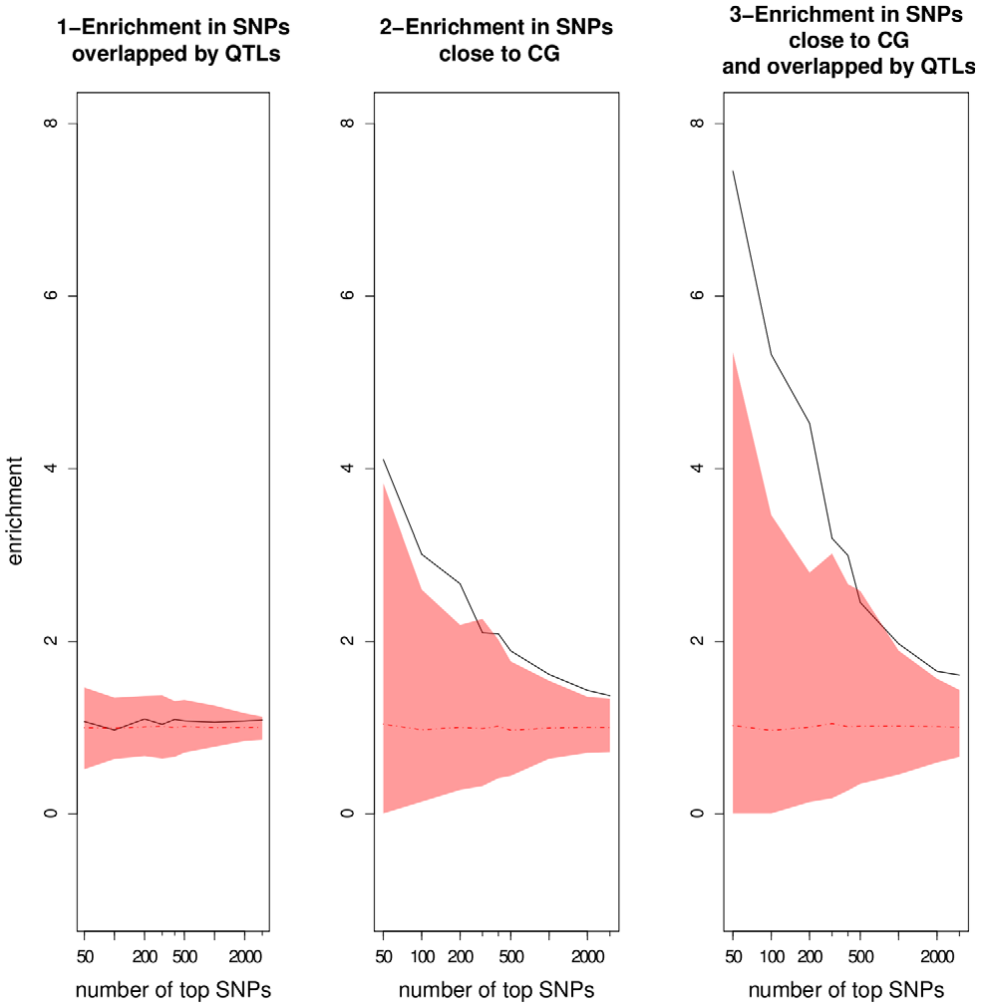
Enrichment ratios as a function of the number of top SNPs chosen in the GWA mapping results using the EMMA method. The mean and the corresponding 95% confidence interval from the null distributions are represented by the dotted line and the colored areas, respectively. CG: candidate gene.

We investigated the number of top SNPs that can be considered as true associations by looking at the enrichment ratios for progressively larger sets of top SNPs (Figure 6). The enrichment ratio for top SNPs validated by QTLs never differed from what would be expected randomly. This result underlines the fact that QTLs detected using the RILs are far too coarse to be informative by themselves. For top SNPs within 20 kb of candidate genes, the enrichment ratio dropped with the number of top SNPs, demonstrating that candidate genes are overrepresented among top-ranking SNPs. The enrichment ratio was clearly increased for SNPs close to candidate genes and overlapped by QTLs. For the 50 top SNPs, consideration of candidate genes overlapped by QTLs almost doubled the enrichment ratio relative to consideration of all candidate genes (7.4 *vs* 4.1). This suggests that combining QTLs with candidate genes is much more informative than using either approach separately.

Patterns are more difficult to interpret when not controlling for population structure. Indeed, enrichment in SNPs validated by both candidate genes and QTLs in the Wilcoxon analysis did not differ significantly from random, in particular for small sample sizes, indicating that a large proportion of the best associations are likely to be false positives (Table 8 in Dataset S1, Figure S10). Based on the results from EMMA, we consider the 500 top SNPs from both EMMA and Wilcoxon for plausible associations with candidate genes. This sample size corresponds to the limit of significance of enrichment ratio in EMMA (Figure 6).

### Plausible associations

Forty-two candidate genes, out of a list of 282 *a priori* candidate genes, were detected (Table 2). Among these, only 4 were detected with both EMMA and Wilcoxon, and were confirmed by a QTL: *TSF*, AT4G23340, *ELF*5, and *ETC3*. Eleven genes were detected with EMMA only and were overlapped by QTLs: *TOC1*, *ATHAP2B*, *YAP169*, *LD*, *FCA*, *PHYD*, AT2G39540, *KIN1*, *CDF3*, *KIN2*, and *COL1*. Ten genes were detected with Wilcoxon only and were overlapped by QTLs: *SRR1*, AT5G59570, *FY*, *GA1*, *MMP*, *ATGA2OX7*, *LKP2*, *CUL4*, *DDF1*, and *FPA*, suggesting that they are potentially false negatives generated by EMMA. These 10 genes represent a large fraction (*i.e.* 40%) of the 25 genes overlapped by QTLs. Finally, 17 candidate genes (40%) were detected by EMMA and/or Wilcoxon but were not overlapped by QTLs, suggesting that they are potentially false positives.

**Table 2:** List of candidate genes associated with flowering time scored in the field.

Genes	Gene ID	Chromosome	Overlapping QTLs^a^	EMMA^b^	Wilcoxon^b^	Top50^c^
TSF	AT4G20370	4	7,8,13,20,21,33	X	X	X
AT4G23340	AT4G23340	4	4,13,19	X	X	
ELF5	AT5G62640	5	2,17	X	X	X
ETC3	AT4G01060	4	2,33	X	X	
TOC1	AT5G61380	5	2,6,7,8,19,23,33	X		
ATHAP2B	AT3G05690	3	13,17,23	X		X
YAP169	AT5G07200	5	13,19,21	X		
LD	AT4G02560	4	3,17,19	X		X
FCA	AT4G16280	4	7,23	X		
PHYD	AT4G16250	4	7,23	X		
AT2G39540	AT2G39540	2	3,17	X		
KIN1	AT5G15960	5	20	X		
CDF3	AT3G47500	3	33	X		
KIN2	AT5G15970	5	20	X		
COL1	AT5G15850	5	20	X		
SRR1	AT5G59560	5	2,6,7,8,13,23,33		X	
AT5G59570	AT5G59570	5	2,6,7,8,13,23,33		X	
FY	AT5G13480	5	7,8,19,20		X	
GA1	AT4G02780	4	3,17,19		X	
MMP	AT1G70170	1	2,13,19		X	
ATGA2OX7	AT1G50960	1	3		X	
LKP2	AT2G18915	2	13		X	
CUL4	AT5G46210	5	33		X	
DDF1	AT1G12610	1	20		X	
FPA	AT2G43410	2	33		X	
AT2G30810	AT2G30810	2	-	X	X	
AGL 18	AT3G57390	3	-	X	X	
AT2G47310	AT2G47310	2	-	X	X	
ATH1	AT4G32980	4	-	X	X	
KNAT5	AT4G32040	4	-	X	X	
AT4G33280	AT4G33280	4	-	X	X	
AT3G57300	AT3G57300	3	-	X	X	
CKB1	AT5G47080	5	-	X		
DDF2	AT1G63030	1	-	X		
CCA1	AT2G46830	2	-	X		
AP1	AT1G69120	1	-	X		X
TOR1	AT4G27060	4	-	X		
GI	AT1G22770	1	-	X		
PMI15	AT5G38150	5	-	X		
GASA5	AT3G02885	3	-		X	
AGL16	AT3G57230	3	-		X	
AGL17	AT2G22630	2	-		X	

**a** Numbers refer to the RIL CRB code of the 13 RIL families (see Table 1).

**b** “X” indicates that the gene was detected among the 500 best associations with EMMA and/or Wilcoxon.

**c** “X” indicates that the gene was detected among the 50 best associations with EMMA.

Seventeen of these associated candidate genes were found for at least one of the 16 flowering time-related greenhouse phenotypes measured by 6 different teams, in different greenhouse conditions, and published in Atwell *et al.* (Table 9 in Dataset S1, [13]). Among these candidate genes, only 9 are supported by QTL confidence intervals.

*FRI* functionality did not associate significantly with the flowering time measured in the field over winter (with EMMA, −log^10^ p-value<2.4 in a 20 kb region on both side of *FRI*). To control for allelic heterogeneity [13], the GWA mapping analyses were also performed separately for accessions carrying functional *FRI* alleles and non-functional alleles (‘Ler’ allele or ‘Col’ allele). However, associations within a 40 kb window centered on *FRI* were not improved, and the maximum −log^10^ p-values using EMMA were 2.3 and 2.2 for the ‘Ler’ and ‘Col’ alleles, respectively. This is consistent with our failure to validate a role for *FRI* in RIL families for which the parents were segregating for functional and non-functional alleles (Table 1 in Dataset S1). Furthermore, the QTLs detected at the beginning of chromosome 4 in the Bay-0×Shahdara family were validated by 6 independent HIFs (Figure 3 and Table 5 in Dataset S1). Those 6 HIF lines segregate for only one common region between 0.41 and 2.58 Mb, which does not overlap with *FRI*. In this genomic region, only *ETC3*, shown to have an effect on flowering development [25], was detected among the top SNPs in EMMA (rank = 163), and could therefore be proposed as a candidate gene for natural variation of flowering time. Two major *FLC* haplogroups (*FLCA* and *FLCB*) have previously been found to be associated with flowering time variation in *A. thaliana* under field conditions [26], but only in the presence of putatively functional *FRI* alleles. In this study, we first excluded 40 accessions with a weak or non-functional *FLC* allele from the analyses (Table 1 in Dataset S1). When taking into account population structure, no significant haplogroup effect was detected when considering all accessions with an active *FLC* allele (−log^10^ p-value = 0.07) or when considering accessions with both an active *FLC* allele and a functional *FRI* allele (−log^10^ p-value = 0.62). A haplogroup effect was similarly not detected in an outbred population of *A. thaliana* that was phenotyped for flowering time in growth chambers simulating fall conditions [27], [28]. As for *FRI*, the absence of a haplogroup effect is consistent with our failure to validate a role of *FLC* in RIL families for which the parents segregate for *FLCA* and *FLCB* (Table 1 in Dataset S1). *PHYC*, another gene believed to be important based on greenhouse experiments [29], was not significantly associated with flowering time, and no QTL has been found in RIL families whose parental lines are polymorphic for *PHYC* (Figure S8, Table 1 in Dataset S1).

Flowers *et al.* [30] sequenced 52 candidate genes for flowering time in 24 natural accessions, all with the exception of Kas-2 were included in our 184 natural accessions used for GWA mapping analysis. Among these 52 genes, 5 are included in the set of 25 genes detected by GWA and overlapped by QTLs in our study: *TSF*, *ELF5*, *LD*, *PHYD*, and *GA1*. In *ELF5*, 3 common non-synonymous polymorphisms were detected among the 24 accessions sequenced by Flowers *et al.* [30]. The top SNP associated with flowering time in our study and close to *ELF5* (Chromosome 5, position = 25,175,269bp) is in strong linkage disequilibrium (LD) (*r* ^2^ = 0.82, Fisher's exact test: *P*<0.001) with the polymorphism responsible for the change of the amino-acid (AA) in position 503 (Ala→Thr). The Alanine residue in position 503 is conserved in the *ELF5* orthologous gene in *Arabidopsis lyrata*, suggesting that the Threonine residue is the derived state (The *Arabidopsis lyrata* genome project, http://genome.jgi-psf.org/Araly1/Araly1.home.html). LD between the significant marker and the other two polymorphisms (AA in position 364 and 393) are not significant (respectively, *r* ^2^ = 0.38, *P*<0.027; and *r* ^2^ = 0.07, *P*<0.526). AA in position 393 is in complete LD with SNP markers at positions 25,167,440 and 25,168,305 on chromosome 5 that were not found to be associated with flowering time. Unfortunately, the two RIL family founders that are common with Flowers *et al.*'s set of accessions, *i.e.* Cvi-0 and Ct-1, are not polymorphic for those 3 common *ELF5* non-synonymous polymorphisms. For *PHYD*, *GA1*, and *LD*, no significant LD was found between common non-synonymous polymorphisms detected by Flowers *et al.* [30] and the top SNPs detected in our study. In *TSF*, only 4 rare non-synonymous polymorphisms were found, making their detection by GWA mapping unlikely. Whereas QTLs detected in the RIL families Cvi-0×Col-0 and Ct-1×Col-0 did overlap with *TSF*, no amino-acid change was found between the founders. Recently, a causal polymorphism mediating natural variation in flowering time has been identified in the promoter of the closest homolog of *TSF*, *FLOWERING LOCUS T* [31], suggesting that the causal polymorphisms in *TSF* might also be located in its promoter region.

**Figure 7:**
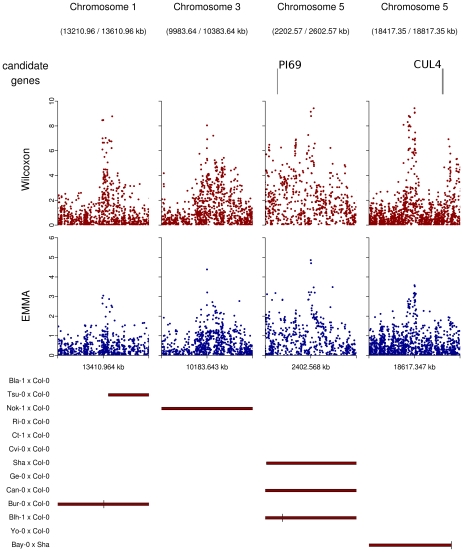
Peaks of associations with no candidate genes within a 20 kb region on either side of the top SNP (plotting window 400 kb).

Regions overlapped by QTLs but presenting no obvious candidate were graphically checked for neat association peaks in the EMMA and the Wilcoxon outputs. Nine regions showing neat peaks were selected and checked for obvious candidate genes that might have been missed when establishing our list of 282 *a priori* candidate genes (Table 10 in Dataset S1, Figure 7). Among those regions, only the region of 20 kb around position 18,617,347 on chromosome 5 included two genes of potential interest: *DOG1* involved in the dormancy processes and *SAG12* associated with leaf senescence. No obvious candidates were found in the other 8 regions, even when those regions were expanded to 50 kb (Figure 7).

## Discussion

### Natural variation and genetic architecture

In our simple model, photothermal units (PTU) significantly outperformed Julian days in terms of the reproducibility of the measure of flowering time. We chose to keep our model simple, rather than applying the genetically informed model of Wilczek *et al.* [22] that includes information about flowering requirements, such as critical day length, temperature, and winter chilling temperatures during vernalization. While consideration of vernalization could improve our photothermal model, the vernalization requirements are difficult to address for three reasons. First, the amount of vernalization required is likely to vary among natural accessions according to both the length of period to cold exposure [32] and the vegetative stage at which plants are exposed to cold [33, personal observation, F. Roux]. Second, Shindo *et al.* [32] showed that levels of *FLC*, a floral repressor responsive to vernalization that decreases with vernalization and thus releases initiation of flowering, may return to initial expressions levels when vernalization is not saturated. In our experiment, temperatures fluctuated within years such that cold exposure was not constant (Figure 1). This succession of vernalizing and non-vernalizing temperatures is very different from what plants may experience in growth chambers, and *FLC* expression may be reactivated when temperatures transitionally reach non-vernalizing levels. Third, compensatory effects between the different pathways determining flowering time have been showed in *A. thaliana* and in other plant species [34], [35], particularly between the photoperiod and the vernalization pathway. In the iteroparous herb *Beta vulgaris* ssp. *maritima*, a linear relationship was found between the photoperiod on flowering date and the vernalization duration; *i.e.* extra vernalization allowed the plants to flower under a shorter photoperiod [35]. In our experiment, plants flowered with fewer PTU in 2008/2009 than in 2007/2008, although they flowered slightly later in the season. This result suggests that other mechanisms might have compensated for the lack of accumulated PTU, such as stronger vernalization in 2008/2009. In these conditions, it is very difficult to make any assumptions about the amount of vernalization required for each natural accession but this possible explanation certainly deserves further exploration.

Great variability in flowering time was observed among the 14 accessions that served as parents in the RILs. This is not surprising because parents were chosen to maximize genetic and phenotypic diversity observed under greenhouse conditions [21]. An interesting point about these 14 crosses is the amount of transgressive segregation observed in each of the RILs. Transgression, defined as the generation of extreme phenotypes relative to the parental lines, could have originated due to 3 different phenomena [36]: (i) Expression of over- or under-dominance; (ii) epistatic interactions; or (iii) the complementarity of additive alleles that are dispersed between the parental lines. The first phenomenon is unlikely due to the low residual heterozygosity expected after 5–6 generations of selfing in the RILs. While epistatic interactions have been detected in some RIL families, the epistatic genetic variance is low compared to the additive genetic variance. This suggests that the transgression detected in this study most likely originates from the recombination of alleles with opposite effects. The absence of a negative relationship between the intensity of phenotypic transgression and the genetic relatedness between pairs of founders is inconsistent with the observed phenotypic transgression resulting from the break up of population structure but rather suggests that natural accessions contain locally co-adapted gene complexes. The absence of a negative relationship between the intensity of phenotypic transgression and the genetic relatedness between pairs of founders has also been found for 3 flowering time related traits in maize (data not shown; [37]). Assessing the extent of transgression in different species, and different traits that show contrasting patterns with regard to population structure, is needed to allow generalization about the cause of transgressive segregation.

The use of 4,366 RILs divided into 13 families provides a great opportunity to almost completely dissect the genetic architecture of flowering time in *A. thaliana* under our field conditions. Our field study revealed many QTLs of relatively moderate effect. This environmental dependence of the distribution of QTL effects might originate from the relatively larger number of environmental signals in outdoor conditions as compared to the greenhouse. The QTLs detected in the present study are nevertheless of greater effect than the QTLs detected in field trials on 25 RIL families in maize [37], and in mice, flies, and humans [38]. Perhaps a genetic architecture of small-effect QTLs is favored in outcrossing species as a means of ensuring synchronous flowering among plants within a local population [37]. Selfing species such as *A. thaliana*, on the other hand, do not require synchronous flowering. A comparative assessment of *FRI* further suggests that selfing species can tolerate large QTL effects on flowering. While non-functional *FRI* alleles are associated with additive effects on flowering time of up to 19.7 days for *A. thaliana* in greenhouse conditions (B. Brachi and F. Roux, unpublished results; [28]) a length difference of 14 AAs between the 2 *FRI* variants detected in the self-incompatible species *Arabidopsis lyrata* only conferred an 8-day difference in flowering time when these variants were transformed into *A. thaliana* [39]. Additional comparative analyses of flowering time genes in species with varying reproductive systems are needed to test the generality of this pattern.

### Advantages of combining association and traditional linkage mapping

The combination of linkage and association mapping clearly outperforms each method used in isolation. For example, QTL mapping increases our ability to distinguish true from false associations finely mapped by GWA mapping with a candidate gene enrichment of up to 7.4. This empirical result supports the notion that linkage and association mapping are complementary methods [20], [40]. Another advantage of a dual mapping strategy, rarely highlighted in the literature of GWA mapping, is related to the occurrence of false negatives. Controlling for population structure is necessary for reducing the false positive rate, but this approach also introduces false negatives. This may be a problem that is especially great for quantitative traits such as flowering time, the variation of which overlaps with population structure. While GWA mapping analyses on a less structured sample of natural accessions may solve this problem, it may also prevent detection of the genetic basis of natural variation occurring at the scale of the species. In contrast, our study shows that the use of QTL mapping in combination with GWA mapping on a worldwide sample of natural accessions may be an excellent alternative for detecting false negatives. In this study, 40% of the detected candidate genes overlapped by QTLs could be considered to be false negatives when analyzed by EMMA. We hypothesized that such a large fraction of false negatives could also occur for traits with a strong correlation between genetic relatedness and phenotypic similarity, for example, latitude related phenotypic traits like cold tolerance or relative growth rate [41]. A potential benefit of dual linkage and association mapping would be narrowing QTL intervals in RIL families. This might be achieved by using recently developed plant material such as advanced intercross RILs (AI RILs) [42] or multiple advanced generation inter-cross (MAGIC) lines, which would allow QTL intervals to be narrowed down to 300 kb [43]. On the other hand, the ongoing 216,509 SNP genotyping of the 14 parental lines will soon enable the nested association mapping (NAM) strategy to be undertaken [44]. Projecting the 216,509 SNP genetic information from parental lines onto the 4366 RILs included in this study will provide a powerful genetic resource for the scientific community. The joint analysis of data sets from the natural accessions and the 4366 RILs should greatly increase our power to finely map genomic regions associated with phenotypic variation.

Although flowering time is an easily scored quantitative trait, we must acknowledge that many other phenotypic traits will be challenging to score on 4,366 genetic lines. Two alternatives to phenotype a smaller number of lines while keeping enough statistical power to detect significant phenotype-genotype associations might be considered. First, we propose the use of the core-collections designed for every RIL family used in this study. Each core-collection contains 164 RILs and maximizes the genetic diversity and recombination observed within the RIL family of interest [23]. Second, the use of only 459 MAGIC lines might still provide a high enough resolution to fine map candidate genes within roughly 300kb [43].

### Identifying genes associated with flowering time natural variation

A striking, but not unexpected, result is the limited overlap between the genomic regions detected in our field experiment and the genes detected under greenhouse conditions. Large QTL×environment interactions for flowering time have already been demonstrated with RILs grown either in (natural or simulated) outdoor conditions versus controlled conditions [11], [12]. Only the *ETC3* and *ATGA20X7* genes supported by QTL regions in this study have also been proposed as candidate genes for flowering time phenotypes in GWA mapping studies scored under greenhouse conditions [13]. It is important to note that different phenotypic traits (number of leaves, bolting date) have been used as proxies for flowering time in these other studies; although these traits are generally highly correlated, the virtual absence of overlap between genes detected among experiments might result, at least in part, from the identity of the phenotypic traits scored as flowering time. For example, both bolting date and flowering date were scored in this study for the 2008/2009 experiment. While these traits are highly correlated, GWA mapping analyses indicate specific genetic bases for the time between bolting and flowering (our unpublished results). Taken together, these results confirm a strong role of genotype by environment interactions for flowering time in *A. thaliana* and reinforce the idea that varying environmental signals may reveal new genetic loci associated with flowering time variation.

Many candidate genes identified in this study are related to the photoperiod pathway and, more specifically, to circadian clock-related genes (*TOC1*, *CDF3*, *COL1*, *SRR1*, *AT5G59570*, and *LKP2*). The enrichment in circadian clock-related genes is in agreement with the experimental demonstration in *A. thaliana* of a fitness advantage to plants with a clock period that matched the environment [45], [46]. A significant enrichment of differentially expressed circadian clock-related genes has been found between early- and late-flowering *Capsella bursa-pastoris* [47], a co-occurring species and close sister group of *A. thaliana*, hence suggesting a parallel evolution of similar regulatory differences. Compared to stable greenhouse conditions, the difference in the candidate genes identified in natural conditions might originate from the plants experiencing new and/or less predictable environmental signals in outdoor conditions, such as decreasing and increasing photoperiod length or day-to-day fluctuation in temperature or light quality. For example, *TOC1*, part of the central oscillator of the *Arabidopsis thaliana* circadian clock [48], is entrained by photoperiod and thermocycles [49] and this drives rhythmic outputs, including seasonal control of flowering. For the light input to the clock, *LKP2* has been proposed as a candidate to serve as circadian photoreceptor [49], and *SRR1* activity is required for normal oscillator function *via* phytochrome-B-mediated light signaling [50]. Recently, *COL1* was found to be regulated by the circadian clock at warm temperatures [51]. Promoters of *COL1* contains motifs required for cold induction and might be thus proposed as a thermoreceptor. *LKP2* recognized *COL1* with an LOV domain [52] suggesting that the light and temperature signaling pathways might interact with each other.

Natural polymorphisms altering flowering time have been functionally validated in greenhouse studies for several flowering time genes: *CRY2*, *FRI*, *FLC*, *FLM*, *HUA2*, *PHYA*, *PHYB*, *PHYC*, and *PHYD* [53]. With the exception of *PHYD*, none of these candidates was confirmed by GWA mapping in our field study. A polymorphism in *PHYD* was previously found to be associated with bolting time in both natural accessions and the MAGIC lines [10]. Flowers *et al.* [30] hypothesized that an excess of replacement polymorphisms in *PHYD* may reflect either a recent relaxation of the selective constraints on this gene or adaptation to some environments. The detection of *PHYD* by GWA-mapping tends to support the second hypothesis. Two explanations might explain the absence of associations of the other functionally validated genes in our genome scan. First, the causative mutation may be rare, thus decreasing the power to detect an association through GWA. This might be the case for the *HUA2* loss-of-function allele, found only in a subset of lines [54], and *CRY2* and *FLM* with accession-specific mutations [55], [56]. *CRY2* is a particularly useful illustration of the ‘rare allele’ effect; a CRY2 point mutation conferring early flowering to plants grown in short photoperiods has only been detected in Cvi-0 in a worldwide survey [55], yet its importance is evident by a strong QTL in the Cvi-0×Col-0 RIL family in this study (bottom of chromosome 1). Second, the allelic effect might be sensitive to the environment, being determined by the timing of germination as demonstrated for *FRI* loss-of-function alleles [22]. In this example, a shift in germination date of a few days in early autumn (from mid- to late-September) cancelled the early-flowering phenotype associated with non-functional *FRI* alleles. Our failure to detect *FRI* in our study is consistent with the sowing period being performed in late September. Although this period coincides with natural germination flushes in *A. thaliana* populations in the North of France, it might be worth testing different germination dates.

GWA mapping has been shown to be powerful for detecting candidate genes associated with a quantitative trait [13]. On the other hand, GWA mapping should also be powerful at identifying genes that have not previously been described as candidates. In this study, 9 association peaks far from candidate genes and supported by QTL mapping and/or NILs were found to be associated with natural variation in flowering time. One of these association peaks has also been found by GWA mapping for different flowering time traits scored in the greenhouse [13]. *DOG1* (*DELAY OF GERMINATION 1*), first described in seed dormancy processes [57], is close to this association peak and has been proposed as a new candidate gene for flowering time [13]. However, *SAG12* (*SENESCENCE-ASSOCIATED GENE 12*), located 20 kb away from *DOG1* and closer to the association peak, might be a better candidate flowering gene. Indeed, *SAG12* accelerates rosette leaf senescence [58] and might induce early-flowering.

Functional validation of candidate genes found in this field experiment will certainly help complete our knowledge of the flowering time genetic network, as well as of the ecological and evolutionary significance of the genetic bases underlying flowering time natural variation. However we must keep in mind that functionally validating QTLs explaining less than 10%, as was the case for most of the QTLs detected in this study, might require appropriate genetic material like the Cre-lox transgenic lines developed for estimating a 9% fitness cost associated with the resistance gene *RPM1* [59].

## Materials and Methods

In this study, we combined genome-wide association (GWA) mapping based on 197 natural accessions genotyped for approximately 216,509 SNP markers (see section “Genome-Wide Association mapping”) with QTL mapping based on 13 RIL families for a total number of 4366 RILs. We also included 81 pairs of near-isogenic lines, and the parents of the RIL families. In total, 19,884 plants were phenotyped in a 2-year field experiment.

### Plant material

Four different groups of plant material were used in this study:

#### Natural accessions

A worldwide set of 197 natural accessions were phenotyped for flowering time in order to perform GWA mapping. All the accessions are listed in Table 1 in Dataset S1 and have been described elsewhere [60]. To reduce maternal effects prior to phenotyping, natural accessions were grown for one generation during 2007 under controlled greenhouse conditions (16 h photoperiod, 20°C) at the University of Lille 1 and their seeds collected. Of these, 184 accessions were genotyped for 216,509 SNPs evenly spaced across the genome [61]. The design of the Affymetrix genotyping array and genotyping protocols have been detailed elsewhere [13].

#### RILs and parental lines

Thirteen RIL families, including a total of 4,366 RILs (mean per family ∼336, range: 223–456) were obtained from the Centre of Ressources Biologiques (CRB, INRA Versailles; Table 11 in Dataset S1). The creation of the 13 RIL families was based on 14 natural accessions (*i.e.* 14 parental lines, namely Bay-0, Bla-1, Blh-1, Bur-0, Can-0, Col-0, Ct-1, Cvi-0, Ge-0, Nok-1, Ri-0, Shahdara, Tsu-0 and Yo-0) issued from a core collection designed to maximize both the genetic and phenotypic diversity in *A. thaliana* [21]. All but one RIL family (Bay-0×Shahdara) share the same recurrent parent Col-0 (Table 1). RILs resulted from 2 generations of intercrosses, followed by 5–6 generations of single seed descent. RIL families were genotyped for ∼82 genetic markers (range: 69–90; Table 11 in Dataset S1). To reduce maternal effects, the seeds were produced in the same controlled environment [23]. Further details on the creation of the 13 RIL families are available at the following website, http://dbsgap.versailles.inra.fr/vnat/.

#### NILs

Major additive QTLs found in the Bay-0×Shahdara RIL family were confirmed in NILs, following the heterogeneous inbred families (HIFs) strategy [62]. All 81 HIFs used in this study were developed from individual F_7_ RILs of the Bay-0×Shahdara cross to segregate in a single and limited genomic region [63]. For each RIL, several seeds were sown and genotyped individually for markers across the segregating region (Table 5 in Dataset S1). One to three independent fixed plants for each allele (*i.e.* NILs; F_8_) were chosen and allowed to self-fertilize. Depending on the RIL, F_9_ seeds from one or two independent plants fixed for each allele were then phenotyped to identify the phenotypic effect of Bay-0 *vs.* Shahdara alleles in the segregating region. Every genomic region is covered by at least 2 independent HIFs. NIL seeds distributed by the CRB were produced in the same controlled environment as the RIL seeds.

### Experimental design and growth conditions

#### 2007–2008 field experiment

For the first year of the field experiment, a total of 18,696 plants were phenotyped for flowering time. The experiment was organized in three blocks, each block being an independent randomization of 1 replicate per RIL, 2 replicates per natural accession, 4 replicates per NIL and 7 replicates per parental line of the RIL families. Each block, thus, included 6232 plants.

Seeds were sown half a block per day between from 24–29 September 2007 on damp standard culture soil (Huminsubstrat N3, Neuhaus) in arrays of 66 individual wells (Ø4 cm, vol. ∼38 cm^3^) (TEKU, JP 3050/66). This period coincides with natural germination flushes in *A. thaliana* populations in the North of France (personal observation, F. Roux). The experiment included 95 arrays per block for a total of 285 arrays. Two control accessions, Bg-2 (early flowering) and Lov-5 (late flowering) were placed in the same positions within each array in order to correct for micro-environmental variation. Seeds were stratified for 4 days at 4°C in the dark, in order to promote germination, and 3 seeds were placed in each well. In each block, the remaining 38 wells were left empty.

After the 4-days cold treatment, plants were placed in the greenhouse to protect seeds from rainfall. The conditions mimicked the outdoor conditions (no additional light or heating). While in the greenhouse, the arrays were rotated every day in order to reduce micro-environmental variation: half a block was moved from one end of the greenhouse to the other. In this way, all the plants experienced the same set of conditions during their early development. Plants were watered daily and no fertilizer was added.

Germination was monitored daily and the number of seedlings in each well scored. Most of the germination occurred 3–5 days after the cold treatment. The germination date considered in all analyses is the date of the first germination that occurred in a well.

Seedlings were thinned to one per well (17 days after the cold treatment) when most of the seedlings had reached the 4-leaf stage. On the day they were thinned, arrays were placed outside in a common garden located at the University of Lille 1, France. The 3 blocks (each of 4×24 arrays stuck some on the others) were arranged at 2-m spacing in the common garden. The density of plants was 576 plants/m^2^. This density corresponds to natural densities that seeds may experience when they are dispersed far from the maternal plant [64]. Soil had been tilled so that arrays could be slightly buried. Because the bottom of the wells was pierced, roots were able to reach the soil easily. The plants were watered for a week to ease the acclimatizing to outdoor conditions. Vertebrate herbivores were excluded by two successive fences. Molluscicide (PhytorexJ, Bayer Jardin) was added around experimental blocks to prevent slug attacks. Insects (mainly *Myzus persicae*) were biologically controlled by planting *Vicia faba* and *Tagetes patula* seedlings among the experimental blocks.

Plants were monitored every 3 days (1 block per day) or when non-freezing temperatures allowed it. Flowering date was scored as the number of days between germination and the appearance of the first open flower. Most studies aiming at studying natural variation of flowering time in *A. thaliana* scored bolting date (differentiation of the inflorescence from the apical meristem) or rosette leaf number as proxies for flowering time [3], [11], [22], [65]. While bolting date indicates the initiation of the reproductive structures, flowering date clearly indicates the beginning of offspring production.

#### 2008–2009 field experiment

Because climatic conditions vary annually at a specific geographical location, we re-grew a subset of plants in the following year to measure the reproducibility of flowering time scored in 2007–2008. For this second year of the field experiment, a total of 1188 plants were phenotyped for flowering time, including 6 replicates of 192 of the 197 accessions phenotyped in the 2007–2008 field experiment (see Table 1 in Dataset S1). The experimental design for the 2008–2009 experiment was exactly the same as for the 2007–2008 experiment. Seeds were sown on 24 September 2008, stratified for 4 days, and placed outside in the same common garden on 15 October 2008.

### Data analysis

We aimed to predict flowering time using temporal measures that incorporate fluctuating climatic conditions (see Text S1). In this study, flowering time was scaled in photothermal units (PTU) using a phenology model that integrates both photoperiod length and temperature [66]:
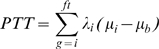
where PTT stands for “photothermal time” and is expressed in photothermal units (PTU, °C·daylight); *g* is the germination date; *ft* the flowering date; *i* spans the germination date to the flowering date, counting only the days with a mean temperature above 3°C; *μ_b_* is the optimal base temperature (3°C) for the developmental rate of the natural accession Col-0 [67]; *μ_i_* is the mean daily temperature during daylight and *λ_i_* is the daily photoperiod as a proportion of 24 h.

Accumulation of chilling degrees was calculated as follows:
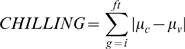
where CHILLING stands for the accumulation of chilling degrees across the winter; *μ_v_* is the threshold temperature below which vernalization occurs (*i.e.* 6°C, [22]); and *μ_c_* is the mean daily temperature, counting only days with a mean temperature below 6°C.

### Statistical analysis

Flowering time measured in units of PTT was analyzed with the general linear model (GLM) procedure in SAS9.1 (SAS Institute Inc., Cary, North Carolina, USA) according to the following model:
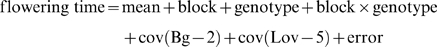
where ‘mean’ is the constant, ‘block’ accounts for differences in micro-environment among the 3 experimental blocks, ‘genotype’ measures the effect of genetic background (*i.e.* “natural accession”, “RIL”, “parental line” and “NIL”), ‘block×genotype’ was only considered for parental lines and NILs, cov(Bg-2) and cov(Lov-5) are covariates that represent trait values obtained for each array for the control accessions and accounts for array effects within block, and ‘error’ is the residual term. For NILs, data obtained from each of the 2 independent fixed plants for each allele are represented by a ‘family’ factor nested within ‘genotype’. All factors were treated as fixed effects because levels of no factor were random samples from a population to which we intended to extrapolate.

Transforming data did not improve normality in the “natural accessions” data set, and did so in only a few RIL families. To be consistent across analyses of all plant groups, we chose not to transform any data sets. Least-square means were obtained for each “genotype” and were subsequently used for GWA and QTL mapping analyses. Broad sense heritabilities (*H* ^2^) were estimated for the 13 RIL families from the mean square (MS) of GLM using the formula adapted from [68]. Phenotypic transgression was considered significant when the lowest or highest genetic value observed among RILs was more extreme than that observed for a parent, plus or minus twice the standard deviations [28], [69]. We estimated the intensity of phenotypic transgression for each RIL family by dividing the range of PTU values observed among RILs by the absolute difference of PTU between the corresponding parental lines. Kinship coefficients based on 189 informative SNPs [70] were used to calculate the genetic relatedness among the parental lines.

### Genome-wide association mapping

GWA analyses were based on a subset of the 197 natural accessions included in the experiment (184 for the 2007–2008 experiment). Two different analyses were performed [13]. In the first analysis, a Wilcoxon rank-sum test was run to test the association between phenotypes and genotypes for each marker. The second analysis, EMMA [24], is based on a mixed model that includes a matrix of genotype similarity among the accessions to control for population structure [13]. EMMA uses the following mixed model:
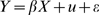
where Y is the vector of flowering time, X the vector of flowering time, *β* is the fixed phenotypic effect for the locus tested, and *u*∼N*_n_*(0, 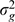 *K*) and *ε*∼N*_n_*(0, 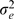 *I*) are random effects meant to capture the variance due to background genetics and environment, respectively.

### QTL mapping

QTL mapping analyses were performed independently for each of the 13 RIL families. The large size of each RIL family allowed us to simultaneously detect additive and epistatic QTLs using the QTLNetwork-2.0 software based on a mixed-model composite interval mapping (MCIM) method [71], [72]. Epistatic QTLs with or without single-locus effects were mapped. One- and two-dimensional genome scans for QTLs were performed using a 10 cM testing window, a 0.1 cM walk speed and a 0.5 cM filtration window size. To control the experimental type I error rate, a critical *F* value using the Satterthwaite method was estimated by performing a permutation test with at least 1000 permutations of the original data for each RIL family. QTL effects and QTL confidence intervals were estimated with a Bayesian method *via* the summary of the Gibbs samplers (Gibbs sample size = 20,000).

In order to compare the genetic architecture of flowering time between 2 environments, the MCIM method using the QTLNetwork-2.0 software was also applied on flowering time data scored under greenhouse conditions for 5 RIL families common to this study [23].

### Enrichment for *a priori* candidate genes

An *a priori* list of 266 candidate genes was primarily retrieved from Atwell *et al.* [13] (Table 12 in Dataset S1) who searched TAIR8 (The Arabidopsis Information Resource, at http://arabidopsis.org/) for genes with annotations related to flowering time. In addition, a few genes were added following our literature searches, resulting in a list of 282 candidate genes in total.

When considering GWA mapping results, we focused on the SNPs presenting the highest association (top SNPs) with flowering time for each GWA mapping analysis (EMMA and Wilcoxon, for the 2007–2008 field experiment). We checked whether those top SNPs were located within the confidence interval of a QTL and whether they were located within 20 kb of one of the 282 candidate genes. The 20 kb window is conservative given that linkage disequilibrium in *A. thaliana* decays per 10 kb on average [61].

As described in Atwell *et al.* [13], enrichment was then calculated for SNPs within QTL confidence intervals and/or within 20Kb of an *a priori* candidate gene.

To determine the threshold number of top SNPs above which additional top SNPs would behave like the rest of the genome, enrichment was calculated for progressively more selective sets of top SNPs (3000, 2000, 1000, 500, 400, 300, 200, 100, 50). For each set of top SNPs, a null distribution of enrichment was generated to determine a 95% confidence interval (see Text S2 for description of the algorithm).

### Genotyping candidate genes

Common polymorphisms previously found to be associated with flowering time were genotyped for the 197 natural accessions and the 14 parental lines. Functionality of the *FRIGIDA* allele was determined according to sequencing data [60]. *FLOWERING LOCUS C* functionality was determined according to Caicedo *et al.* [26]. Weak *FLC* alleles were determined by genotyping either the Ler miniature inverted repeat transposable element (MITE) 1224 bp insertion or the Da (1)-12 transposable element 4009 bp insertion. Shahdara and C24 accessions have also been reported to contain a functional *FRI* allele and a weak or nonfunctional *FLC* allele [73]. The two major *FLC* haplogroups (*FLCA* and *FLCB*) previously suggested to be associated with flowering time variation in *A. thaliana* under field conditions were determined by using the PCR primers described in Caicedo *et al.* [26]. A promoter indel upstream of the start codon of *PHYTOCHROME C* was genotyped according to Balasubramanian *et al.* [29].

## Supporting Information

Dataset S1 Supplementary tables.
(0.82 MB PDF)

Figure S1 A year-to-year comparison of flowering time expressed in Julian days (left panel) and photothermal units (right panel).
(0.06 MB TIF)

Figure S2 Distribution of flowering time expressed in photothermal units (PTU) for the 2007–2008 field experiment. Top: distribution of flowering time for the 197 natural accessions. Below: distribution of flowering time for each of the 13 RIL families. For each RIL family, red bars extend from the minimum to the maximum values observed, with the larger ticks demarcating the median of the distribution and the smaller ticks indicating the flowering times for the parental lines.
(0.14 MB TIF)

Figure S3 Relationship between flowering time and latitude. Accessions bearing non-functional and functional *FRI* alleles are depicted by empty and filled dots, respectively. Drawn lines correspond to regression lines for accessions with a functional *FRI* allele (dotted line), a non-functional *FRI* allele (dashed line), a non-functional *FRI* allele excluding Cvi-0 (i.e., latitude<20°C; dashed-dotted line).
(0.12 MB TIF)

Figure S4 Distribution of flowering time for each RIL family.
(0.69 MB TIF)

Figure S5 Network of additive and epistatic QTLs for flowering time for each RIL family.
(0.38 MB PDF)

Figure S6 Quantile-Quantile plot of p-values (raw and negative logarithm) in genome-wide scans for flowering time. The different curves correspond to different analyses of GWA mapping. Dashed black line: expected; dashed red line: Wilcoxon; solid red line: Wilcoxon with minor allele relative frequency (MARF)>0.1; dashed blue line: EMMA; solid blue line: EMMA with MARF>0.1.
(0.13 MB TIF)

Figure S7 The distribution dependence of p-value distributions on minor allele relative frequency (MARF) for EMMA (top panel) and Wilcoxon (bottom panel).
(0.51 MB TIF)

Figure S8 Comparison of GWA and traditional linkage mapping (additive QTLs) results for flowering time for chromosomes 1, 2, 3, and 5.
(1.94 MB PDF)

Figure S9 Comparison of GWA and traditional linkage mapping (epistatic QTLs) results for flowering time for chromosomes 1, 4, and 5. No epistatic QTLs were found on chromosomes 2 and 3.
(0.77 MB PDF)

Figure S10 Enrichment ratios as a function of the number of top SNPs chosen in the GWA mapping results using the Wilcoxon test. The mean and the corresponding 95% confidence interval from the null distributions are represented by the dotted line and the colored areas, respectively. CG: candidate gene.
(0.10 MB TIF)

Text S1 Photothermal time.
(0.05 MB PDF)

Text S2 Enrichment and null distribution computation.
(0.01 MB PDF)
