# Supplementary material for: Linkage and Association Mapping of Arabidopsis thaliana Flowering Time in Nature
Source: PLoS Genet. 2010 May 6;6(5):e1000940. doi: 10.1371/journal.pgen.1000940 (PMC2865524; doi:10.1371/journal.pgen.1000940)
Supplement: Dataset S1 — Supplementary tables. (0.82 MB PDF) [file pgen.1000940.s001.pdf]

Table 1. List of natural worldwide accessions and RIL parental lines used in this study.

Material	Name	latitude	longitude	2008-2009	216kSNPs	FRI	NF FRI allele	PHYC	FLC	FLC_A-FLC_B
NA	Ag-0	45	1.3	x	X	F		0	AA	A
NA	Alc-0	40.31	-3.22	x	X	NF		0	WA	A
NA	Algutsrum	56.68	16.5	x	X	F		1	AA	.
NA	An-1	51.22	4.4	x	X	NF	P	0	AA	A
NA	Ang-0	50.3	5.3	x	X	NF	S/T	0	AA	A
NA	Bå1-2	56.4	12.9	x	X	F		1	AA	B
NA	Bå3-3	56.4	12.9	x		F		1	AA	A
NA	Bå4-1	56.4	12.9	x	X	F		0	AA	A
NA	Bå5-1	56.4	12.9	x	X	F		0	AA	A
NA	Bay-0	49	11	x	X	NF	B	1	AA	B
NA	Bg-2	47.65	-122.31		X	NF	H	0	AA	A
NA	Bil-5	63.32	18.48	x	X	F		1	AA	A
NA	Bil-7	63.32	18.48	x	X	F		1	AA	A
NA	Bla-1	41.68	2.8	x	X	F		0	ND	A
NA	Blh-1	48	19	x		NF	L	0	AA	B
NA	Boo2-1	55.86	13.51	x	X	F		1	AA	A
NA	Bor-1	49.4	16.23	x	X	F		0	ND	A
NA	Bor-4	49.4	16.23	x	X	F		0	ND	A
NA	Br-0	49.2	16.62	x	X	F		-	AA	A
NA	Brö1-6	56.3	16	x	X	F		1	AA	A
NA	Bs-1	47.5	7.5	x	X	NF	B	0	AA	B
NA	Bu-0	50.5	9.5	x	X	NF	L	0	AA	A
NA	Buckhorn Pass	41.36	-122.76	x	X			0	AA	A
NA	Bur-0	54.1	-6.2	x	X	F		0	AA	A
NA	C24	40.21	-8.43	x	X	F		0	WA	A
NA	Can-0	29.21	-13.48	x	X	F		0	AA	A
NA	Cen-0	49	0.5	x	X	F		1	AA	A
NA	CIBC-17	51.41	-0.64	x	X	F		0	AA	A
NA	CIBC-5	51.41	-0.64	x	X	F		0	AA	A
NA	Co			x	X	NF	R	0	AA	A
NA	Col-0	38.3	-92.3	x	X	NF	L	1	AA	A
NA	CS22491	61.36	34.15	x	x	F		0	ND	B
NA	Ct-1	37.3	15	x	X	NF	B	0	AA	B
NA	Cvi-0	15.11	-23.62	x	X	NF	I	0	AA	A
NA	Dem-4	41.19	-87.19	x	X	F		0	AA	A
NA	Dra-3-1	55.76	14.12	x	X	F		1	AA	A
NA	DraII-1	49.41	16.28	x	X	F		0	AA	A
NA	DraIII-1	49.41	16.28	x	X	F		1	WA	A
NA	Duk	49.1	16.2	x	X	NF	B	1	WA	A
NA	Eden-1	62.88	18.18		X	F		0	AA	A
NA	Eden-2	62.88	18.18	x	X	F		0	AA	A
NA	Edi-0	56	-3	x	X	F		0	AA	B
NA	Eds-1	62.9	18.4	x	X	F		1	AA	A
NA	Ei-2	50.3	6.3	x	X	F		0	AA	A
NA	En-1	50	8.5	x	X	NF	B	0	AA	B
NA	Est-1	58.3	25.3	x	X	NF	L	0	AA	B
NA	Fab-2	63.02	18.32	x	X	F		0	AA	A
NA	Fab-4	63.02	18.32		X	F		0	AA	A
NA	Fei-0	40.5	-8.32	x	X	NF	O	0	AA	A
NA	Fjä1-1	56.06	14.29	x	X			0	AA	A
NA	Ga-0	50.3	8	x	X	NF	B	1	AA	A
NA	Gd-1	53.5	10.5	x	X	NF	L	0	AA	B
NA	Ge-0	46.5	6.08	x	X	F		1	AA	A
NA	Got-22	51.53	9.94	x	X	F		0	AA	B
NA	Got-7	51.53	9.94	x	X	F		0	AA	B
NA	Gr-1	47	15.5	x	X	NF	B	0	ND	A
NA	Gu-0	50.3	8	x	X	NF	L	1	AA	B
NA	Gul1-2	56.3	16	x		F		0	AA	A

Table 1. (continued)

Material	Name	latitude	longitude	2008-2009	216kSNPs	FRI	NF FRI allele	PHYC	FLC	FLC_A-FLC_B
NA	Gy-0	49	2	x	x	NF	P	0	AA	A
NA	H55	49	15	x	x	NF	L	1	AA	A
NA	Hi-0	52	5	x	x	NF	B	-	AA	B
NA	Hod	48.8	17.1	x	x	F		1	AA	B
NA	Hov2-1	56.1	13.74	x	x			0	ND	A
NA	Hov4-1	56.1	13.74	x	x	F		1	AA	A
NA	Hovdala-2	56.1	13.74	x	x	F		1	AA	B
NA	HR-10	51.41	-0.64	x	x	NF	K	-	AA	A
NA	HR-5	51.41	-0.64	x	x	NF	K	0	AA	A
NA	Hs-0	52.24	9.44	x	x	NF	L	0	AA	B
NA	HSm	49.33	15.76	x	x	F		0	WA	.
NA	In-0	47.5	11.5	x	x	F		0	AA	B
NA	Is-0	50.5	7.5	x	x	NF	B	0	AA	B
NA	Jm-0	49	15	x	x	NF	B	0	AA	B
NA	Ka-0	47	14	x	x	NF	B	1	AA	A
NA	Kas-1	35	77	x		F		0	AA	B
NA	Kent	51.15	0.4	x	x	NF	B	1	AA	B
NA	Kin-0	44.46	-85.37	x	x	NF	K	0	AA	A
NA	Kni-1	55.66	13.4	x	x	F		0	AA	B
NA	Kno-10	41.28	-86.62	x	x	F		0	AA	A
NA	Kno-18	41.28	-86.62	x	x	F		0	AA	A
NA	Köln	51	7	x	x	NF	L	0	AA	B
NA	Kondara	38.48	68.49	x	x	F		-	AA	B
NA	Kulturen-1	55.71	13.2	x	x			1	AA	A
NA	Kz-1	49.5	73.1	x	x	F		-	AA	B
NA	Kz-13	49.5	73.1	x		F		1	WA	B
NA	Kz-9	49.5	73.1	x	x	F		1	ND	B
NA	Lc-0	57	-4	x	x	NF	O	1	AA	B
NA	Ler-1	47.98	10.87	x	x	NF	B	0	WA	A
NA	Liarum	55.95	13.85	x	x	F		1	AA	A
NA	Lillö-1	56.15	15.78	x	x	F		1	AA	A
NA	Lip-0	50	19.3	x	x	F		1	AA	A
NA	Lis-1	56	14.7	x	x	F		1	WA	B
NA	Lis-2	56	14.7	x	x	F		1	AA	A
NA	Lisse	52.25	4.57	x	x			0	AA	.
NA	LL-0	41.59	2.49	x	x	F		0	ND	A
NA	Lm-2	48	0.5	x	x	NF	B	0	AA	A
NA	Lom1-1	56.09	13.9	x	x	NF	B	0	AA	B
NA	Lov-1	62.8	18.08	x	x	F		0	ND	A
NA	Lov-5	62.8	18.08		x	F		0	ND	.
NA	Lp2-2	49.38	16.81	x	x	F		0	WA	A
NA	Lp2-6	49.38	16.81	x	x	F		0	WA	A
NA	Lu-1	55.71	13.2	x	x	F		1	WA	A
NA	Lund	55.71	13.2	x	x	F		0	AA	B
NA	Lz-0	46	3.3	x	x	NF	B	0	AA	A
NA	Mir-0	44	12.37	x	x	F		0	AA	A
NA	Mr-0	44.15	9.65	x	x	F		0	AA	A
NA	Mrk-0	49	9.3	x	x	NF	B	-	AA	B
NA	Ms-0	55.75	37.63	x	x			1	AA	B
NA	Mt-0	32.34	22.46	x	x	NF	L	1	AA	A
NA	Mz-0	50.3	8.3	x	x	NF	L	0	AA	B
NA	N6002	43.77	11.25	x	x	F		0	AA	A
NA	N6034	48.61	2.31	x		F		0	AA	A
NA	N6041	50.07	8.53	x	x			0	AA	B
NA	N6187	47	-122.2	x	x	F		0	AA	A
NA	Na-1	47.5	1.5	x	x	NF	B	0	AA	B
NA	NC-6	35	-79.18	x		F		0	AA	A
NA	Nd-1	50	10	x	x	NF	L	1	AA	A
NA	NFA-10	51.41	-0.64	x	x	NF	S/T	-	AA	A

Table 1. (continued)

Material	Name	latitude	longitude	2008-2009	216kSNPs	FRI	NF FRI allele	PHYC	FLC	FLC_A-FLC_B
NA	NFA-8	51.41	-0.64	x	x	NF	K	0	AA	A
NA	Nok-3	52.24	4.45	x	x	F		0	AA	B
NA	Nw-0	50.5	8.5	x	x	NF	L	-	AA	B
NA	Nyl-2	62.96	18.28	x	x	F		1	AA	A
NA	Omo-2-1	56.14	15.78	x	x	F		1	AA	A
NA	Omo-2-3	56.14	15.78	x	x	F		1	AA	B
NA	Ör-1	56.45	16.11	x	x	F		1	AA	A
NA	Ost-0	60.25	18.37	x	x	F		1	AA	A
NA	Oy-0	60.23	6.13	x	x	NF	L	0	AA	A
NA	Pa-1	38.07	13.22	x	x	F		0	AA	A
NA	Per-1	58	56.32	x	x	F		1	WA	A
NA	Petergof	59	29	x	x	NF	B	1	AA	B
NA	Pi-0	47.04	10.51	x		F		0	AA	B
NA	Pla-0	41.5	2.25	x	x	F		0	WA	A
NA	Pna-10	42.09	-86.33	x	x	F		0	AA	A
NA	Pna-17	42.09	-86.33	x	x	NF	Q	0	AA	B
NA	Pro-0	43.25	-6	x	x	NF	N	0	AA	A
NA	Pu2-23	49.42	16.36	x	x	F		0	ND	A
NA	Pu2-7	49.42	16.36	x	x	F		0	ND	A
NA	Pu2-8	49.42	16.36	x	x	F		0	WA	A
NA	Ra-0	46	3.3	x	x	NF	B	0	AA	A
NA	Rak-2	49	16	x	x	NF	B	0	AA	B
NA	Rd-0 CS1482	50.5	8.5	x	x	NF	L	0	AA	B
NA	Rd-0 CS1483	50.5	8.5	x	x	NF	L	0	AA	B
NA	Ren-1	48.5	-1.41	x	x	NF		0	AA	A
NA	Ren-11	48.5	-1.41	x	x	NF	F	0	AA	A
NA	Rev-1	55.7	13.4	x	x	F		0	AA	B
NA	Rmx-A02	42.04	-86.51	x	x	F		0	AA	.
NA	Rmx-A180	42.04	-86.51	x	x	NF	O	0	ND	.
NA	RRS-10	41.56	-86.43	x	x	F		0	AA	A
NA	RRS-7	41.56	-86.43	x	x	NF	B	0	AA	A
NA	Rsch-4	56.3	34	x	x	NF	B	1	AA	B
NA	Rubezhnoe-1	49	38.28	x	x	F		1	AA	B
NA	San-2	56.07	13.74	x	x	F		1	AA	B
NA	Sanna-2	62.69	18	x	x	NF	J	0	WA	A
NA	Santa Clara	37.21	-121.16	x		NF	B	1	AA	A
NA	Sap-0	49.49	14.24	x	x	F		1	WA	A
NA	Sav-0	49.18	15.88	x	x			1	WA	A
NA	Se-0	38.33	-3.53	x	x	F		0	ND	A
NA	Sf-1	41.78	3.03	x		F		0	WA	A
NA	Shahdara	38.35	68.48	x	x	F		-	WA	B
NA	Sorbo	38.35	68.48	x	x	F		-	AA	B
NA	Spr-1-2	56.3	16	x	x	F		1	AA	A
NA	Spr-1-6	56.3	16	x	x	F		1	AA	A
NA	Sq-1	51.41	-0.64	x	x	NF	S/T	0	AA	A
NA	Sq-8	51.41	-0.64	x	x	NF	L	0	AA	B
NA	Sr:5	58.9	11.2	x	x	F		0	WA	A
NA	St-0	59	18	x	x	F		0	AA	A
NA	Stu1-1	56.4	16.11	x		F		1	AA	A
NA	Stw-0	52	36	x	x	F		1	WA	A
NA	Ta-0	49.5	14.5	x	x	F		1	WA	A
NA	Tamm-2	60	23.5	x	x	F		1	AA	A
NA	Tamm-27	60	23.5	x	x	F		1	AA	A
NA	Tottarp-2	55.95	13.85	x	x	F		1	AA	A
NA	Ts-1	41.72	2.93	x	x	F		0	ND	A
NA	Ts-5	41.72	2.93	x	x	F		0	AA	A
NA	Tsu-1	34.43	136.31	x	x	NF	B	-	AA	B
NA	Tu-0	45	7.5	x	x	NF	B	-	AA	B
NA	Ull-2-3	56.06	13.97	x	x	NF	M	0	ND	.

Table 1. (continued)

Material	Name	latitude	longitude	2008-2009	216kSNPs	FRI	NF FRI allele	PHYC	FLC	FLC_A-FLC_B
NA	Ull-2-5	56.06	13.97	x	x	F		-	AA	A
NA	Ull1-1	56.06	13.97	x	x			1	AA	B
NA	Uod-1	48.3	14.45	x	x	NF	B	0	AA	A
NA	Uod-2	48.3	14.45	x				1	AA	A
NA	Uod-7	48.3	14.45	x	x	F		0	AA	A
NA	Van-0	49.3	-123	x	x	F		1	AA	A
NA	Var-2-1	55.58	14.33	x	x	F		-	AA	A
NA	Var-2-6	55.58	14.33	x	x	F		-	AA	A
NA	Västervik	57.75	16.63	x	x			-	AA	A
NA	Vimmerby	57.7	15.8	x	x	F		-	AA	B
NA	Vinslöv	56.1	13.92	x	x			0	AA	B
NA	Wa-1	52.3	21	x	x	F		1	AA	B
NA	Wei-0	47.25	8.26	x	x	NF	B	0	AA	A
NA	Wil-1	54.68	25.32	x		F		1	AA	A
NA	Wil-1 Dean	54.68	25.32		x	F		1	AA	A
NA	Ws-0	52.3	30	x	x	F		1	AA	A
NA	Ws-2	52.3	30	x	x	F	L	1	AA	B
NA	Wt-5	52.3	9.3	x	x	NF	B	0	AA	B
NA	Yo-0	37.45	-119.35	x	x	F		0	AA	A
NA	Zdr-1	49.39	16.25	x	x	NF	B	1	ND	A
NA	Zdr-6	49.39	16.25	x	x	F		1	WA	A
Parent	Bay-0	49	11		x	NF	B	1	AA	B
Parent	Bla-1	41.68	2.8		x	F		0	AA	A
Parent	Blh-1	48	19		x	F		1	AA	A
Parent	Bur-0	54.1	-6.2		x	F		0	AA	A
Parent	Can-0	29.21	-13.48		x	F		0	AA	A
Parent	Col-0	38.3	-92.3		x	NF	L	1	AA	A
Parent	Ct-1	37.3	15		x	NF	B	0	AA	B
Parent	Cvi-0	15.11	-23.62		x	NF	I	0	AA	A
Parent	Ge-0	46.5	6.08		x	F		1	AA	A
Parent	Nok-1	52.24	4.45		x	F		0	AA	B
Parent	Ri-0	49.1	-123		x	NF		0	AA	A
Parent	Shahdara	38.35	68.48		x	F		-	WA	B
Parent	Tsu-0	34.43	136.31		x	NF	B	-	AA	B
Parent	Yo-0	37.45	-119.35		x	F		0	AA	A

Plant material: NA = natural worldwide accession, Parent = parental lines of the 13 RIL families.

2008-2009: natural accessions phenotyped during the 2008-2009 field experiment.

216kSNPs: natural accessions genotyped for 216,509 SNP markers (Atwell *et al.* 2010).

FRI: functionality of the *FRIGIDA* gene according to Shindo *et al.* (2006). NF = non-functional, F = functional.

NF FRI allele: loss-of-function alleles (Shindo *et al.* 2006).

PHYC: promoter indel upstream of the start codon of PHYTOCHROME C (Balasubramanian *et al.* 2006). 0, Ler haplotype; 1, Col haplotype.

FLC: functionality of the *FLC* gene. AA = active allele, WA = weak or non-functional allele, ND = not defined (*i.e.* the presence/absence of transposon insertions could not be determined).

FLC_A-FLC_B: major *FLC* haplogroups (Caicedo *et al.* 2004).

Table 2. Effect of *Arabidopsis thaliana* plant genotype on flowering time scored in the field experiment: natural accessions, parental lines and RILs.

group	family	cross	Source	Type III SS	df	MS	F-ratio	*P*
Group 1: Natural Accessions			Block	1.56E+04	2	7812.60	22.53	0.0000
			Genotype	2.35E+06	196	12008.63	34.64	0.0000
			Cov(Lov5)	1.41E+03	1	1405.19	4.05	0.0444
			Cov(Bg2)	1.73E+04	1	17271.86	49.82	0.0000
			Error	2.94E+05	848	346.70		
Group 3: Parental lines			Block	4.25E+03	2	2126.258	10.325	0.0000
			Genotype	8.19E+05	13	62967.006	305.769	0.0000
			Block*Genotype	5.22E+02	1	522.282	2.536	0.113
			Cov(Lov5)	6.96E+03	1	6958.25	33.789	0.0000
			Cov(Bg2)	1.28E+04	26	492.984	2.394	0.0000
			Error	4.61E+04	224	205.93		
Groupe 2: RIL families	2RV	Col-0XBla-1	Block	1.35E+04	2	6738.38	8.52	0.0002
			Genotype	1.61E+06	239	6748.71	8.54	0.0000
			Cov(Lov5)	3.02E+03	1	3022.89	3.82	0.0514
			Cov(Bg2)	4.73E+03	1	4734.73	5.99	0.0149
			Error	2.66E+05	337	790.62		
	3RV	Col-0XNok-1	Block	1.50E+04	2	7516.28	11.24	0.0000
			Genotype	1.16E+06	273	4261.84	6.37	0.0000
			Cov(Lov5)	5.39E+00	1	5.39	0.01	0.9285
			Cov(Bg2)	2.02E+04	1	20174.05	30.18	0.0000
			Error	2.92E+05	437	668.54		
	4RV	Col-0XNok-1	Block	1.50E+04	2	7481.73	11.52	0.0000
			Genotype	6.95E+05	221	3145.66	4.84	0.0000
			Cov(Lov5)	2.86E+01	1	28.60	0.04	0.8339
			Cov(Bg2)	4.14E+03	1	4137.75	6.37	0.0120
			Error	2.45E+05	378	649.41		
	6RV	Col-0XRi-0	Block	3.71E+03	2	1855.37	2.95	0.0535
			Genotype	6.03E+05	283	2132.08	3.38	0.0000
			Cov(Lov5)	1.39E+04	1	13862.23	22.01	0.0000
			Cov(Bg2)	9.57E+03	1	9573.90	15.20	0.0001
			Error	3.19E+05	507	629.92		
	7RV	Col-0XCt-1	Block	2.21E+04	2	11067.16	18.40	0.0000
			Genotype	8.39E+05	375	2236.03	3.72	0.0000
			Cov(Lov5)	2.97E+03	1	2966.70	4.93	0.0267
			Cov(Bg2)	1.52E+04	1	15184.65	25.24	0.0000
			Error	3.96E+05	659	601.61		
	8RV	Col-0XCvi-0	Block	2.02E+04	2	10110.44	12.39	0.0000
			Genotype	4.80E+06	365	13152.60	16.12	0.0000
			Cov(Lov5)	4.39E+03	1	4386.39	5.37	0.0207
			Cov(Bg2)	1.06E+04	1	10551.64	12.93	0.0003
			Error	5.56E+05	681	816.08		
	13RV	Col-0XSha	Block	1.76E+04	2	8808.97	8.59	0.0002
			Genotype	2.39E+06	339	7052.95	6.88	0.0000
			Cov(Lov5)	3.48E+02	1	347.75	0.34	0.5605
			Cov(Bg2)	1.34E+04	1	13370.07	13.04	0.0003
			Error	5.90E+05	576	1024.99		
	17RV	Col-0XGe-0	Block	1.16E+04	2	5793.78	21.20	0.0000
			Genotype	3.82E+05	337	1133.10	4.15	0.0000
			Cov(Lov5)	2.03E+03	1	2031.31	7.43	0.0066
			Cov(Bg2)	1.94E+04	1	19427.93	71.09	0.0000
			Error	1.67E+05	611	273.29		

Table 2. (continued)

group	family	cross	Source	Type III SS	df	MS	F-ratio	*P*
Group 2: RIL families	19RV	Col-0XCan-0	Block	1.99E+04	2	9935.25	16.14	0.0000
			Genotype	1.62E+06	370	4377.71	7.11	0.0000
			Cov(Lov5)	1.39E+04	1	13943.94	22.66	0.0000
			Cov(Bg2)	9.97E+03	1	9969.18	16.20	0.0001
			Error	4.12E+05	670	615.40		
	20RV	Col-0XBur-0	Block	1.83E+04	2	9170.15	15.99	0.0000
			Genotype	1.27E+06	340	3749.11	6.54	0.0000
			Cov(Lov5)	3.13E+03	1	3130.65	5.46	0.0198
			Cov(Bg2)	1.00E+04	1	10037.27	17.51	0.0000
			Error	3.24E+05	565	573.33		
	21RV	Col-0XBlh-1	Block	1.10E+04	2	5499.05	6.92	0.0011
			Genotype	1.07E+06	310	3460.03	4.36	0.0000
			Cov(Lov5)	1.07E+04	1	10721.80	13.50	0.0003
			Cov(Bg2)	2.47E+04	1	24699.44	31.09	0.0000
			Error	4.30E+05	541	794.36		
	23RV	Col-0XYo-0	Block	3.18E+04	2	15921.56	38.39	0.0000
			Genotype	9.06E+05	455	1991.71	4.80	0.0000
			Cov(Lov5)	9.43E+03	1	9427.23	22.73	0.0000
			Cov(Bg2)	1.61E+04	1	16134.93	38.90	0.0000
			Error	3.46E+05	834	414.76		
	33RV	Bay-0XSha	Block	2.41E+04	2	12046.49	20.50	0.0000
			Genotype	1.33E+06	406	3267.72	5.56	0.0000
			Cov(Lov5)	3.55E+03	1	3548.45	6.04	0.0142
			Cov(Bg2)	9.55E+03	1	9549.23	16.25	0.0001
			Error	4.15E+05	706	587.54		

Table 3. Additive QTLs for flowering time scored in the field experiment.

RIL family	QTL	interval	position	range	A	SE	P-Value	h^2(a)
2RV	1-18	c1_28454-c1_29898	103.6	101.0-105.6	25.1383	2.3145	<0.0001	0.2447
	3-12	c3_19628-c3_20729	69.6	63.8-76.1	-11.0985	2.2734	<0.0001	0.0387
	4-1	c4_00042-c4_02133	1.3	0.0-5.6	-21.1668	2.2905	<0.0001	0.1307
	5-15	c5_24997-c5_26671	90.3	87.8-92.1	27.5738	2.3353	<0.0001	0.1855
3RV	1-12	c1_18368-c1_20384	55.1	45.6-61.1	10.168	1.7252	<0.0001	0.0282
	2-13	c2_17606-c2_18753	55.7	53.2-58.1	8.8091	1.6306	<0.0001	0.0945
	4-4	c4_04877-c4_05850	13.1	0.5-19.2	6.9853	1.711	<0.0001	0.0076
	5-3	c5_02900-c5_04011	11.7	10.5-13.2	32.0854	1.6761	<0.0001	0.455
4RV	2-8	c2_10250-c2_12435	39.2	34.9-46.2	11.8479	1.79	<0.0001	0.0939
	3-11	c3_15117-c3_17552	51.2	44.3-57.1	-7.574	1.6349	<0.0001	0.0351
	4-10	c4_11878-c4_13171	43.2	41.3-45.1	-18.7506	1.7151	<0.0001	0.3093
6RV	5-4	c5_04011-c5_05319	14.4	12.4-15.6	11.2515	1.4196	<0.0001	0.1267
	5-17	c5_24997-c5_26321	89	86.9-90.8	13.5894	1.4729	<0.0001	0.2026
7RV	3-13	c3_20729-c3_22147	71.5	66.6-74.1	4.9904	1.0901	<0.0001	0.0365
	4-6	c4_05850-c4_06923	20.7	14.3-25.1	4.3549	1.0927	<0.0001	0.035
	4-10	c4_08930-c4_10609	44.9	38.6-51.3	4.6542	1.1052	<0.0001	0.0025
	5-4	c5_04011-c5_05319	13.7	12.0-15.1	16.3352	1.0815	<0.0001	0.3104
	5-20	c5_24997-c5_26671	89.4	85.3-93.3	8.9199	1.1185	<0.0001	0.0781
8RV	1-1	c1_00593-c1_02212	1.5	0.3-2.7	48.0923	2.0737	<0.0001	0.4562
	1-23	c1_28667-c1_29898	135.8	130.2-137.9	13.0275	2.1161	<0.0001	0.0186
	4-12	c4_11878-c4_13171	57.9	52.7-61.5	13.1663	2.0577	<0.0001	0.0313
	5-3	c5_02900-c5_04011	17.3	14.0-22.5	-12.18	2.0308	<0.0001	0.0495
	5-7	c5_07442-c5_08563	33.1	30.5-35.4	-9.8079	2.049	<0.0001	0.0201
	5-18	c5_24997-c5_26671	111.1	108.3-112.7	18.4554	2.0853	<0.0001	0.077
13RV	1-20	c1_28454-c1_29898	93.2	89.9-96.2	13.0769	1.9835	<0.0001	0.0404
	2-7	c2_07650-c2_10250	27.1	22.8-31.4	-16.8388	2.105	<0.0001	0.0334
	2-14	c2_17606-c2_18753	61.2	58.3-61.2	14.3015	1.9134	<0.0001	0.1001
	3-6	c3_05141-c3_06631	8	1.4-11.0	13.299	1.9551	<0.0001	0.0473
	4-11	c4_11878-c4_13171	50.6	42.9-53.7	-12.6146	2.0365	<0.0001	0.0176
	5-3	c5_02900-c5_04011	10.3	8.1-11.9	21.9281	1.9517	<0.0001	0.145
	5-17	c5_24997-c5_26671	83	80.0-85.4	26.7251	2.0109	<0.0001	0.2064
17RV	2-12	c2_17606-c2_18753	62.6	59.7-64.4	5.075	0.8114	<0.0001	0.0669
	3-2	c3_01901-c3_02968	3.8	1.5-6.3	5.7749	0.7789	<0.0001	0.0766
	4-1	c4_00641-c4_02133	2	0.0-4.6	-7.603	0.8169	<0.0001	0.0691
	5-5	c5_05319-c5_06820	26.2	24.3-32.0	-9.0636	0.7996	<0.0001	0.1341
	5-18	c5_24997-c5_26321	94.8	92.7-94.8	8.6772	0.7703	<0.0001	0.1777
19RV	1-19	c1_26993-c1_28454	98.6	96.8-102.7	10.7099	1.2869	<0.0001	0.0957
	4-1	c4_00641-c4_02133	0.1	0.0-0.7	-13.8883	1.2662	<0.0001	0.0308
	4-10	c4_10609-c4_11878	27.4	25.6-31.1	21.6857	1.312	<0.0001	0.2464
	5-3	c5_02900-c5_04011	10.7	7.4-16.9	-4.1901	1.3035	<0.0001	0.0181
	5-17	c5_24997-c5_26671	90.9	89.2-92.7	19.4886	1.3855	<0.0001	0.2085
20RV	1-4	c1_04176-c1_05593	8.3	0.0-11.8	-6.3288	1.4724	<0.0001	0.0551
	1-10	c1_13869-c1_15634	41.9	40.3-45.9	-15.1421	1.4661	<0.0001	0.0816
	4-10	c4_10609-c4_11878	37.6	35.3-40.0	-13.2772	1.5125	<0.0001	0.0779
	5-4	c5_04011-c5_05319	10.7	9.6-14.9	21.8483	1.5027	<0.0001	0.268
	5-17	c5_22415-c5_23116	74.3	70.9-76.8	-10.7022	1.4991	<0.0001	0.0661

Table 3. (continued)

RIL family	QTL	interval	position	range	A	SE	P-Value	h^2(a)
21RV	3-13	c3_20729-c3_22147	67.5	61.1-70.1	5.9157	1.8354	<0.0001	0.0414
	4-8	c4_08930-c4_11878	50.6	44.9-53.8	11.161	1.9054	<0.0001	0.0809
	5-2	c5_01587-c5_02900	9.7	7.3-13.7	13.6697	1.91	<0.0001	0.1165
23RV	3-1	c3_00885-c3_01901	3	0.0-7.7	4.5576	0.8096	<0.0001	0.0218
	4-1	c4_00641-c4_02341	0	0.0-1.1	-8.1466	0.7852	<0.0001	0.1031
	4-7	c4_08930-c4_10609	36.7	32.7-42.4	-4.8106	0.8233	<0.0001	0.0319
	5-6	c5_06820-c5_07442	21.5	20.7-23.2	-13.0906	0.797	<0.0001	0.1807
	5-18	c5_24997-c5_26444	77.9	76.6-79.1	14.8201	0.8353	<0.0001	0.2702
33RV	1-17	MSAT127088-MSAT1.5	89.1	86.0-91.0	-12.7732	1.3187	<0.0001	0.0662
	2-4	IND628-MSAT2.36	21.3	16.5-26.3	9.3802	1.4237	<0.0001	0.0481
	2-10	MSAT2.10-MSAT2.22	64.4	60.4-64.4	-6.6995	1.2404	<0.0001	0.0577
	3-8	MSAT3.21-MSAT318406	50.3	40.0-55.9	7.4047	1.3091	<0.0001	0.0333
	4-1	MSAT4.39-MSAT4.8	1	0.0-4.2	11.5621	1.2665	<0.0001	0.1108
	4-6	MSAT4.15-CIW7	43.7	40.3-45.9	16.1257	1.3021	<0.0001	0.0801
	5-11	MSAT518662-MSAT520037	67.3	63.4-67.3	5.0839	1.2459	<0.0001	0.0003
	5-16	MSAT5.19-K9I9	84.9	83.2-86.9	-16.2971	1.2404	<0.0001	0.1271

Each QTL is designated by the relevant chromosome and the marker interval. For example, a QTL named 3-13 means that the QTL is located at the 13th marker interval of the 3rd chromosome.

Interval: the flanking markers of the QTL.

Position expressed in cM: the distance between the QTL and the first marker of the relevant chromosome.

RIL family: Denomination of the RIL families using the CRB codes (see Table 11 in Dataset S1 for correspondence).

Range expressed in cM: the support interval of the QTL position.

A: the estimated additive effect of the Col-0 allele for all RIL families, except for RIL family 33RV, for which the estimated additive effect corresponds to the Shahdara allele.

SE: standard error of the estimated QTL effect.

P-Value: p-value of the predicted QTL effect.

h^2(a): heritability of the additive effect.

Table 4. Epistatic QTLs for flowering time scored in the field experiment.

	QTL_i	interval_i	position_i	range_i	QTL_j	interval_j	position_j	range_j	AA	SE	P-Value
	1-18	c1_28454-c1_29898	103.6	101.0-105.6	5-15	c5_24997-c5_26671	90.30	87.8-92.1	-10.12	2.49	0.00
2RV	epistasis heritability										
	QTL_i	QTL_j	h^2 (aa)								
	1-18	5-15	0.0235								
	QTL_i	interval_i	position_i	range_i	QTL_j	interval_j	position_j	range_j	AA	SE	P-Value
	1-12	c1_18368-c1_20384	55.10	45.6-61.1	5-3	c5_02900-c5_04011	11.70	10.5-13.2	9.59	1.79	0.00
3RV	epistasis heritability										
	QTL_i	QTL_j	h^2 (aa)								
	1-12	5-3	0.04								
	QTL_i	interval_i	position_i	range_i	QTL_j	interval_j	position_j	range_j	AA	SE	P-Value
	4-1	c4_00641-c4_02133	0	0.0-2.1	5-3	c5_02900-c5_04011	14.10	10.7-19.5	9.96	1.71	0.00
4RV	epistasis heritability										
	QTL_i	QTL_j	h^2 (aa)								
	4-1	5-3	0.078								
	QTL_i	interval_i	position_i	range_i	QTL_j	interval_j	position_j	range_j	AA	SE	P-Value
	1-14	c1_20384-c1_23381	76.9	73.5-80.9	5-4	c5_04011-c5_05319	13.70	12.0-15.1	7.20	1.19	0.00
7RV	epistasis heritability										
	QTL_i	QTL_j	h^2 (aa)								
	1-14	5-4	0.0487								
	QTL_i	interval_i	position_i	range_i	QTL_j	interval_j	position_j	range_j	AA	SE	P-Value
	1-1	c1_00593-c1_02212	1.5	0.3-2.7	5-7	c5_07442-c5_08563	33.10	30.5-35.4	9.47	2.17	0.00
	1-1	c1_00593-c1_02212	1.5	0.3-2.7	4-10	c4_08930-c4_10609	47.90	44.7-51.2	13.23	2.26	0.00
	epistasis heritability										
8RV	QTL_i	QTL_j	h^2 (aa)								
	1-1	5-7	0.0091								
	1-1	4-10	0.0404								
	1-1	4-12	0.0009								
	QTL_i	interval_i	position_i	range_i	QTL_j	interval_j	position_j	range_j	AA	SE	P-Value
	4-11	c4_11878-c4_13171	50.6	42.9-53.7	5-17	c5_24997-c5_26671	83.00	80.0-85.4	-7.54	2.17	0.00
13RV	epistasis heritability										
	QTL_i	QTL_j	h^2 (aa)								
	4-11	5-17	0.0102								

Table 4. (continued)

	QTL_i	interval_i	position_i	range_i	QTL_j	interval_j	position_j	range_j	AA	SE	P-Value
19RV	1-19	c1_26993-c1_28454	98.6	96.8-102.7	4-1	c4_00641-c4_02133	0.10	0.0-0.7	5.35	1.29	0.00
	4-5	c4_05850-c4_06923	4.2	0.8-7.3	5-3	c5_02900-c5_04011	10.70	7.4-16.9	-3.53	1.31	0.01
	epistasis heritability										
	QTL_i	QTL_j	h^2 (aa)								
	1-19	4-1	0.0172								
	4-1	5-3	0.0065								
	4-5	5-3	0.002								
	QTL_i	interval_i	position_i	range_i	QTL_j	interval_j	position_j	range_j	AA	SE	P-Value
21RV	4-1	c4_00012-c4_02133	5.4	0.0-11.0	5-2	c5_01587-c5_02900	9.70	7.3-13.7	7.49	2.20	0.00
	4-4	c4_04877-c4_05850	17	12.6-22.6	5-2	c5_01587-c5_02900	9.70	7.3-13.7	5.25	2.07	0.01
	epistasis heritability										
	QTL_i	QTL_j	h^2 (aa)								
	4-1	5-2	0.0613								
	4-4	5-2	0.0056								
	QTL_i	interval_i	position_i	range_i	QTL_j	interval_j	position_j	range_j	AA	SE	P-Value
23RV	4-1	c4_00641-c4_02341	0	0.0-1.1	5-18	c5_24997-c5_26444	77.90	76.6-79.1	3.42	0.84	0.00
	epistasis heritability										
	QTL_i	QTL_j	h^2 (aa)								
	4-1	5-18	0.014								
	QTL_i	interval_i	position_i	range_i	QTL_j	interval_j	position_j	range_j	AA	SE	P-Value
33RV	1-17	MSAT127088-MSAT1.5	89.1	86.0-91.0	5-16	MSAT5.19-K9I9	84.90	83.2-86.9	-5.55	1.33	0.00
	5-11	MSAT518662-MSAT520037	67.3	63.4-67.3	5-16	MSAT5.19-K9I9	84.90	83.2-86.9	-5.70	1.26	0.00
	epistasis heritability										
	QTL_i	QTL_j	h^2 (aa)								
	1-17	5-16	0.0177								
	5-11	5-16	0.0166								

QTL_i and QTL_j are the 2 QTLs involved in epistatic interactions. AA: the estimated additive x additive effect of the Col-0 allele for all RIL families, except for RIL family 33RV, for which the estimated additive x additive effect corresponds to the Shahdara allele. h^2(aa): heritability of the additive x additive effect. For other descriptors, see the legend of Table 3 in Dataset S1.

Table 5. Genotypes of near-isogenic lines validating additive QTLs found in the Bay-0 x Shahdara RIL family, following the heterogeneous inbred families (HIFs) strategy.

				HIF															
**Locus**	**Chr**	**cM**	**Mb**	**100**	**408**	**144**	**416**	**192**	**404**	**11**	**203**	**367**	**143**	**181**	**312**	**90**	**397**	**48**	**108**
MSAT100008	1	0	0.01	A	A	A	A	B	B	A	B	A	A	B	A	B	B	B	B
T1G11	1	3.5	1.24	B	A	A	A	A	B	A	B	A	A	B	A	B	B	B	B
F21M12	1	9.7	3.21	B	A	A	A	A	B	A	B	A	A	B	A	B	B	B	B
IND4992	1	15.4	4.99	B	A	A	A	A	B	A	B	A	A	B	A	B	B	B	B
IND6375	1	19	6.38	B	A	A	A	A	B	B	A	A	A	B	A	B	B	B	B
MSAT1.10	1	21.6	7.30	B	A	A	A	A	B	B	A	A	A	B	A	B	A	B	B
MSAT108193	1	26.6	8.19	B	A	B	A	A	B	A	A	A	A	B	A	B	C	B	A
NGA248	1	32.4	9.89	B	A	B	A	A	B	A	A	A	A	B	A	B	C	B	A
IND1136	1	38.7	11.36	B	A	B	A	A	B	A	A	B	A	B	A	B	C	B	A
T27K12	1	49.1	15.93	B	B	B	B	A	B	A	A	B	A	A	A	B	A	B	B
MSAT1.42	1	54.7	18.15	B	B	B	B	A	B	A	A	B	B	A	A	D	A	B	D
NGA128	1	61.3	20.63	B	B	B	B	A	B	A	A	B	B	A	A	A	A	B	B
IND2188	1	63.8	21.89	B	B	B	B	A	B	A	A	B	B	A	A	A	A	B	B
dCAPsAPR2	1	66.1	22.98	B	B	B	B	A	B	A	A	B	B	A	A	A	A	B	B
F5I14	1	69.6	24.37	B	B	B	B	A	B	A	A	B	A	B	A	A	A	B	B
MSAT1.13	1	76.3	25.83	B	B	B	B	A	B	A	A	B	A	B	B	A	A	B	B
MSAT127088	1	82.7	27.09	B	B	D	B	A	B	A	A	B	A	B	B	A	A	B	B
MSAT1.5	1	91.3	29.02	C	C	B	B	A	B	A	A	B	B	A	B	A	B	A	B
MSAT2.5	2	0	0.21	B	B	A	A	A	B	B	B	A	B	A	B	B	B	A	B
MSAT200897	2	7.9	0.90	B	B	A	A	A	B	B	B	B	A	A	B	B	B	A	B
MSAT2.38	2	13	2.46	B	B	A	A	B	B	B	B	B	A	B	B	B	B	A	B
IND628	2	14.8	6.28	B	B	C	A	B	B	B	B	B	A	B	B	B	B	A	B
MSAT2.36	2	27.1	8.69	B	A	C	A	B	B	B	B	B	A	B	B	B	A	A	B
MSAT2.41	2	35	11.10	B	A	C	A	B	B	B	A	B	A	B	B	B	A	A	B
CZSOD2	2	38.1	11.95	B	A	B	A	B	B	B	A	B	A	B	B	B	A	A	B
MSAT2.7	2	42.9	13.19	B	A	C	B	B	B	B	A	B	A	B	B	B	A	A	B
IND216199	2	51.5	16.20	B	A	B	C	B	B	B	C	B	A	B	B	B	A	A	B
MSAT2.10	2	57.9	18.02	B	A	B	C	B	B	B	A	B	A	A	B	B	A	A	B
MSAT2.22	2	64.6	19.63	B	A	B	C	B	B	B	A	B	A	A	B	B	A	B	B
NGA172	3	0	0.79	A	A	B	A	B	A	B	B	B	B	A	A	B	B	A	A
MSAT3.99	3	3.2	1.51	A	A	B	A	B	A	B	B	B	B	B	A	B	B	A	A
ATHCHIB2	3	6.6	3.96	A	A	B	B	B	A	B	B	A	B	B	A	B	B	A	A
MSAT305754	3	7.9	5.75	A	A	B	B	B	A	B	B	A	B	B	A	B	B	A	A
MSAT3.19	3	23.2	8.81	B	A	A	B	A	A	B	A	A	B	B	A	B	B	A	A
MSAT3.117	3	28.8	9.86	B	A	A	A	C	B	B	A	A	A	B	A	B	B	A	A
MSAT3.32	3	39.5	11.21	B	A	A	A	C	C	A	A	B	A	A	A	B	B	A	A
MSAT3.21	3	48	17.28	B	B	A	A	C	C	A	A	B	A	A	A	B	B	A	A
MSAT318406	3	53.3	18.41	B	B	A	A	C	C	A	A	B	A	A	A	B	D	A	A
MSAT3.65	3	59	19.84	B	B	A	A	B	A	A	A	B	A	A	A	B	B	A	A
MSAT3.18	3	64.1	21.39	B	B	A	A	B	A	A	A	B	A	A	A	B	A	A	A
MSAT3.70	3	72.2	23.45	A	B	A	A	B	A	A	A	A	A	A	A	B	A	A	A
MSAT4.39	4	0	0.09	A	A	B	B	A	A	A	C	C	C	B	B	B	A	B	B
MSAT4.8	4	2	0.41	A	A	B	B	A	A	A	C	C	C	C	C	B	A	B	B
MSAT4.43	4	10.7	2.58	A	A	B	B	A	A	C	A	B	C	B	C	B	B	B	B
NGA8	4	15.8	5.63	A	A	B	B	B	A	C	A	B	C	B	C	B	B	B	A
MSAT4.35	4	24.2	7.55	A	A	B	A	B	A	C	A	B	B	B	B	A	B	B	A
MSAT4.15	4	33.5	9.36	A	A	B	A	B	A	B	A	B	A	B	B	B	B	B	B
CIW7	4	45	11.52	A	A	B	A	B	A	B	A	A	A	B	B	D	B	B	B
MSAT4.18	4	47	11.97	A	A	B	A	B	A	B	A	A	A	B	B	C	B	B	B
MSAT4.9	4	55.6	15.79	A	A	B	A	B	A	B	A	A	A	A	B	A	B	C	B
MSAT4.68	4	61.8	17.26	A	A	B	A	B	B	B	A	A	B	A	B	A	B	C	A
MSAT4.37	4	69.1	18.34	A	A	A	A	B	B	B	A	A	B	A	B	B	B	A	B
MSAT500027	5	0	0.03	A	B	A	B	A	B	A	A	B	A	B	B	B	A	A	B
NGA225	5	8.8	1.51	A	B	A	B	A	B	A	A	B	A	B	B	B	A	A	B
NGA249	5	12.1	2.77	A	B	B	B	A	B	A	A	B	A	B	B	A	A	A	B
NGA151	5	17.7	4.67	A	A	B	B	A	B	A	A	B	A	B	B	A	A	A	B
MSAT5.14	5	26.6	7.50	A	B	B	B	A	A	A	A	B	A	A	B	A	A	A	A

Table 5. (continued)

				HIF															
**Locus**	**Chr**	**cM**	**Mb**	**100**	**408**	**144**	**416**	**192**	**404**	**11**	**203**	**367**	**143**	**181**	**312**	**90**	**397**	**48**	**108**
NGA139	5	30.4	8.43	B	B	B	A	A	A	A	A	B	A	A	B	A	A	A	A
MSAT512110	5	41.8	12.11	B	B	B	A	A	A	B	A	B	A	A	B	A	A	B	A
MSAT5.22	5	45.4	13.96	B	B	B	A	A	B	B	A	B	A	A	B	A	A	B	A
MSAT5.59	5	51.5	15.46	B	B	B	A	A	B	B	A	B	A	A	B	A	B	B	A
MSAT5.9	5	57.8	17.25	B	B	B	A	A	B	B	A	B	A	A	B	A	A	B	A
MSAT518662	5	62.3	18.66	B	B	B	A	A	B	A	A	B	A	A	B	A	C	C	A
MSAT520037	5	67.4	20.04	B	B	B	A	A	B	A	A	A	A	A	B	A	A	C	A
MSAT5.12	5	71.6	21.42	A	B	B	A	B	B	A	A	A	A	A	B	A	B	A	C
JV6162	5	74.2	22.53	A	B	B	A	B	B	A	A	A	A	A	B	A	B	A	C
JV7576	5	79.1	23.88	A	B	B	A	B	B	A	A	A	A	A	B	A	B	A	C
MSAT5.19	5	85	25.92	B	A	B	A	B	B	A	A	A	A	A	B	A	B	B	C
K9I9	5	91.2	26.98	B	B	B	A	B	C	B	A	A	A	A	B	A	D	B	A

A: Shahdara genetic background; B: Bay-0 genetic background; C: heterozygous (genetic markers delimiting the polymorphic region for every HIF validating additive QTL found in the Bay-0 x Shahdara RIL family); D: not available.

Table 6. Effect of *Arabidopsis thaliana* plant genotype on flowering time in near-isogenic lines, validating additive QTLs found in the Bay-0 x Shahdara RIL family, following the heterogeneous inbred families (HIFs) strategy.

		Factors																						
		block			block x Bay-Sha			Bay-Sha			family(Bay-Sha)			Lov-5			Bg-2			Error		LSM PTT (PTU)	
QTL	HIF	d.f.	MS	F	d.f.	MS	F	d.f.	MS	F	d.f.	MS	F	d.f.	MS	F	d.f.	MS	F	d.f.	MS	Bay	Sha
QTL1.17	100	2	9.3	0.05 ns	2	40.9	0.23 ns	1	3452.4	19.67 ***	2	121.0	0.69 ns	1	415.0	2.36 ns	1	595.3	3.39 ns	36	175.5	455.7	438.1
QTL1.17	408	2	733.0	2.31 ns	2	182.6	0.57 ns	1	3793.5	11.95 ***	2	641.4	2.02 ns	1	126.8	0.40 ns	1	323.0	1.02 ns	36	317.6	463.3	444.9
QTL2.4	144	2	725.0	1.59 ns	2	746.4	1.64 ns	1	2595.4	5.71 *	1	132.3	0.29 ns	1	1893.3	4.16 ns	1	210.9	0.46 ns	25	454.8	421	441.7
QTL2.10	416	2	730.0	4.81 *	0	-	-	1	12749.9	84.07 ***	1	6.3	0.04 ns	1	313.9	2.07 ns	1	1.3	0.01 ns	17	151.6	528.9	439.2
QTL3.8	192	2	829.5	2.02 ns	2	81.4	0.20 ns	1	8446.1	20.52 ***	2	1106.7	2.69 ns	1	5.9	0.01 ns	1	4782.3	11.62 **	36	411.6	404.9	433.1
QTL3.8	404	2	1107.1	5.98 **	2	512.8	2.77 ns	1	1155.8	6.24 *	2	254.8	1.38 ns	1	414.3	2.24 ns	1	65.7	0.35 ns	36	185.2	478.2	488.4
QTL4.1	11	2	1297.5	2.91 ns	2	1092.7	2.45 ns	1	2123.2	4.76 *	-	-	-	1	26.3	0.06 ns	1	2992.7	6.71 *	15	446.2	365.8	388.2
QTL4.1	203	2	295.9	0.70 ns	2	583.6	1.39 ns	1	7650.1	18.17 ***	2	1192.0	2.83 ns	1	131.5	0.31 ns	1	1304.2	3.10 ns	35	421.0	412.8	439.2
QTL4.1	367	2	964.4	4.21 *	2	600.9	2.63 ns	1	6394.8	27.94 ***	2	353.8	1.55 ns	1	1035.3	4.52 *	1	40.7	0.18 ns	35	228.8	439.7	463.8
QTL4.1	143	2	69.7	0.47 ns	2	7.7	0.05 ns	1	793.3	5.31 *	2	79.8	0.53 ns	1	694.8	4.65 *	1	82.6	0.55 ns	35	149.5	440.1	448.8
QTL4.1	181	2	3763.0	4.69 *	2	750.0	0.93 ns	1	12851.8	16.00 ***	2	1188.4	1.48 ns	1	469.6	0.58 ns	1	47.7	0.06 ns	33	803.0	354	389.5
QTL4.1	312	2	1451.3	4.56 *	2	80.0	0.25 ns	1	4201.6	13.21 ***	2	213.3	0.67 ns	1	677.6	2.13 ns	1	0.1	0.00 ns	35	318.1	421.2	440.6
QTL4.6	90	2	416.7	0.57 ns	2	402.5	0.55 ns	1	3556.7	4.89 *	2	2965.3	4.08 *	1	1857.7	2.55 ns	1	806.6	1.11 ns	31	727.3	377.2	396.5
QTL5.11	397	2	492.7	3.54 ns	2	116.2	0.83 ns	1	1129.5	8.11 *	-	-	-	1	666.4	4.79 *	1	80.6	0.58 ns	14	139.2	439.4	455.5
QTL5.11	48	2	1470.0	5.37 *	2	151.3	0.55 ns	1	1378.2	5.04 *	2	535.4	1.96 ns	1	15.3	0.06 ns	1	153.5	0.56 ns	28	273.6	427.1	414.6
QTL5.16	108	2	4514.2	7.80 **	2	3540.7	6.12 *	1	7251.7	12.53 **	-	-	-	1	3981.4	6.88 *	1	2398.4	4.14 ns	14	578.8	463.5	426.5

LSM PTT: Least-square means of photothermal time (expressed in photothermal units) for Bay-0 and Shahdara allele.

d.f.: Degrees of freedom; MS: mean square; *0.05 > P > 0.01, **0.01 > P > 0.001, ***P < 0.001, ns: not significant.

A ‘family(Bay-0-Sha)’ factor controls for the effect of “independent fixed plant” during the process of NIL creation (see Materials and Methods section). ‘-‘ indicates that only 1 family was available in both genetic backgrounds.

Table 7. Comparison of the QTLs detected in this study and the QTLs found in Simon et al, 2009 (shaded area of the table). The comparison is presented for the 5 RIL families that were used in Simon *et al.* (2008). "CI" stands for "Confidence Interval". Values for Simon *et al.* (2008) were obtained by re-analyzing their data with QTLnetwork 2.0. The "Overlapping" column contains a "X" symbol whenever QTLs found in both study overlap.

family	Flowering time measured in the field 07-08 (PTU)				Flowering time in GH conditions (Simon *et al*, 2008)				Overlapping
	QTL	position	CI	Col allele effect sign	QTL	position	CI	Col allele effect sign	
7RV					1-15	84.1	80.8-89.0	-	
					2-7	38.8	29.5-42.1	+	
					2-9	48.7	47.6-53.3	+	
	3-13	71.5	66.6-74.1	+					
	4-6	20.7	14.3-25.1	+					
	4-10	44.9	38.6-51.3	+					
					4-15	68.8	63.4-73.5	-	
	5-4	13.7	12.0-15.1	+					
					5-11	52.1	48.1-55.8	-	
	5-20	89.4	85.3-93.3	+	5-20	92.9	90.4-95.3	+	X
8RV	1-1	1.5	0.3-2.7	+	1-1	1.9	0.0-4.0	+	X
	1-23	135.8	130.2-137.9	+					
					3-13	57.9	55.1-64.1	+	
	4-12	57.9	52.7-61.5	+					
	5-3	17.3	14.0-22.5	-	5-3	16.9	9.5-22.7	-	X
	5-7	33.1	30.5-35.4	-					
					5-8	41.7	37.5-46.0	-	
	5-18	111.1	108.3-112.7	+	5-18	111	105.7-112.7	+	X
13RV	1-20	93.2	89.9-96.2	+					
	2-7	27.1	22.8-31.4	-					
	2-14	61.2	58.3-61.2	+					
	3-6	8	1.4-11.0	+					
	4-11	50.6	42.9-53.7	-					
	5-3	10.3	8.1-11.9	+	5-3	10.9	9.3-13.6	+	X
	5-17	83	80.0-85.4	+	5-17	84	79.8-87.6	+	X
20RV	1-4	8.3	0.0-11.8	-					
	1-10	41.9	40.3-45.9	-	1-9	40	35.1-45.9	-	X
					1-16	74.6	73.1-77.7	-	
					4-1	0	0.0-3.0	-	
	4-10	37.6	35.3-40.0	-	4-8	28.8	24.5-35.7	-	X
	5-4	10.7	9.6-14.9	+					
	5-17	74.3	70.9-76.8	-	5-16	72.8	65.9-75.9	-	X
21RV					1-6	36	29.1-48.7	-	
	3-13	67.5	61.1-70.1	+	3-11	51.3	37.5-66.8	+	X
					4-1	1.5	0.0-5.3	-	
	4-8	50.6	44.9-53.8	+					
	5-2	9.7	7.3-13.7	+					
					5-17	100.8	97.8-100.8	+	

Table 8. Enrichment ratios as a function of the number of top SNPs chosen in the GWA mapping results using EMMA. The mean and the corresponding 95% confidence interval from the null distributions (respectively mean, low and high) are presented for SNPs close to candidate genes (CG) and/or QTLs.

Number of top SNPs	Observed enrichment QTL	Expected enrichment QTL			Observed enrichment CG	Expected enrichment CG			Observed enrichment CG + QTL	Expected enrichment CG + QTL		
		Mean	Low	High		Mean	Low	High		Mean	Low	High
50	1.07	0.99	0.51	1.46	4.10	1.04	0.00	3.82	7.44	1.02	0.00	5.34
100	0.97	0.99	0.63	1.34	3.00	0.97	0.14	2.60	5.32	0.96	0.00	3.46
200	1.1	1.00	0.67	1.36	2.66	1.00	0.27	2.19	4.52	1.00	0.13	2.79
300	1.03	1.01	0.64	1.37	2.09	0.98	0.32	2.25	3.20	1.04	0.18	3.01
400	1.1	1.00	0.66	1.30	2.08	1.01	0.41	2.01	2.99	1.00	0.27	2.66
500	1.07	1.01	0.71	1.32	1.88	0.97	0.44	1.76	2.45	1.01	0.34	2.58
1000	1.06	1.00	0.77	1.25	1.61	0.99	0.63	1.54	1.97	1.01	0.45	1.89
2000	1.07	1.00	0.84	1.16	1.43	1.00	0.70	1.35	1.65	1.01	0.59	1.56
3000	1.08	1.00	0.86	1.12	1.37	1.00	0.71	1.33	1.60	1.00	0.66	1.43

Table 9. Field experiment candidate genes detected in phenotypes scored in greenhouse conditions. 'EMMA' and 'Wilc': candidate genes detected in the top 500 SNPs by the EMMA and Wilcoxon methods, respectively. 'overlapping QTLs': RIL family names with a QTL overlapping the position of the gene. 'pathway': genetic pathway the candidate genes might be implicated in (information obtained from the TAIR website and literature searches). The 16 remaining columns indicate whether those genes were detected for different greenhouse phenotypes in Atwell *et al.* (2010). LD: Flowering time in long days at 18°C; LDV: Flowering time in long days at 18°C, vernalized for 2 weeks at 4°C; SD: Flowering time in short days at 18°C; SDV: Flowering time in short days at 18°C , vernalized for 2 weeks at 4°C; 0W: bolting time in long days, 23°C, not vernalized; 2W: bolting time in long days, 23°C, vernalized for 2 weeks at 5°C; 4W: bolting time in long days, 23°C, vernalized for 4 weeks at 5°C; 8W: bolting time in long days, 23°C, vernalized for 8 weeks at 5°C; FLC: FLC expression, long days, 20-22°C; FRI: FRI expression, long days, 20-22°C; 8WGHFT: Flowering time in natural light (from October 2002 to March 2003) at 20-22°C, vernalized for 8 weeks at 4°C; 8WGHFT: Flowering time in natural light (from October 2002 to March 2003) at 20-22°C, not vernalized; FTGH: flowering time in greenhouse conditions, long day 20°C.

Gene	chr.	EMMA	Wilc.	overlapping QTLs	Pathway	LD	LDV	SD	SDV	0W	2W	4W	8W	FLC	FRI	FT10	FT16	FT22	8WGHFT	0WGHFT	FTGH
**TSF**	4	X	X	7,8,13,20,21,33	floral integrator																
**AT4G23340**	4	X	X	4,13,19	gibberellin?																
**ELF5**	5	X	X	2,17	independent of FLC																
**ETC3**	4	X	X	2,33	trichome and root hair patterning	X		X		X	X	X		X	X		X	X		X	X
**AT2G30810**	2	X	X	-	gibberellin?	X															
**AGL 18**	3	X	X	-	upstream of floral integrators										X						
**AT2G47310**	2	X	X	-	autnomous? Similar to FCA						X		X						X		
**ATH1**	4	X	X	-	gibberellin																
**KNAT5**	4	X	X	-	gibberellin																
**AT4G33280**	4	X	X	-	vernalization? Similar to VRN1	X	X	X	X			X	X	X		X	X	X	X		
**AT3G57300**	3	X	X	-	?																
**TOC1**	5	X		2,6,7,8,19,23,33	circadian clock																
**ATHAP2B**	3	X		13,17,23	osmotic stress																
**YAP169**	5	X		13,19,21	gibberellin										X						X
**LD**	4	X		3,17,19	autnomous					X						X	X				
**FCA**	4	X		7,23	autonomous																
**PHYD**	4	X		7,23	light-dependent																X
**AT2G39540**	2	X		3,17	gibberellin																
**KIN1**	5	X		20	cold and ABA inducible protein																

Table 9. (continued)

Gene	chr.	EMMA	Wilc.	overlapping QTLs	Pathway	LD	LDV	SD	SDV	0W	2W	4W	8W	FLC	FRI	FT10	FT16	FT22	8WGHFT	0WGHFT	FTGH
**CDF3**	3	X		33	circadian clock																
**KIN2**	5	X		20	cold and ABA inducible protein																
**COL1**	5	X		20	circadian clock								X								
**CKB1**	5	X		-	circadian clock																
**DDF2**	1	X		-	gibberellin																
**CCA1**	2	X		-	circadian clock																
**AP1**	1	X		-	floral meristem identity																
**TOR1**	4	X		-	?																
**GI**	1	X		-	circadian clock	X															
**PMI15**	5	X		-	light-dependent																
**SRR1**	5		X	2,6,7,8,13,23,33	circadian clock																
**AT5G59570**	5		X	7,8,13,20,21,33	circadian clock? associated to LUX																
**FY**	5		X	1,8,19,20	autonomous																
**GA1**	4		X	3,17,19	gibberellin	X				X											
**MMP**	1		X	2,13,19	senescence-associated																
**ATGA2OX7**	1		X	3	gibberellin			X	X	X	X	X	X					X	X		
**LKP2**	2		X	13	circadian clock			X													
**CUL4**	5		X	33	light-dependent								X		X						
**DDF1**	1		X	20	gibberellin			X	X												X
**FPA**	2		X	33	autonomous																
**GASA5**	3		X	-	gibberellin								X			X	X		X	X	
**AGL16**	3		X	-	?		X														
**AGL17**	2		X	-	light-dependent	X	X			X	X	X		X		X	X	X	X	X	X

Table 10. List of candidate regions associated with flowering time scored in the field with no obvious candidate gene.

GWA method	Chromosome	Position (bp)
EMMA	1	13,410,964
EMMA	1	27,060,739
EMMA	2	9,762,418
EMMA	3	10,183,643
EMMA	3	3,273,516
EMMA	5	2,402,568
EMMA	5	1,993,499
EMMA	5	18,617,347
Wilcoxon	3	16,045,391

Table 11. Names, crosses and number of lines for each RIL family phenotyped during the field experiment.

RIL families CRB code[a]	Crosses	Number of lines	Number of genotyped markers
2RV	Bla-1 x Col-0	259	78
3RV	Tsu-0 x Col-0	276	79
4RV	Nok-1 x Col-0	223	85
6RV	Ri-0 x Col-0	286	85
7RV	Ct-1 x Col-0	377	85
8RV	Cvi x Col-0	366	90
13RV	Shahdara x Col-0	345	96
17RV	Ge-0 x Col-0	338	92
19RV	Can-0 x Col-0	371	94
20RV	Bur-0 x Col-0	343	97
21RV	Blh-1 x Col-0	315	75
23RV	Yo-0 x Col-0	456	82
33RV	Bay-0 x Shahdara	411	69

[a] CRB = Centre de Ressources Biologiques (INRA Versailles)

Table 12. List of the 282 *a priori* candidate genes for flowering time.

Name	LocusTag	Start	End
LHY	AT1G01060	33379	37840
CRY2	AT1G04400	1185549	1188516
CKL13	AT1G04440	1202254	1205802
STO	AT1G06040	1828412	1829889
PAP3	AT1G09530	3076584	3079541
PHYA	AT1G09570	3095258	3100359
HYL1	AT1G09700	3137769	3140355
AT1G10588	AT1G10588	3501146	3501905
DDF1	AT1G12610	4289942	4291015
LWD1	AT1G12190	4394895	4396289
RAV1	AT1G13260	4542165	4543739
GAI	AT1G14920	5149221	5151349
GA4	AT1G15550	5344473	5346161
ATARP4	AT1G18450	6348100	6351968
AT1G22690	AT1G22690	8027287	8028114
GI	AT1G22770	8061833	8067705
SEPALLATA3	AT1G24260	8593631	8596087
PFT1	AT1G25540	8969052	8974647
CAL	AT1G26310	9100140	9103590
ATGA2OX2	AT1G30040	10537632	10539815
UFO	AT1G30950	11036161	11037489
AT1G30960	AT1G30960	11037593	11040014
SUF4	AT1G30970	11040262	11043732
FRL2	AT1G31814	11412589	11414483
VIP1	AT1G43700	16486671	16488681
ATGA20OX5	AT1G44090	16763117	16764926
CH1	AT1G44446	16850799	16853664
GCR1	AT1G48270	17831621	17834088
RTV1	AT1G49480	18317846	18320313
ATGA2OX7	AT1G50960	18893217	18895387
AT1G52800	AT1G52800	19667712	19669030
SPA4	AT1G53090	19787020	19790570
SPL4	AT1G53160	19810087	19811276
AT1G55080	AT1G55080	20556678	20557921
ORTH2	AT1G57820	21417835	21421611
ARR3	AT1G59940	22069282	22070638
ATGA20OX4	AT1G60980	22456238	22457805
VIP5	AT1G61040	22486872	22489634
ATSCO1	AT1G62750	23237099	23240112
LDL1	AT1G62830	23268155	23270867
DDF2	AT1G63030	23371072	23372075
FT	AT1G65480	24335091	24337597
RGL1	AT1G66350	24751858	24753706
FKF1	AT1G68050	25512339	25514552
AP1	AT1G69120	25985993	25989976
AT1G69935	AT1G69935	26345464	26346789
MMP	AT1G70170	26427537	26429019
AGL12	AT1G71692	26956307	26958789
AT1G72050	AT1G72050	27118686	27121132

Name	LocusTag	Start	End
HAP2C	AT1G72830	27409118	27411630
MIF1	AT1G74660	28051237	28051788
AT1G74670	AT1G74670	28056947	28057810
ASHH1	AT1G76710	28794623	28797570
FLM	AT1G77080	28960531	28964990
EFS	AT1G77300	29044816	29053704
ATGA2OX1	AT1G78440	29516492	29517944
NUA	AT1G79280	29824069	29837871
GA2	AT1G79460	29895285	29899480
ELF7	AT1G79730	30005431	30008898
ATGA3OX4	AT1G80330	30202953	30204429
GA4H	AT1G80340	30205585	30207092
MOS3	AT1G80680	30328900	30333661
RGA1	AT2G01570	255248	257549
CAND1	AT2G02560	689787	697595
PKS1	AT2G02950	854946	856537
SEPALLATA4	AT2G03710	1129265	1131835
CR88	AT2G04030	1281838	1286101
ELF8	AT2G06210	2428900	2436684
AT2G14900	AT2G14900	6411257	6412412
PHYB	AT2G18790	8146963	8151512
LKP2	AT2G18915	8201654	8204565
FVE	AT2G19520	8463018	8466607
PIL5	AT2G20180	8711105	8713973
FIO1	AT2G21070	9047944	9050609
SVP	AT2G22540	9586954	9590973
AGL17	AT2G22630	9625452	9629037
CLF	AT2G23380	9962650	9967439
COL3	AT2G24790	10573977	10575224
ELF3	AT2G25930	11066113	11070402
BAS1	AT2G26710	11387570	11390690
ATC	AT2G27550	11780328	11781758
SYD	AT2G28290	12063290	12080160
TOE1	AT2G28550	12233028	12235620
AT2G30810	AT2G30810	13134903	13135743
COP1	AT2G32950	13985010	13990612
SPL3	AT2G33810	14312077	14313148
FES1	AT2G33835	14318863	14321776
ATGA2OX3	AT2G34555	14564067	14565776
FHY1	AT2G37678	15808543	15809871
SNZ	AT2G39250	16395964	16398151
AT2G39540	AT2G39540	16507944	16508319
HOS1	AT2G39810	16619878	16625135
ELF4	AT2G40080	16741372	16741990
SHP2	AT2G42830	17827332	17831090
PIF4	AT2G43010	17893504	17896127
FPA	AT2G43410	18032324	18038320
CKB4	AT2G44680	18433621	18435432
AGL6	AT2G45650	18811424	18813596

Table 12. (continued)

Name	LocusTag	Start	End
SOC1	AT2G45660	18814612	18818121
SPA1	AT2G46340	19029246	19034486
APRR9	AT2G46790	19239718	19242156
CCA1	AT2G46830	19252741	19255983
AT2G47310	AT2G47310	19430754	19434347
RFI2	AT2G47700	19559384	19561654
MBD9	AT3G01460	173323	182461
SEPALLATA2	AT3G02310	464286	467081
COL2	AT3G02380	487243	488700
GASA5	AT3G02885	638028	639062
ATVGT1	AT3G03090	700463	704776
AT3G04510	AT3G04510	1215642	1216964
FLK	AT3G04610	1250559	1254879
HST	AT3G05040	1401277	1408203
ATGID1A	AT3G05120	1430477	1432784
ATHAP2B	AT3G05690	1676552	1678938
AT3G06910	AT3G06910	2178636	2181203
AtPRMT4b	AT3G06930	2185149	2189393
COL9	AT3G07650	2441663	2444538
AT3G10185	AT3G10185	3145584	3146204
FLD	AT3G10390	3229298	3231824
ATMYB65	AT3G11440	3602099	3605110
SPY	AT3G11540	3631893	3637961
PIE1	AT3G12810	4065049	4074085
LDL2	AT3G13682	4479200	4481516
SPL5	AT3G15270	5140372	5141355
SPA3	AT3G15354	5169102	5172844
VRN1	AT3G18990	6548875	6551859
HAF2	AT3G19040	6567163	6575288
ATFYPP3	AT3G19980	6961742	6965114
DDL	AT3G20550	7174470	7177948
FIE	AT3G20740	7248815	7252458
AT3G21320	AT3G21320	7499059	7501847
TIC	AT3G22380	7912912	7919517
VRN5	AT3G24440	8876034	8878178
TEL1	AT3G26120	9547635	9550423
LWD2	AT3G26640	9794457	9795694
FUS3	AT3G26790	9855065	9857226
BR6OX2	AT3G30180	11813216	11816244
ATARP6	AT3G33520	14104642	14106535
PCL1	AT3G46640	17194075	17196203
CDF3	AT3G47500	17514985	17517043
REF6	AT3G48430	17946594	17951731
AMP1	AT3G54720	20265703	20268826
SMZ	AT3G54990	20384695	20387499
AGL16	AT3G57230	21188689	21191911
AT3G57300	AT3G57300	21210467	21218864
AGL18	AT3G57390	21244678	21246888
GIS	AT3G58070	21517590	21518631

Name	LocusTag	Start	End
SHP1	AT3G58780	21749437	21752884
PIL6	AT3G59060	21838955	21841484
CKB3	AT3G60250	22281312	22283088
AGL13	AT3G61120	22629234	22631466
PIL2	AT3G62090	22999522	23001684
ATGID1B	AT3G63010	23300400	23302461
CRP	AT4G00450	203471	211003
FRI	AT4G00650	269026	271503
AT4G00690	AT4G00690	281645	283129
ETC3	AT4G01060	460472	461085
EZA1	AT4G02020	886600	891955
LD	AT4G02560	1123490	1128421
GA1	AT4G02780	1237767	1244813
DFL2	AT4G03400	1497535	1499864
PDF2	AT4G04890	2476487	2482343
CRY1	AT4G08920	5724100	5727250
GASA2	AT4G09610	6074767	6075642
SPA2	AT4G11110	6771601	6777221
AGL14	AT4G11880	7143109	7147216
pEARLI 1	AT4G12480	7406102	7406934
COP9	AT4G14110	8132881	8134915
ELIP2	AT4G14690	8418278	8419258
FAR1	AT4G15090	8614063	8618142
AT4G15180	AT4G15180	8651406	8662587
ESD4	AT4G15880	9012660	9016131
PHYD	AT4G16250	9195617	9199501
FCA	AT4G16280	9206613	9214841
HAT4	AT4G16780	9449133	9450762
AT4G16810	AT4G16810	9459889	9462272
VRN2	AT4G16845	9476162	9479897
CKB2	AT4G17640	9825210	9827285
PHYE	AT4G18130	10042149	10046094
TSF	AT4G20370	11000782	11003007
ATGA2OX8	AT4G21200	11302695	11306611
ATGA3OX3	AT4G21690	11527241	11529072
EBS	AT4G22140	11727738	11730521
AGL19	AT4G22950	12023926	12027432
AT4G23340	AT4G23340	12195463	12196803
AGL24	AT4G24540	12670975	12674082
PGI1	AT4G24620	12708762	12712835
GA5	AT4G25420	12990894	12992468
FWA	AT4G25530	13038369	13042452
TOR1	AT4G27060	13581407	13585161
CIP7	AT4G27430	13718685	13723330
ATHXK1	AT4G29130	14352043	14355109
VIP3	AT4G29830	14597667	14599306
AT4G30200	AT4G30200	14786639	14790509
ATPRMT5	AT4G31120	15132017	15136645
FLP1	AT4G31380	15229791	15230724

Table 12. (continued)

Name	LocusTag	Start	End
CYP83B1	AT4G31500	15273477	15275316
KNAT5	AT4G32040	15494071	15496362
ATH1	AT4G32980	15914725	15918047
AT4G33280	AT4G33280	16047358	16049359
FD	AT4G35900	17004598	17006290
AP2	AT4G36920	17400844	17403329
HLS1	AT4G37580	17658606	17660872
AGL21	AT4G37940	17835689	17838615
BRI1	AT4G39400	18324655	18328820
FHL	AT5G02200	437458	438892
PRR7	AT5G02810	637895	641975
LCL1	AT5G02840	648702	651970
ATHB51	AT5G03790	1004982	1006372
TFL1	AT5G03840	1024640	1025811
ELF6	AT5G04240	1169545	1174879
CPD	AT5G05690	1702689	1706788
MYB33	AT5G06100	1837908	1840728
YAP169	AT5G07200	2243554	2245340
CHE	AT5G08330	2680745	2681814
FLC	AT5G10140	3173498	3179449
AT5G10625	AT5G10625	3358788	3359782
HY5	AT5G11260	3593381	3594993
EMF1	AT5G11530	3695863	3701549
HAP2A	AT5G12840	4050694	4053609
FY	AT5G13480	4326531	4331702
AGL15	AT5G13790	4449017	4450846
AT5G14920	AT5G14920	4826482	4827983
GASA4	AT5G15230	4944903	4946219
CO	AT5G15840	5171185	5172761
COL1	AT5G15850	5176094	5177900
KIN1	AT5G15960	5209901	5210730
KIN2	AT5G15970	5211914	5212668
FRL1	AT5G16320	5344505	5346022
TFL2	AT5G17690	5827173	5829684
ASP2	AT5G19550	6598019	6601821
NPH4	AT5G20730	7016447	7022115
HUA2	AT5G23150	7785838	7792492
APRR5	AT5G24470	8355954	8358876
FPF1	AT5G24860	8541781	8542452
TNY	AT5G25810	8986774	8987790
GA3	AT5G25900	9036021	9038409
AT5G27230	AT5G27230	9584095	9588052
ATGID1C	AT5G27320	9629090	9631213
AT5G28450	AT5G28450	10372942	10374194
LSH1	AT5G28490	10454397	10455200
PHYC	AT5G35840	14025056	14028994
TCH2	AT5G37770	15016084	15016849
PMI15	AT5G38150	15240346	15242177
CDF2	AT5G39660	15895927	15898272

Name	LocusTag	Start	End
XPB2	AT5G41360	16561568	16566508
CIP1	AT5G41790	16744758	16750075
CUL4	AT5G46210	18748645	18754037
AT5G46910	AT5G46910	19065007	19068107
LBA1	AT5G47010	19089236	19096561
CKB1	AT5G47080	19141839	19143838
PAT1	AT5G48150	19539481	19541924
AtPRMT4a	AT5G49020	19888477	19892146
EMF2	AT5G51230	20840962	20846790
AT5G51310	AT5G51310	20870080	20871944
AT2353	AT5G51810	21072414	21074034
PGM	AT5G51820	21080594	21085283
DFL1	AT5G54510	22148319	22150904
ZTL	AT5G57360	23258653	23261816
VIN3	AT5G57380	23263621	23266730
MSI1	AT5G58230	23573238	23575471
SRR1	AT5G59560	24017782	24019183
AT5G59570	AT5G59570	24021114	24022738
VIP2	AT5G59710	24074633	24079144
AT5G59845	AT5G59845	24128550	24129246
APRR3	AT5G60100	24215225	24218590
TOE2	AT5G60120	24225012	24228950
AGL8	AT5G60910	24519708	24523369
VIP4	AT5G61150	24620882	24624951
TOC1	AT5G61380	24692290	24695776
LFY	AT5G61850	24861521	24864159
AT5G62040	AT5G62040	24940036	24940935
CDF1	AT5G62430	25086319	25088160
ELF5	AT5G62640	25166659	25169767
LIP1	AT5G64813	25927505	25930122
AGL31	AT5G65050	25999480	26003552
MAF3	AT5G65060	26004655	26008541
MAF4	AT5G65070	26009486	26013360
AGL68	AT5G65080	26014730	26019691
AT5G65540	AT5G65540	26212915	26215548
ICU2	AT5G67100	26794220	26802330
