# Supplementary figures and images for: Linkage and Association Mapping of Arabidopsis thaliana Flowering Time in Nature
Source: PLoS Genet. 2010 May 6;6(5):e1000940. doi: 10.1371/journal.pgen.1000940 (PMC2865524; doi:10.1371/journal.pgen.1000940)

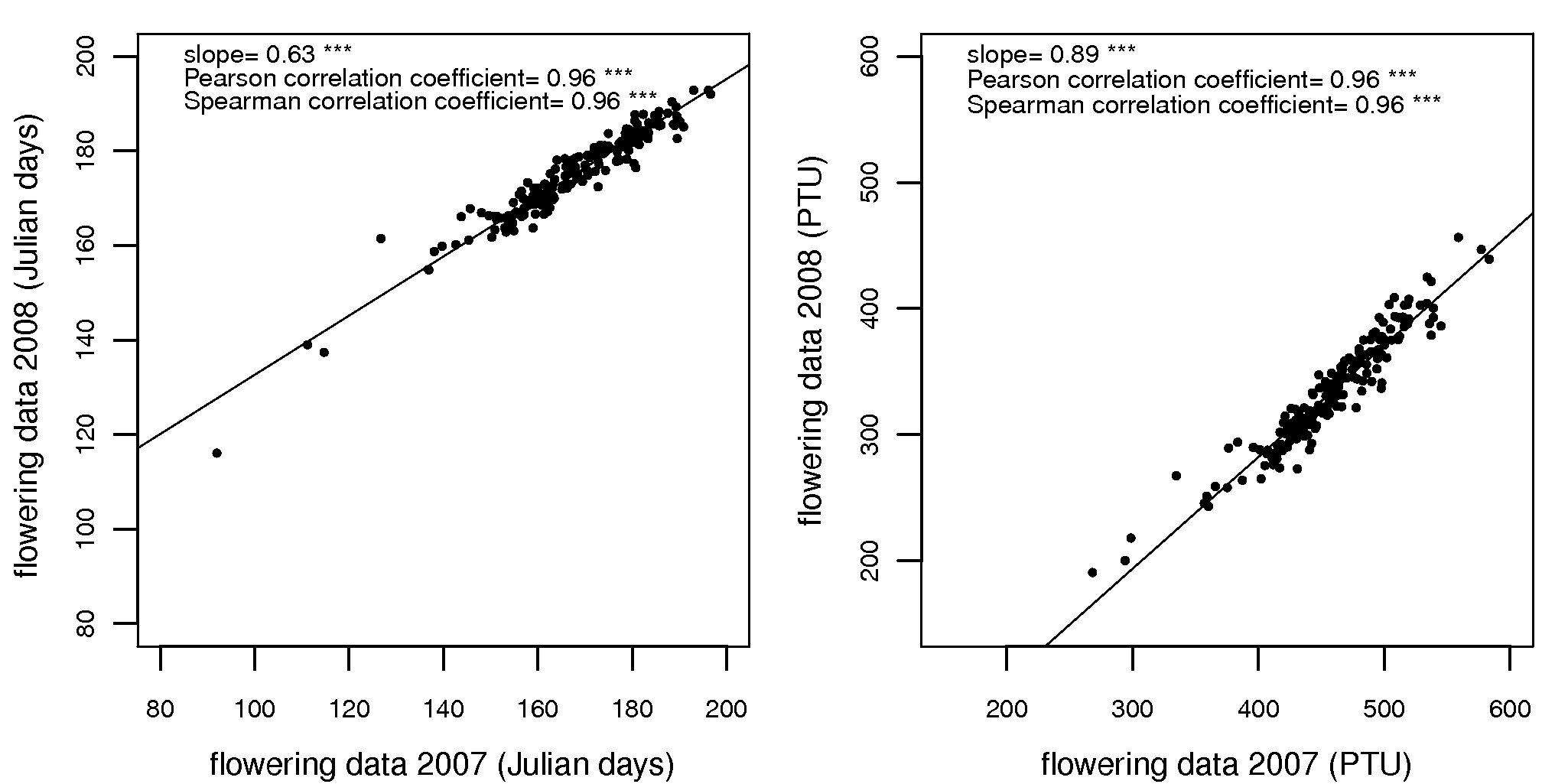

Supplement: Figure S1 — A year-to-year comparison of flowering time expressed in Julian days (left panel) and photothermal units (right panel). (0.06 MB TIF) [file pgen.1000940.s002.tif]

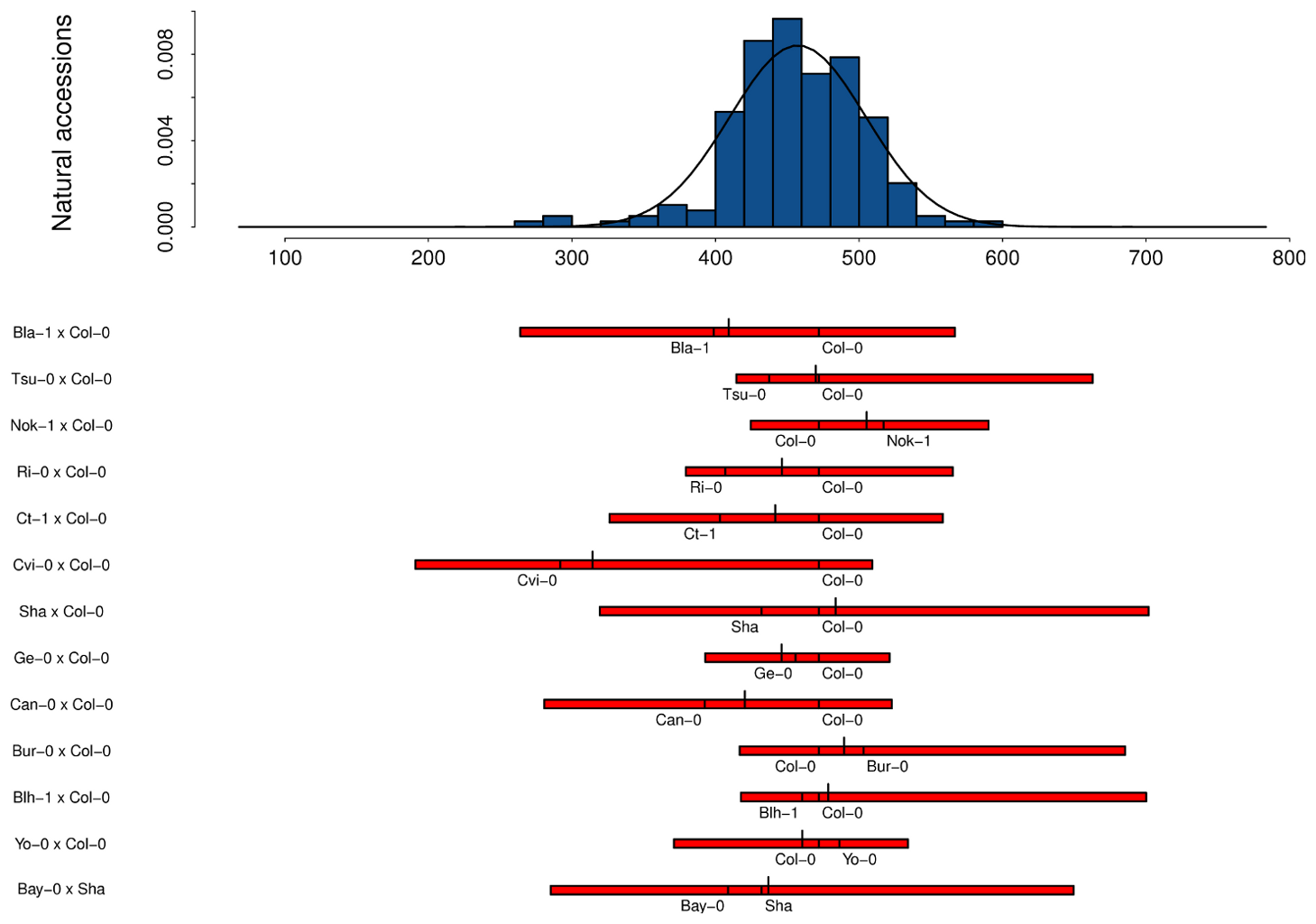

Supplement: Figure S2 — Distribution of flowering time expressed in photothermal units (PTU) for the 2007–2008 field experiment. Top: distribution of flowering time for the 197 natural accessions. Below: distribution of flowering time for each of the 13 RIL families. For each RIL family, red bars extend from the minimum to the maximum values observed, with the larger ticks demarcating the median of the distribution and the smaller ticks indicating the flowering times for the parental lines. (0.14 MB TIF) [file pgen.1000940.s003.tif]

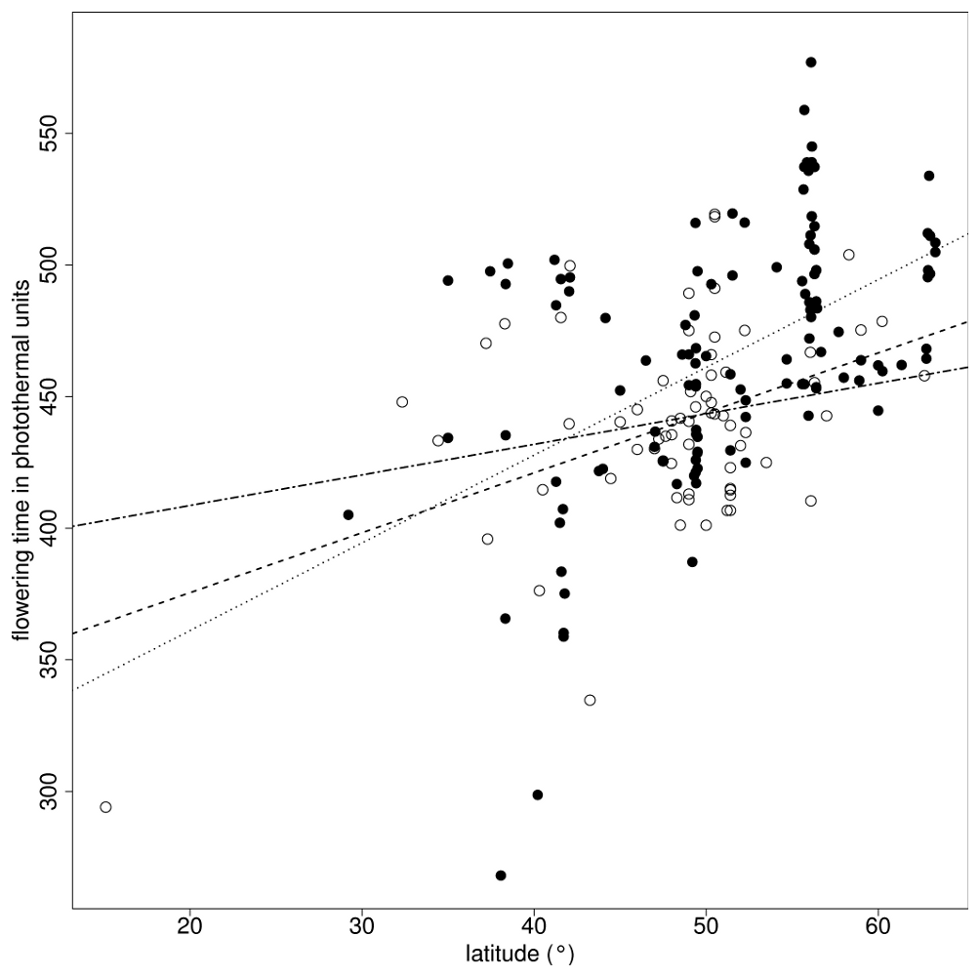

Supplement: Figure S3 — Relationship between flowering time and latitude. Accessions bearing non-functional and functional FRI alleles are depicted by empty and filled dots, respectively. Drawn lines correspond to regression lines for accessions with a functional FRI allele (dotted line), a non-functional FRI allele (dashed line), a non-functional FRI allele excluding Cvi-0 (i.e., latitude<20°C; dashed-dotted line). (0.12 MB TIF) [file pgen.1000940.s004.tif]

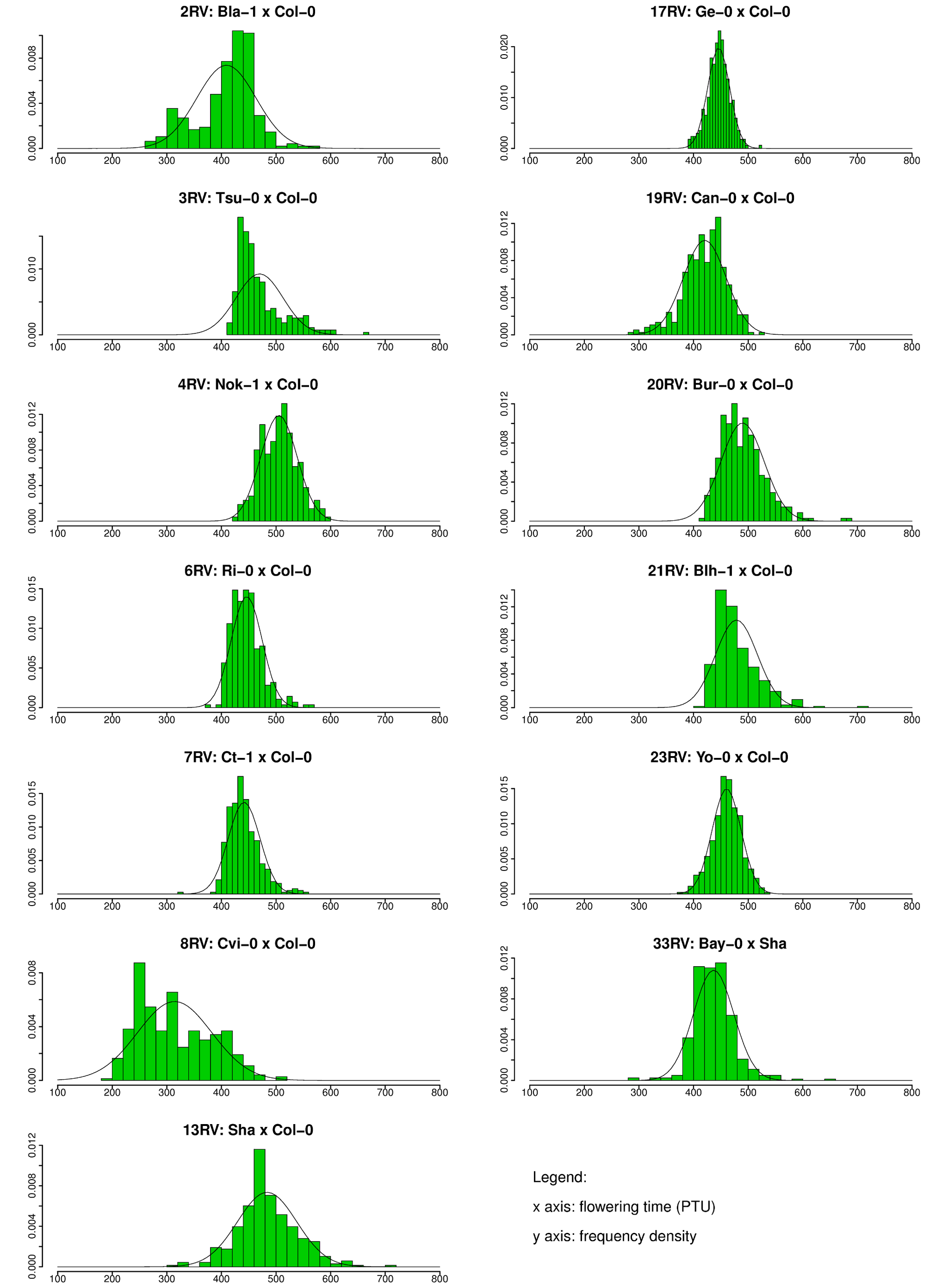

Supplement: Figure S4 — Distribution of flowering time for each RIL family. (0.69 MB TIF) [file pgen.1000940.s005.tif]

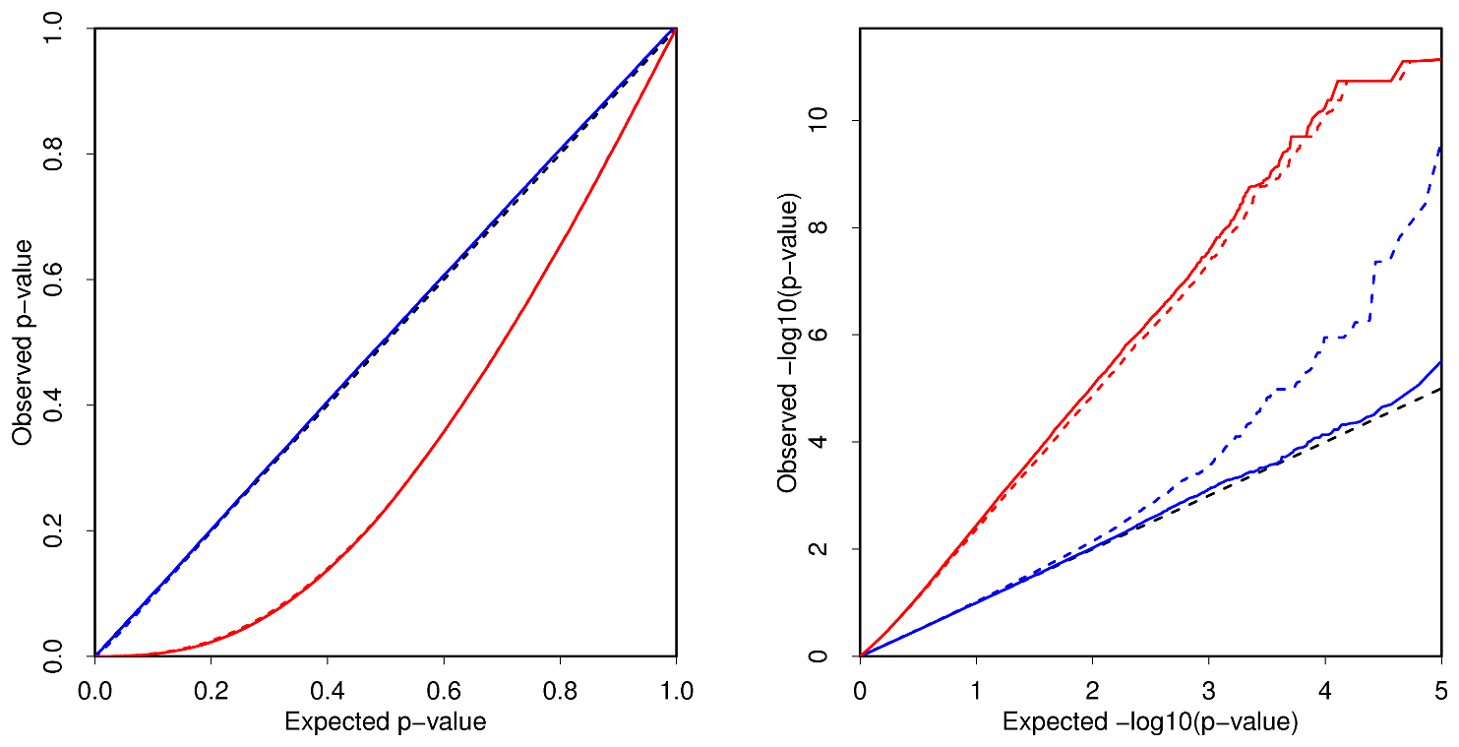

Supplement: Figure S6 — Quantile-Quantile plot of p-values (raw and negative logarithm) in genome-wide scans for flowering time. The different curves correspond to different analyses of GWA mapping. Dashed black line: expected; dashed red line: Wilcoxon; solid red line: Wilcoxon with minor allele relative frequency (MARF)>0.1; dashed blue line: EMMA; solid blue line: EMMA with MARF>0.1. (0.13 MB TIF) [file pgen.1000940.s007.tif]

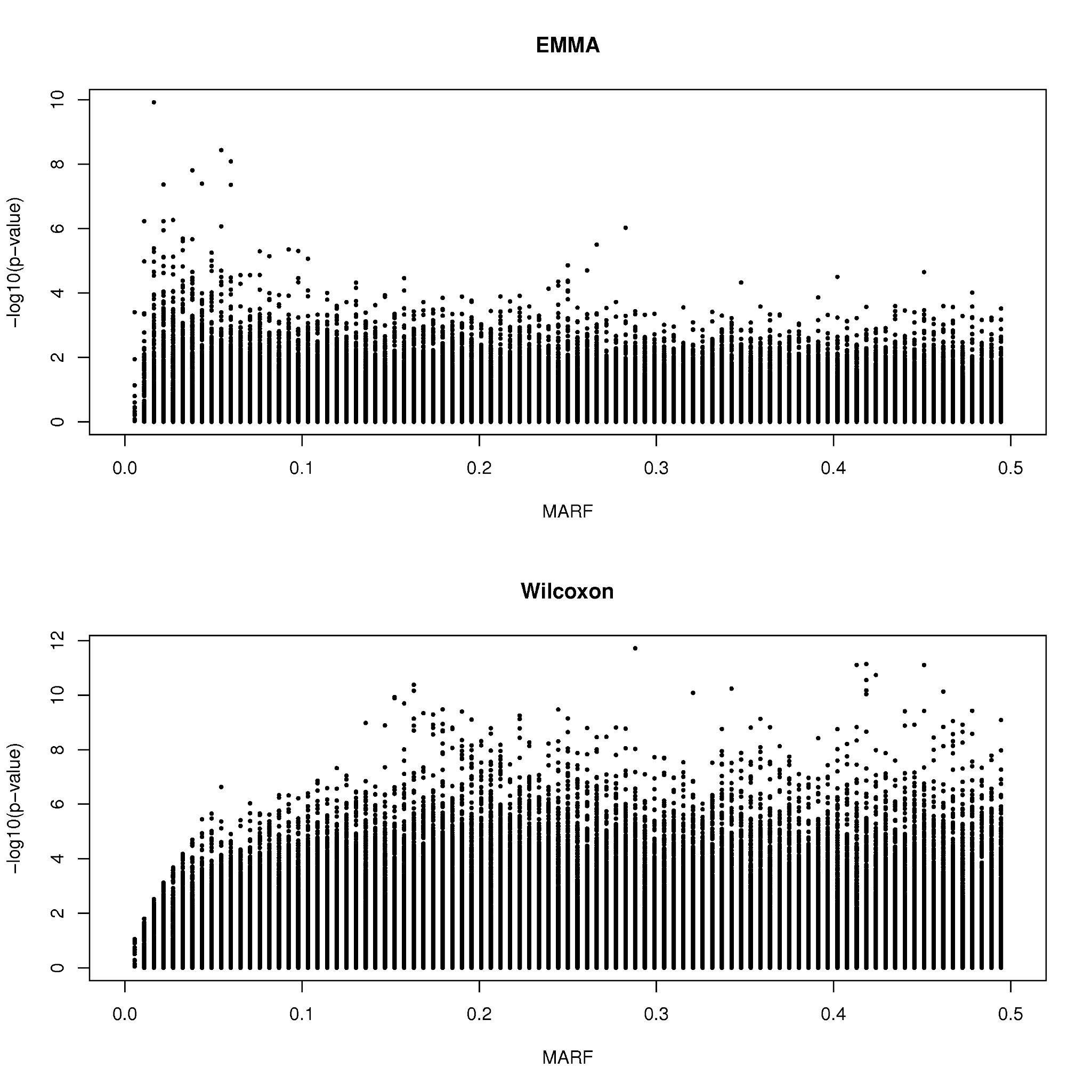

Supplement: Figure S7 — The distribution dependence of p-value distributions on minor allele relative frequency (MARF) for EMMA (top panel) and Wilcoxon (bottom panel). (0.51 MB TIF) [file pgen.1000940.s008.tif]

# Chromosome 1

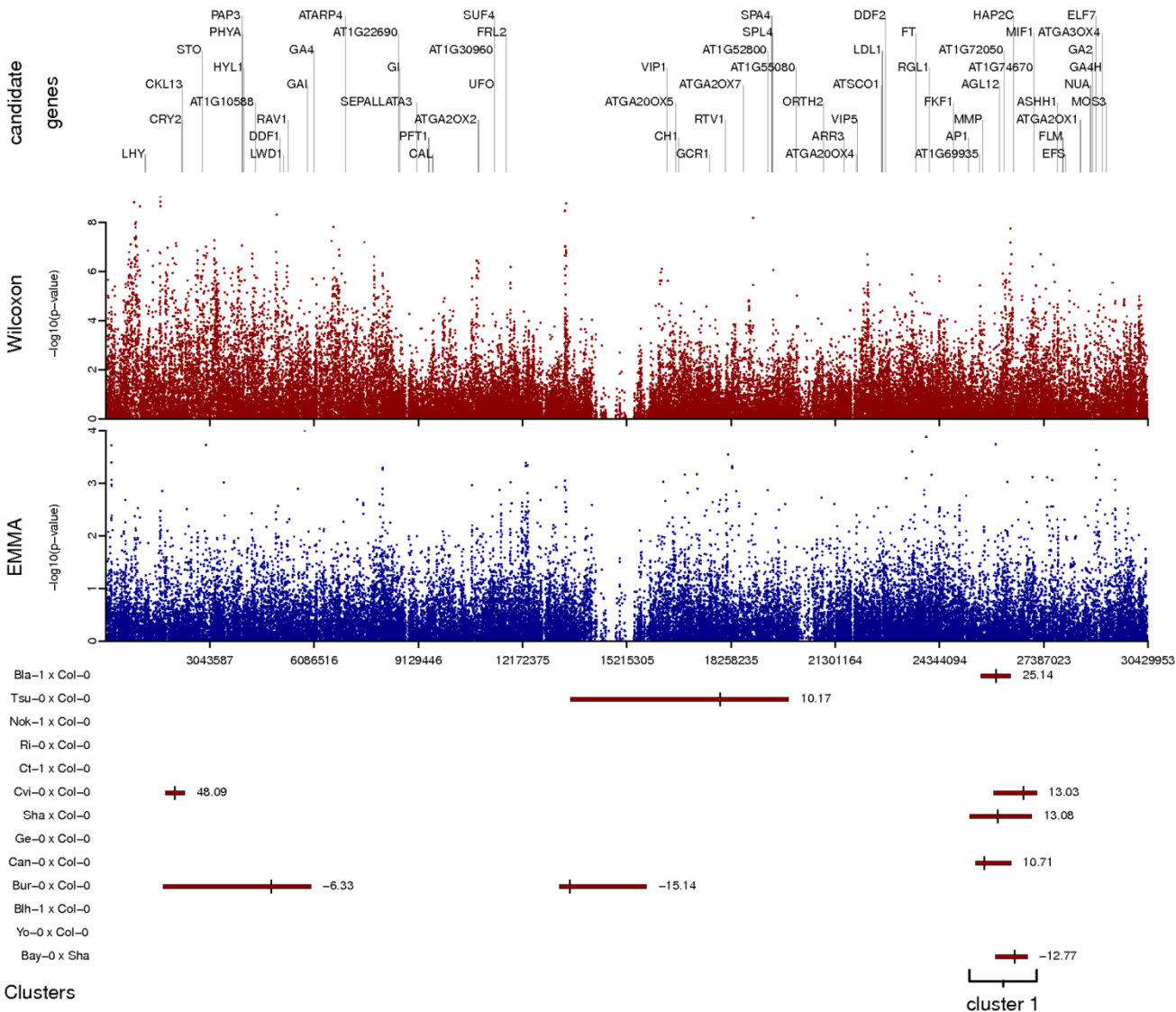

# Chromosome 2

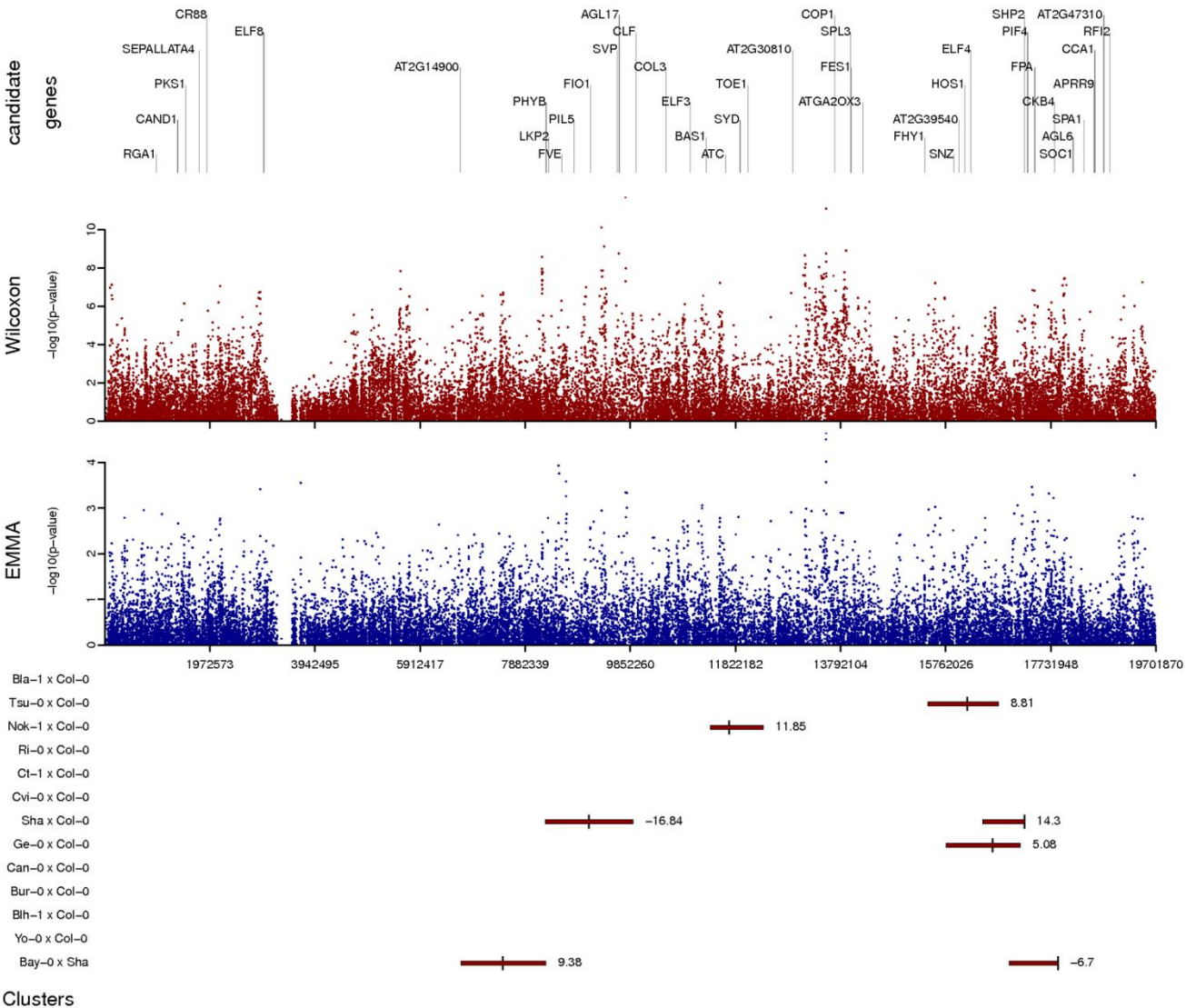

# Chromosome 3

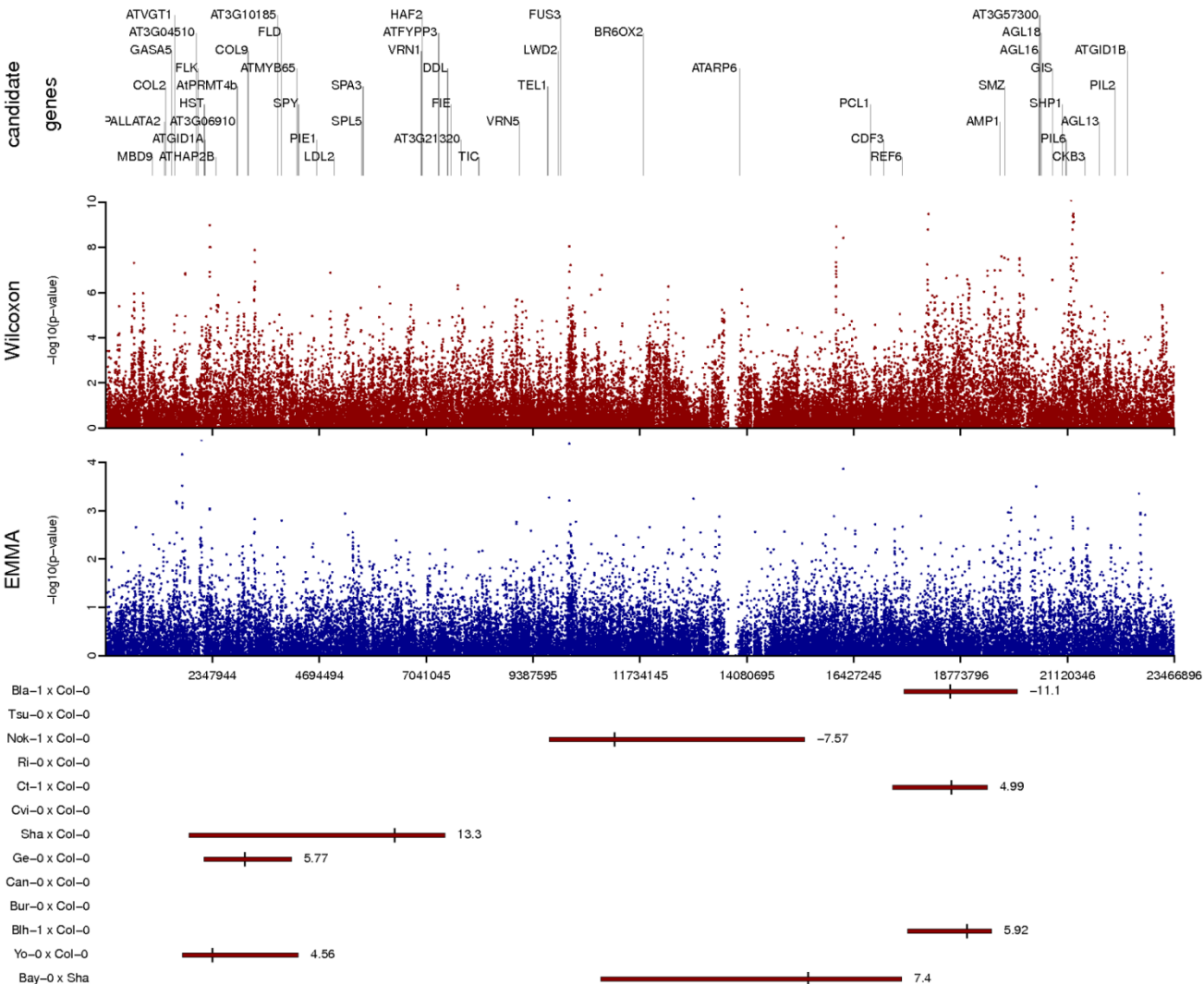

Clusters

## Chromosome 5

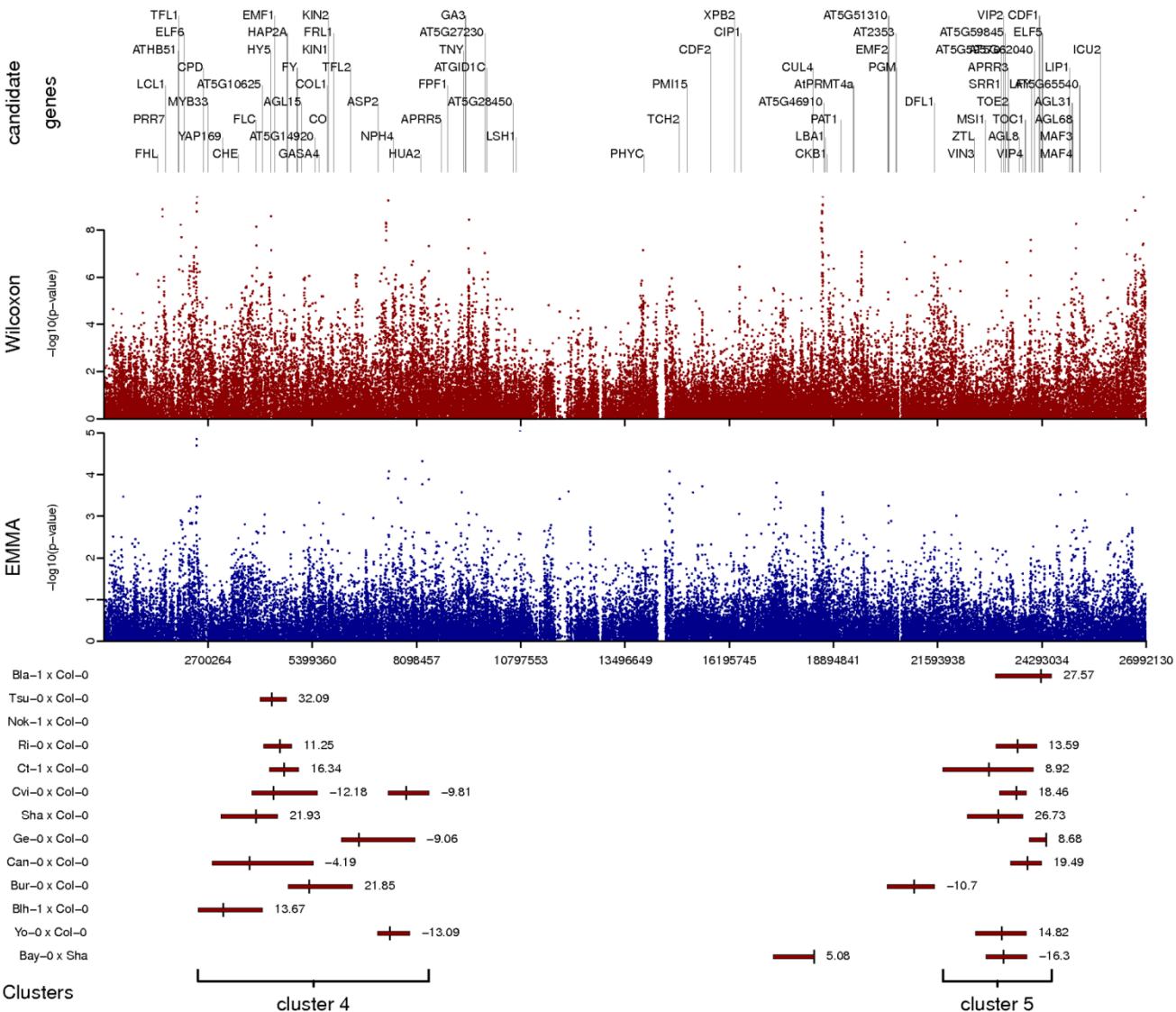

Supplement: Figure S8 — Comparison of GWA and traditional linkage mapping (additive QTLs) results for flowering time for chromosomes 1, 2, 3, and 5. (1.94 MB PDF) [file pgen.1000940.s009.pdf]

# Chromosome 1

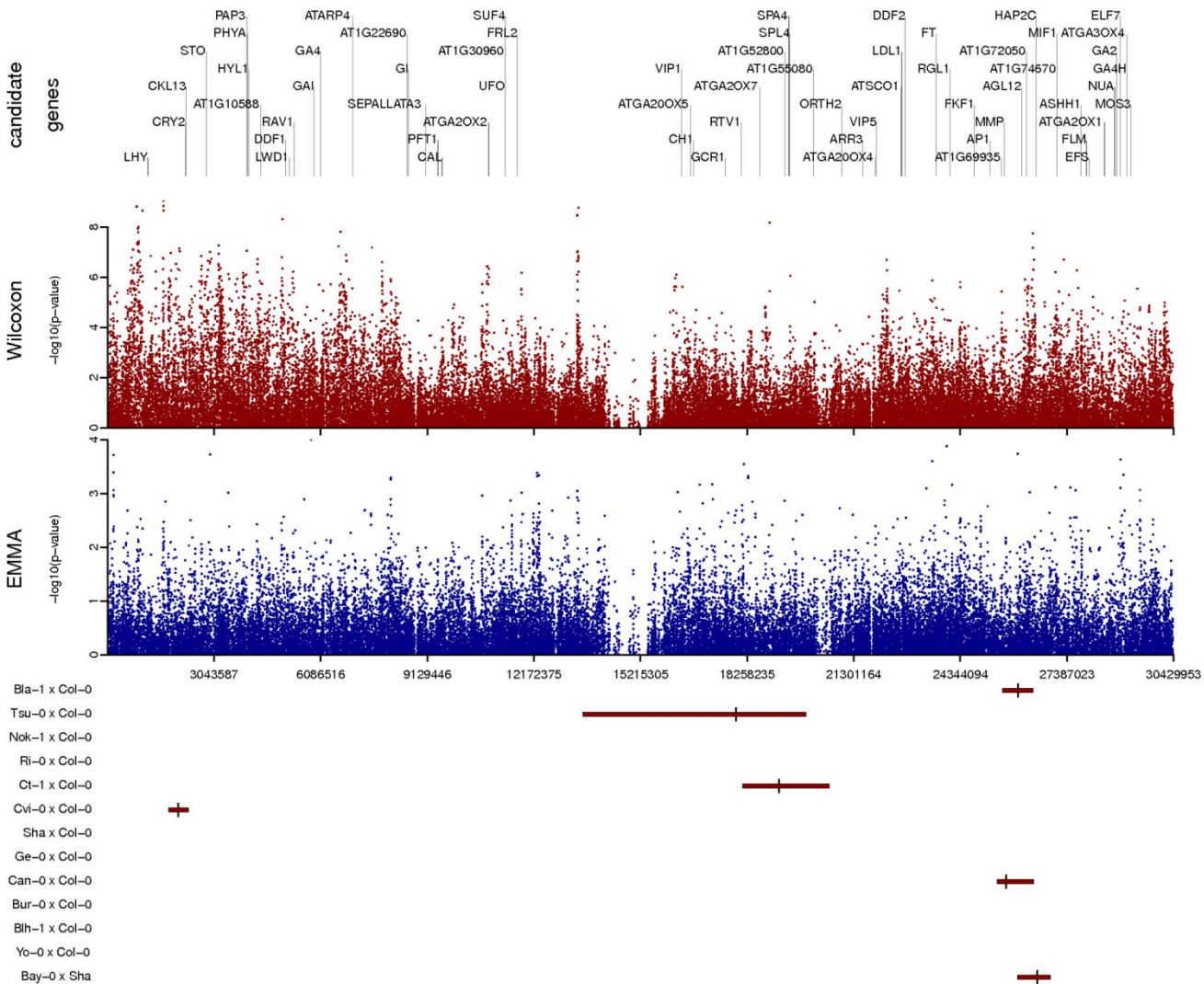

# Chromosome 4

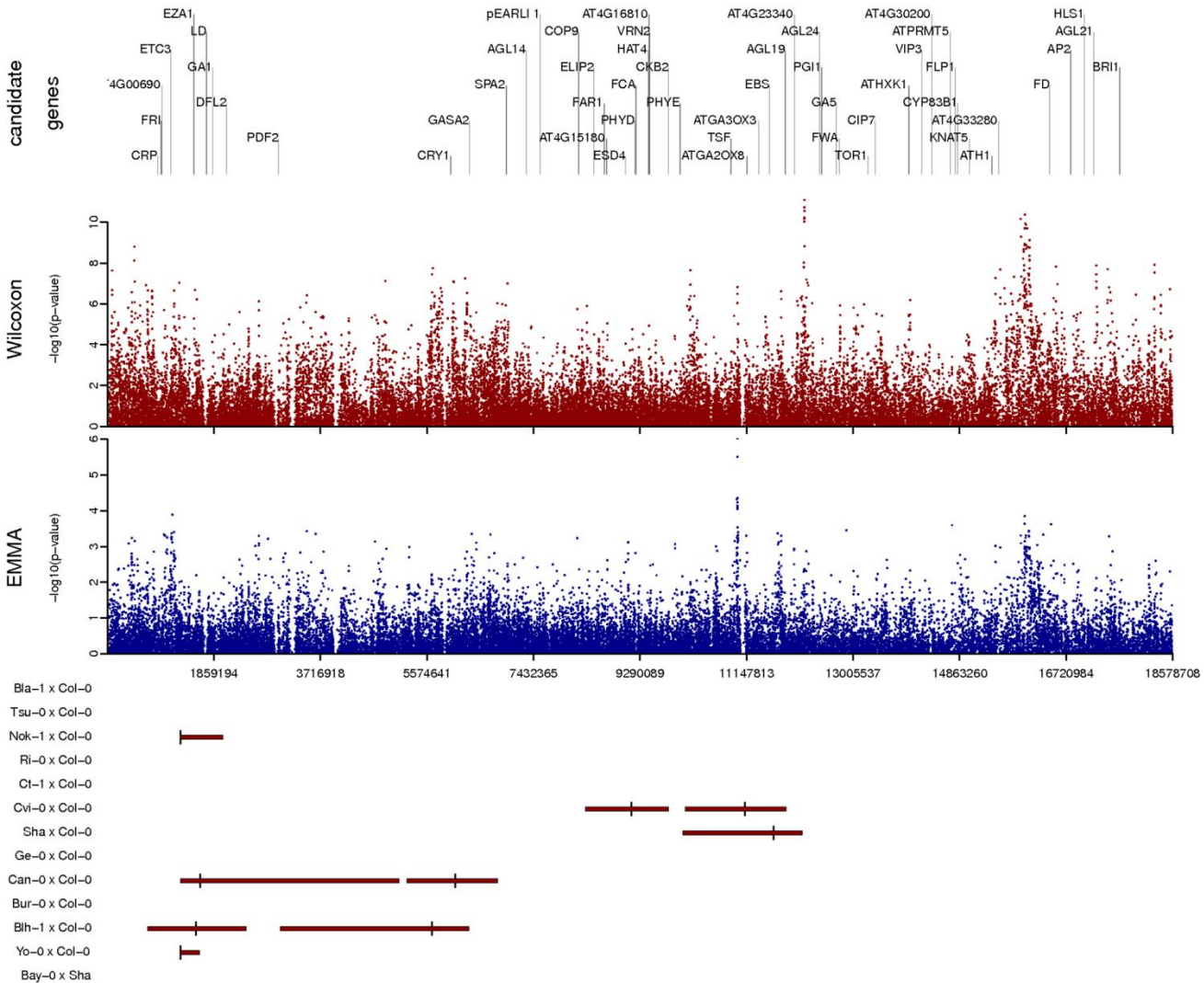

# Chromosome 5

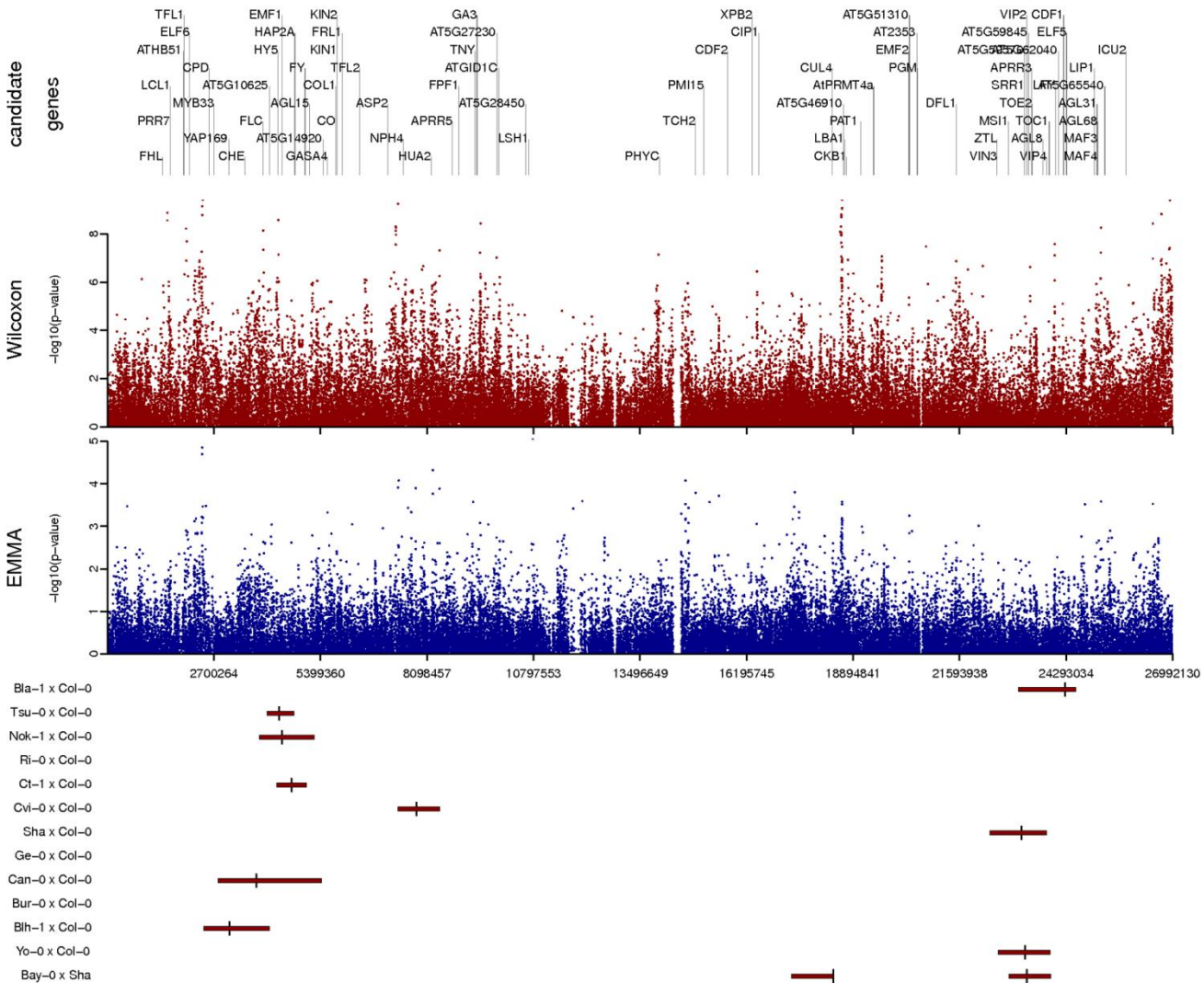

Supplement: Figure S9 — Comparison of GWA and traditional linkage mapping (epistatic QTLs) results for flowering time for chromosomes 1, 4, and 5. No epistatic QTLs were found on chromosomes 2 and 3. (0.77 MB PDF) [file pgen.1000940.s010.pdf]

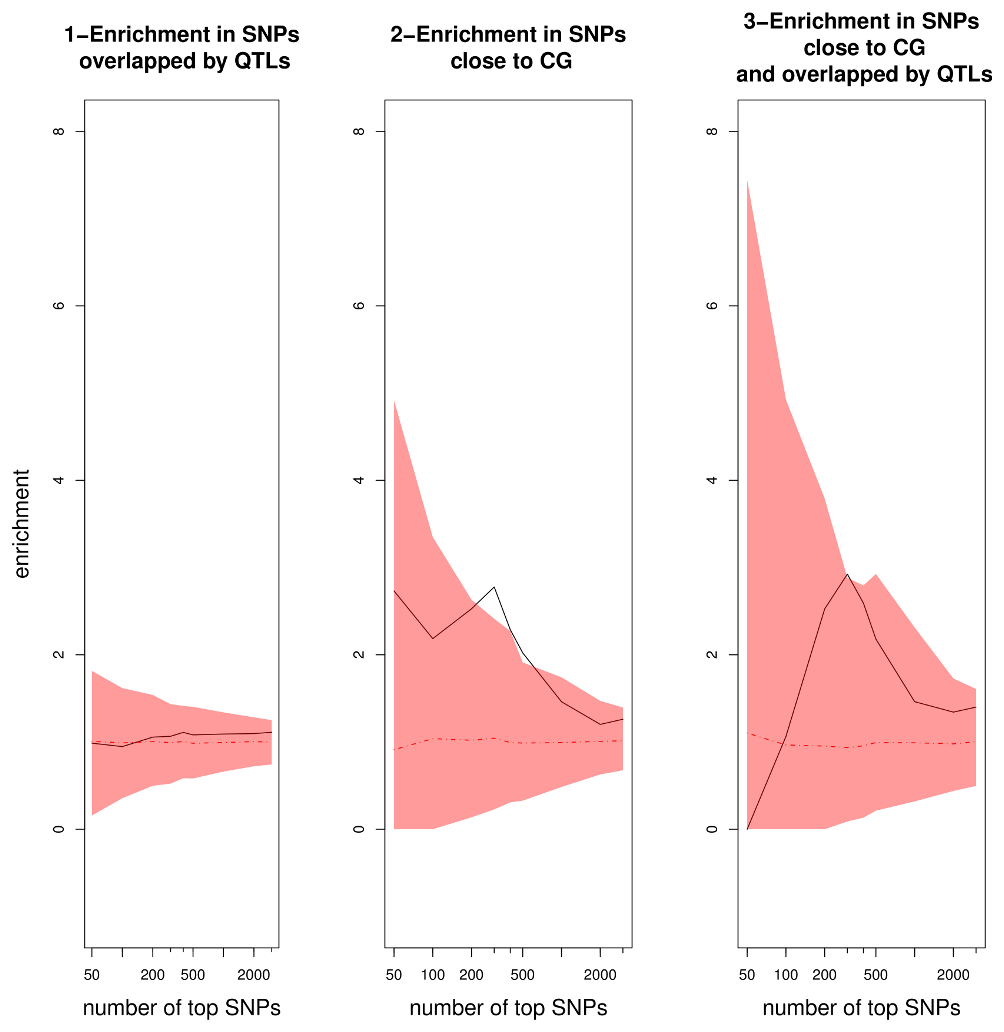

Supplement: Figure S10 — Enrichment ratios as a function of the number of top SNPs chosen in the GWA mapping results using the Wilcoxon test. The mean and the corresponding 95% confidence interval from the null distributions are represented by the dotted line and the colored areas, respectively. CG: candidate gene. (0.10 MB TIF) [file pgen.1000940.s011.tif]
