# Supplementary material for: Linkage and Association Mapping of Arabidopsis thaliana Flowering Time in Nature
Source: PLoS Genet. 2010 May 6;6(5):e1000940. doi: 10.1371/journal.pgen.1000940 (PMC2865524; doi:10.1371/journal.pgen.1000940)
Supplement: Figure S5 — Network of additive and epistatic QTLs for flowering time for each RIL family. (0.38 MB PDF) [file pgen.1000940.s006.pdf]

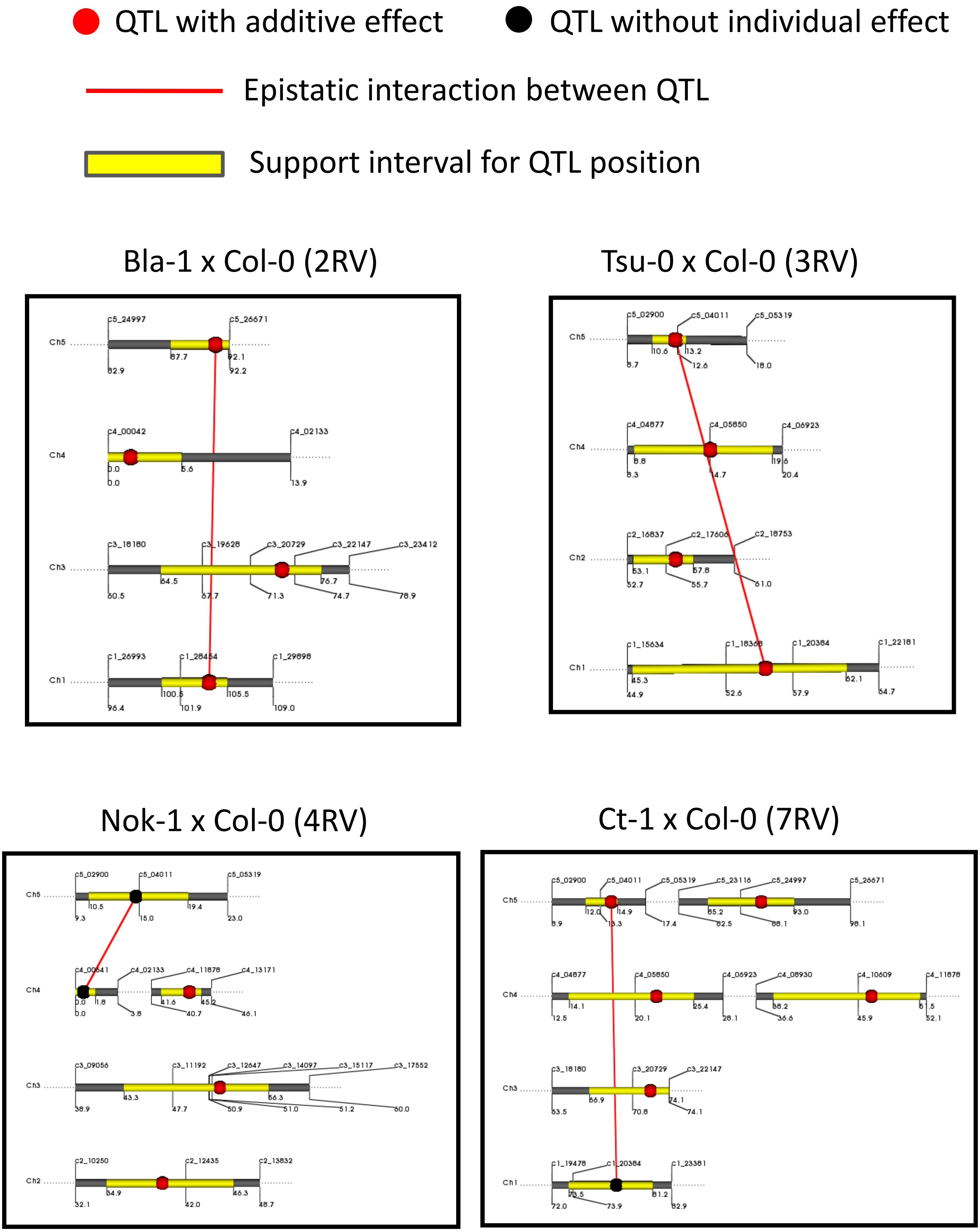

QTL with additive effect
QTL without individual effect
Epistatic interaction between QTL
Support interval for QTL position
Bla-1 x Col-0 (2RV)
Ch5
c5_24997
c5_26671
87.7
92.1
82.9
92.2
Ch4
c4_00042
c4_02133
0.0
5.6
0.0
13.9
Ch3
c3_18180
c3_19628
c3_20729
c3_22147
c3_23412
64.5
76.7
60.5
67.7
71.3
74.7
78.9
Ch1
c1_26993
c1_28454
c1_29898
100.5
105.5
96.4
101.9
109.0
Tsu-0 x Col-0 (3RV)
Ch5
c5_02900
c5_04011
c5_05319
10.6
13.2
8.7
12.6
18.0
Ch4
c4_04877
c4_05850
c4_06923
8.8
19.6
8.3
14.7
20.4
Ch2
c2_16837
c2_17606
c2_18753
53.1
57.8
52.7
55.7
61.0
Ch1
c1_15634
c1_18368
c1_20384
c1_22181
45.3
62.1
44.9
52.6
57.9
64.7
Nok-1 x Col-0 (4RV)
Ch5
c5_02900
c5_04011
c5_05319
10.5
19.4
9.3
15.0
23.0
Ch4
c4_00641
c4_02133
c4_11878
c4_13171
0.0
1.8
41.6
45.2
0.0
3.8
40.7
46.1
Ch3
c3_09056
c3_11192
c3_12647
c3_14097
c3_15117
c3_17552
43.3
56.3
38.9
47.7
50.9
51.0
51.2
60.0
Ch2
c2_10250
c2_12435
c2_13832
34.9
46.3
32.1
42.0
48.7
Ct-1 x Col-0 (7RV)
Ch5
c5_02900
c5_04011
c5_05319
c5_23116
c5_24997
c5_26671
12.0
14.9
85.2
93.0
8.9
13.3
17.4
82.5
88.1
98.1
Ch4
c4_04877
c4_05850
c4_06923
c4_08930
c4_10609
c4_11878
14.1
25.4
38.2
51.5
12.5
20.1
28.1
36.6
45.9
52.1
Ch3
c3_18180
c3_20729
c3_22147
66.9
74.1
63.5
70.8
74.1
Ch1
c1_19478
c1_20384
c1_23381
73.5
81.2
72.0
73.9
82.9

Ri-0 x Col-0 (6RV)

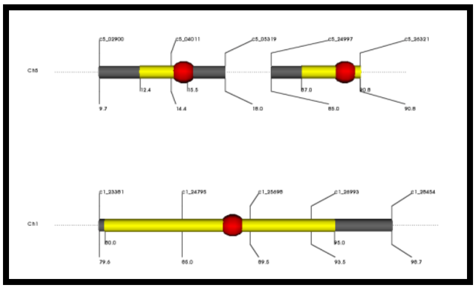

Cvi-0 x Col-0 (8RV)

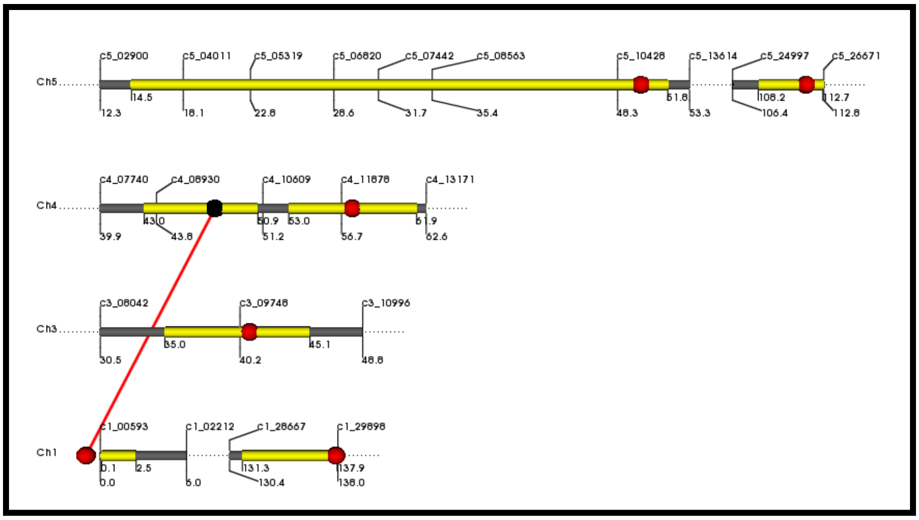

Ge-0 x Col-0 (17RV)

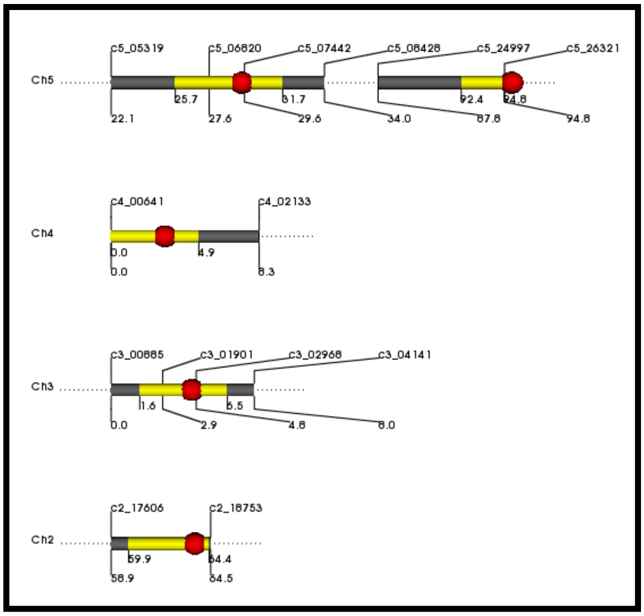

Shahdara x Col-0 (13RV)

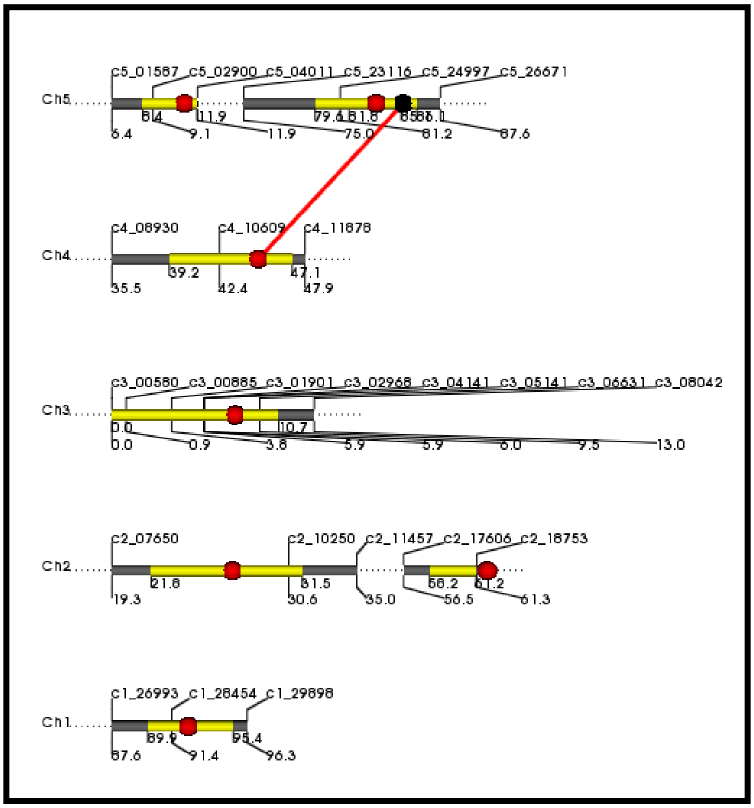

Yo-0 x Col-0 (23RV)

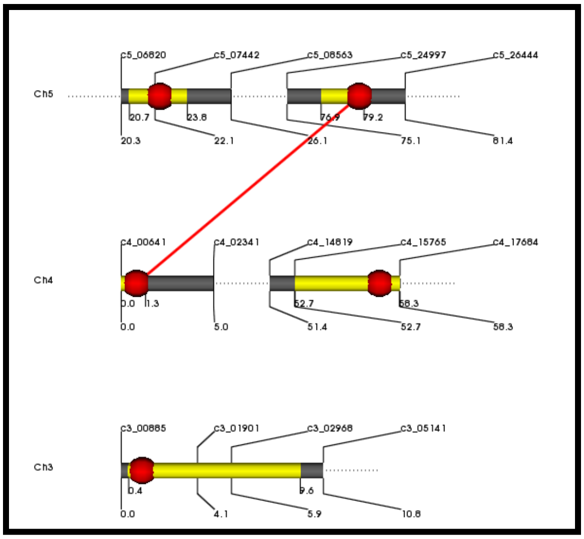

Blh-1 x Col-0 (21RV)

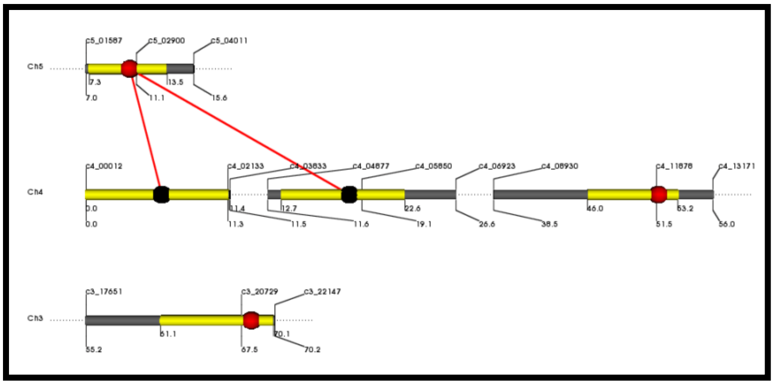

Can-0 x Col-0 (19RV)

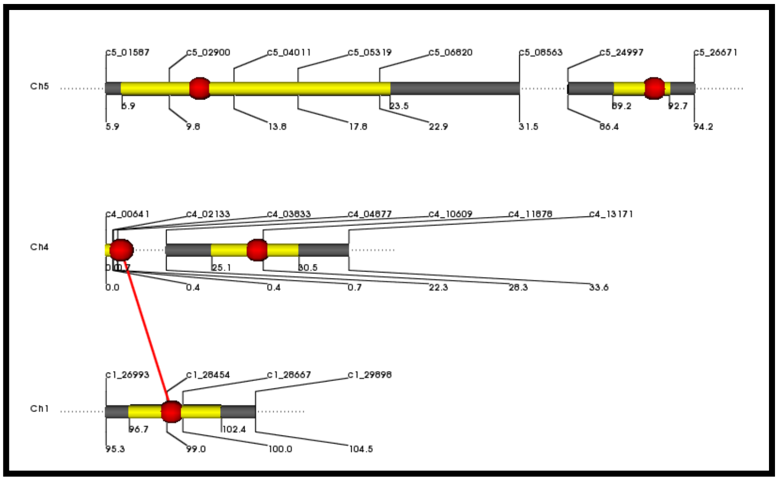

Bur-0 x Col-0 (20RV)

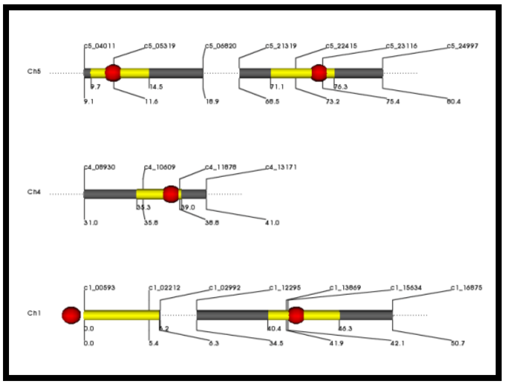

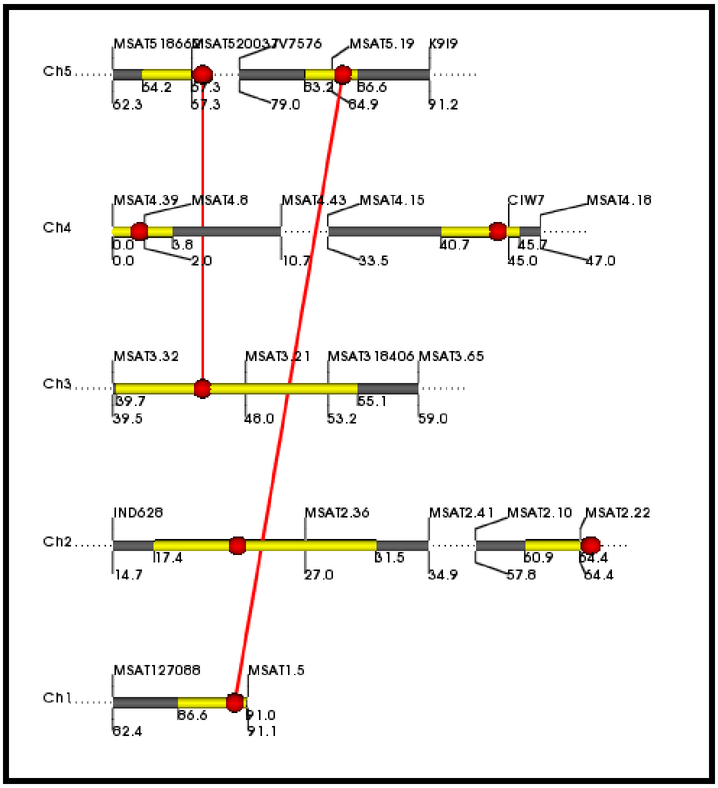

Bay-0 x Shahdara (33RV)
