# Supplementary material for: Linkage and Association Mapping of Arabidopsis thaliana Flowering Time in Nature
Source: PLoS Genet. 2010 May 6;6(5):e1000940. doi: 10.1371/journal.pgen.1000940 (PMC2865524; doi:10.1371/journal.pgen.1000940)
Supplement: Text S1 — Photothermal time. (0.05 MB PDF) [file pgen.1000940.s012.pdf]

Photothermal time:

Photothermal time acquisition required measurement of outside temperatures over the entire experiment from 24 September 2007 until mid-June 2008. From 24 September to 20 October 2007, plants were grown in the greenhouse and the temperature was recorded by the greenhouse internal sensor every 6 minutes. From 15 October to the end of the experiment, the temperature was obtained using the greenhouse external sensor, every 6 minutes, located close to the common garden where the experiment took place. Mean temperatures were calculated for every day, considering only the temperatures measured between dawn and sunset. The time of dawn and sunset as well as day length where obtained from the website www.meteofrance.com.
