# Supplementary material for: Linkage and Association Mapping of Arabidopsis thaliana Flowering Time in Nature
Source: PLoS Genet. 2010 May 6;6(5):e1000940. doi: 10.1371/journal.pgen.1000940 (PMC2865524; doi:10.1371/journal.pgen.1000940)
Supplement: Text S2 — Enrichment and null distribution computation. (0.01 MB PDF) [file pgen.1000940.s013.pdf]

Enrichment and null distribution computation:

Enrichment was calculated as the ratio between the proportion of SNPs close to candidate genes and/or overlapped by at least 1 QTL among SNPs above a defined threshold (i.e. number of top SNPs chosen) and the proportion of SNPs of the same category over the whole genome.

To test the significance of enrichment, we generated a null distribution using permutations. The algorithm used is detailed below:

- Lists of SNP positions and associated p-values for each chromosome were reordered, pasting the chromosomes in a random orientation.
- The same procedure was applied to the annotations (*i.e.* whether the SNP is close to a candidate gene and/or overlapped by at least 1 QTL).
- These 2 newly formed lists were rotated randomly with respect to one another.
- Enrichment in SNPs close to candidate genes and/or overlapped by QTLs was calculated for the defined number of top SNPs.

These 4 steps were repeated 500 times in order to produce the null distribution for each number of top SNPs to be tested. A 5% confidence threshold was then assessed from the null distribution.
